# Urinary multi-omics reveal non-invasive diagnostic biomarkers in clear cell renal cell carcinoma

**DOI:** 10.1038/s44321-026-00498-2

**Published:** 2026-08-11

**Authors:** Gustav Jonsson, Tiago Oliveira, Maura Hofmann, Ursula Lemberger, Karel Stejskal, Gabriela Krššáková, Irma Sakic, Maria Novatchkova, Stefan Mereiter, Gerlinde Grabmann, Thomas Köcher, Rubina Koglgruber, Zeljko Kikic, Sonia Camano Páez, Bárbara Luna Sanchez, Víctor Díez Nicolás, Gerald N Rechberger, Thomas Züllig, Astrid Hagelkruys, Bernhard Englinger, Manuela Schmidinger, Josef M Penninger

**Affiliations:** 1 Institute of Molecular Biotechnology of the Austrian Academy of Sciences, IMBA, Vienna, Austria; 2 Department of Laboratory Medicine, Medical University of Vienna, Vienna, Austria; 3 Vienna BioCenter PhD Program, Doctoral School of the University of Vienna and Medical University of Vienna, Vienna, Austria; 4 Department of Urology, Center for Cancer Research and Comprehensive Cancer Center, Medical University of Vienna, Vienna, Austria; 5 Vienna BioCenter Core Facilities (VBCF), Vienna, Austria; 6 BioBank Hospital Ramón y Cajal-IRYCIS, Madrid, Spain; 7 Urology Department, Ramón y Cajal Hospital, IRYCIS, Madrid, Spain; 8 Institute of Molecular Biosciences, NAWI Graz, University of Graz, Graz, Austria; 9 Department of Medical Genetics, Life Sciences Institute, University of British Columbia, Vancouver, BC Canada; 10 Helmholtz Centre for Infection Research, Braunschweig, Germany

**Keywords:** Biomarkers, Cancer, Urogenital System

## Abstract

Clear cell renal cell carcinoma (ccRCC) is the most common kidney malignancy. Yet, no rapid, non-invasive biomarkers are available for diagnosis or screening. Urine represents an ideal analyte matrix due to its accessibility, low invasiveness, longitudinal sampling, and the kidney’s central role in filtration. Here, we integrated proteomic, lipidomic, and metabolomic analyses of urine from ccRCC patients and controls to identify diagnostic biomarkers. Multi-omics profiling revealed urogenital metabolic dysregulation in ccRCC, including increased lipid metabolism, altered mitochondrial respiration signatures, and elevated urinary lipid content. We identified three urinary protein biomarkers: serum amyloid A1 (SAA1), haptoglobin (HP), and lipocalin 15 (LCN15). Using a parallel reaction monitoring mass spectrometry workflow, we developed a rapid and sensitive assay and combined these markers into a diagnostic UrineScore. The UrineScore achieved 0.96 in an area under the receiver operating characteristic curve analysis in the discovery cohort, and 0.95 in an independent validation cohort. Together, these results support the feasibility of multi-omics-guided urinary biomarker discovery and represent a step toward accessible diagnostic platforms for ccRCC.

## Synopsis

Urine multi-omics profiling of ccRCC patients identifies a three-protein diagnostic signature (HP, SAA1, and LCN15) that forms a composite UrineScore with an AUROC of 96% in distinguishing clear cell renal cell carcinoma from healthy controls, validated in an independent cohort.

- Integrated urine proteomics, lipidomics, and metabolomics identified widespread metabolic dysregulation in ccRCC.
- Three urinary proteins - Haptoglobin (HP), Serum Amyloid A1 (SAA1), and Lipocalin 15 (LCN15) - were identified as specifically elevated in ccRCC compared with healthy controls and non-clear cell RCC subtypes.
- A targeted parallel reaction monitoring mass spectrometry (PRM-MS) assay quantified HP, SAA1, and LCN15, and the resulting UrineScore achieved AUROCs of 0.96 and 0.95 in the discovery and validation cohorts, respectively.
- Tumor-specific upregulation of HP, SAA1, and LCN15 was confirmed in TCGA RNA-sequencing data and CPTAC tissue proteomics; combined high expression predicted worse overall survival in ccRCC.

## Abstract (web summary)

Urine multi-omics profiling of ccRCC patients identifies a three-protein diagnostic signature (HP, SAA1, and LCN15) that forms a composite UrineScore with an AUROC of 96% in distinguishing clear cell renal cell carcinoma from healthy controls, validated in an independent cohort.

## Graphical abstract

**Figure:**
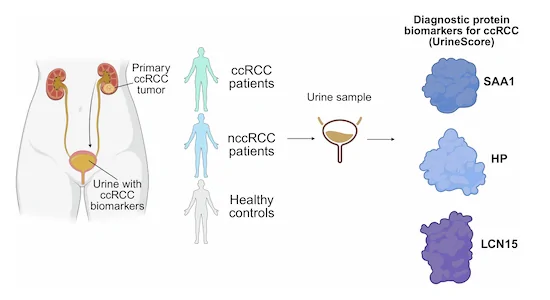

The paper explained **Problem**

Clear cell renal cell carcinoma (ccRCC) is the most common subtype of kidney cancer, accounting for ~80% of all kidney cancer cases. Despite a growing global incidence exceeding 430,000 new diagnoses annually, no validated molecular biomarker exists for the diagnosis or monitoring of ccRCC in routine clinical practice. Patients are most often identified incidentally through abdominal imaging or at late stages when symptoms such as hematuria become apparent, at which point the prognosis is substantially worse. Unlike many other solid tumors, ccRCC has no approved liquid biopsy test, leaving a critical diagnostic gap. The kidney’s central role in blood filtration makes urine an anatomically privileged matrix for biomarker discovery: proteins shed by tumors in the proximal tubule are concentrated directly into the excreted compartment. Despite this, multi-omics characterization of ccRCC urine and its translation into a validated diagnostic assay are still lacking.

**Results**

Integrated proteomics, lipidomics, and metabolomics of urine from ccRCC patients and matched controls revealed widespread metabolic dysregulation. Three urinary proteins, Haptoglobin (HP), Serum Amyloid A1 (SAA1), and Lipocalin-15 (LCN15), were identified as specifically elevated in ccRCC urine compared to both healthy controls and other types of kidney cancer. A targeted parallel reaction monitoring mass spectrometry assay was developed for rapid, sensitive quantification of these three markers, and their measurements combined into a composite UrineScore. The UrineScore achieved 96% accuracy in the discovery cohort and 95% accuracy in an independent validation cohort. Tumor-specific upregulation of HP, SAA1, and LCN15 was further corroborated in publicly available datasets, where combined high expression was associated with worse overall survival in ccRCC.

**Impact**

The UrineScore represents a proof-of-concept for non-invasive, urine-based molecular diagnosis of ccRCC, a disease that currently has no equivalent clinical tool. The assay is based on three defined protein analytes measurable by mass spectrometry infrastructure already present in academic medical centers and is, in principle, adaptable to antibody-based formats such as ELISA or lateral flow assays suitable for routine laboratories. Validation in larger, clinically representative cohorts, including patients with benign renal masses and other urological conditions, is the essential next step. If confirmed, a urine-based diagnostic of this kind could be incorporated into existing clinical check-up workflows, enabling earlier detection in the large population of patients currently diagnosed only after incidental imaging or symptomatic presentation.

## Introduction

Renal cell carcinomas (RCCs) are a heterogenous group of cancers arising from various epithelial subsets of the kidney, such as the proximal convoluted tubules, or the distal nephron and collecting ducts, depending on the subtype. Many histological subtypes of renal cell carcinomas have been described, with the three most common ones being clear cell renal cell carcinoma (ccRCC), papillary renal cell carcinoma (pRCC), and chromophobe renal cell carcinoma (chRCC). Among these three subtypes, ccRCC is the most common, making up ~80% of all RCC cases; ccRCC is also the deadliest of these three subtypes (Hsieh et al, 2017; Muglia and Prando, 2015). ccRCC is primarily driven by loss-of-function mutations or epigenetic silencing of the *Von Hippel Lindau (VHL)* tumor suppressor, leading to the initiation of a genetic hypoxia program which drives tumor progression (Giaccia et al, 2003; Lonser et al, 2003). Despite this clearly identified key mechanism, solely screening for *VHL* mutations for disease detection is not sufficient since not all patients present with *VHL* gene mutations. Furthermore, multiple additional co-drivers have also been identified, such as PBRM1, SETD2, KDM5C, or BAP1 (Sato et al, 2013; Hakimi et al, 2013), confounding genetic-based disease testing.

Despite significant advances in the treatment of ccRCC using small molecule inhibitors and immunotherapies (Albiges et al, 2023; Powles et al, 2022; Vano et al, 2022), early screenable markers for diagnosis are still lacking and most patients are diagnosed through routine imaging when a tumor is incidentally found or already suspected, for example due to palpable renal masses or hematuria (Gray and Harris, 2019; Rose and Kim, 2024). Hematuria is a very common sign of ccRCC and other renal cell malignancies, but is in and of itself insufficient for a reliable diagnosis. The proximal convoluted tubule, where ccRCC arises, is responsible for secreting and reabsorbing solutes between blood and urine (Burg et al, 1976), increasing the likelihood that secreted or shed biomarkers are present and detectable in the urine. Urine also allows longitudinal sampling and is minimally invasive. Omics approaches have been used on urine samples from renal cell carcinomas in the past for the purpose of biomarker discovery, predominantly metabolomics due to the natural abundance of high levels of excreted metabolites as waste products in urine (Lucarelli et al, 2019). However, multiple omics approaches have yet to be integrated on the same urine samples to draw multi-modal conclusions about the dysregulation of the urinary landscape in ccRCC, and how such knowledge can be exploited for biomarker discovery.

In the present work, by combining proteomics, lipidomics, and metabolomics, we detected urogenital metabolic dysregulation in ccRCC patients defined by increased lipid metabolism, altered mitochondrial respiration signatures, and increased urinary lipid content. We further aimed to explore urinary protein biomarkers to reliably detect ccRCC. In clinical cohorts of healthy controls and ccRCC, we discovered and validated three proteins as potential ccRCC diagnostic biomarkers: SAA1, HP, and LCN15. We further developed a parallel reaction monitoring mass spectrometry (PRM-MS) signature for rapid and sensitive quantitative detection of these three marker proteins in patient urine, discriminating between controls and ccRCC patients with a performance accuracy of 96%. This diagnostic signature was validated in an independent ccRCC patient cohort and was further corroborated through computational analysis of publicly available cohorts. Our work serves as a step towards further development of urine-based diagnostics in renal malignancy.

## Results

### Urine from ccRCC patients is indicative of metabolic dysregulation

**Figure EV1:**
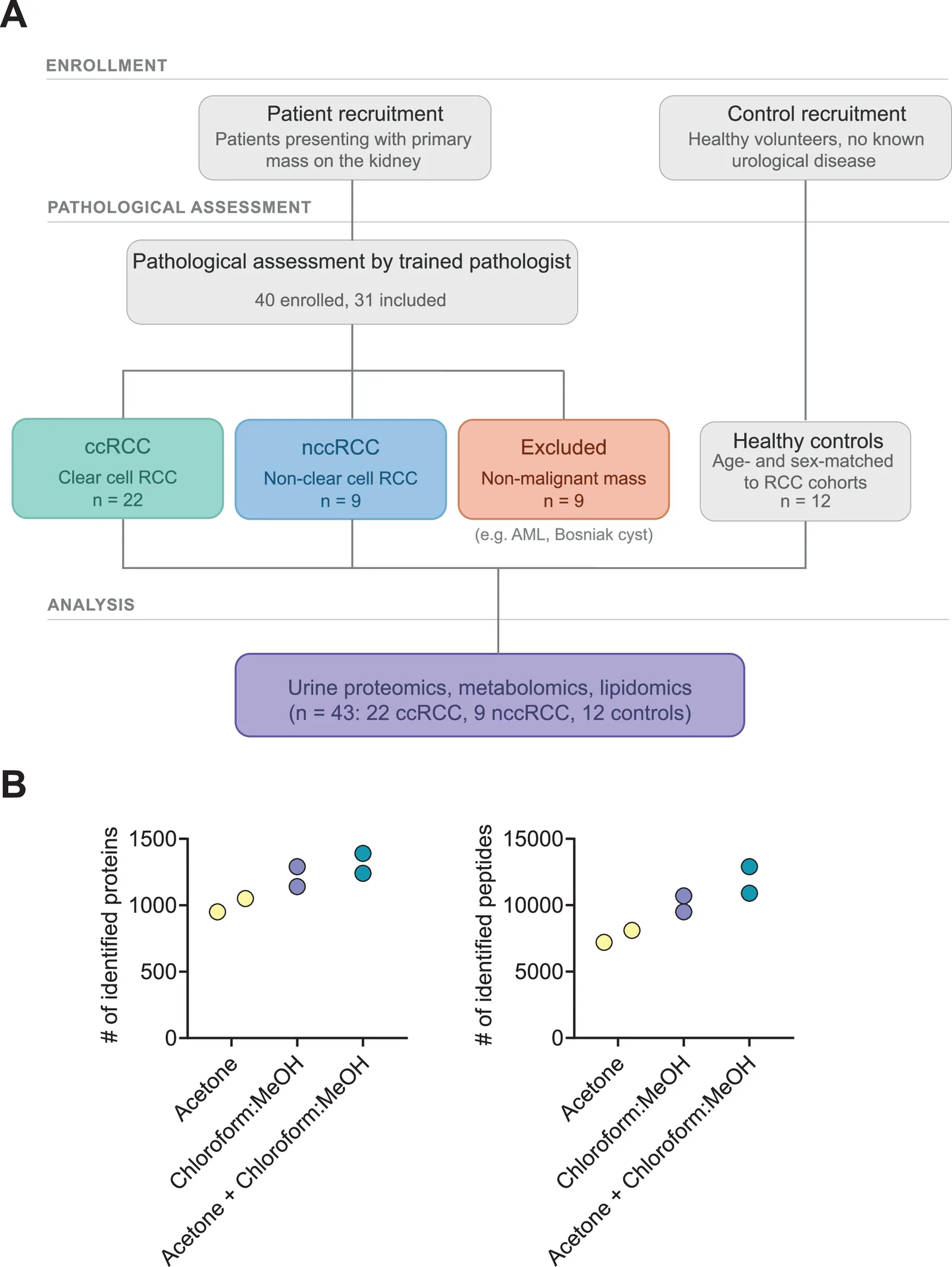
Discovery cohort patient recruitment and urine precipitation. (**A**) Flowchart describing the recruitment of patients and controls into the discovery cohort. Urine was collected from 40 patients presenting in the clinic with a foreign kidney mass. After pathological assessment, 22 patients were assigned to the ccRCC group and 9 patients to the nccRCC group, 9 patients were excluded due to the masses being non-malignant, e.g., cysts. In parallel, 12 healthy age-matched controls were recruited. ccRCC clear cell renal cell carcinoma, nccRCC non-clear cell renal cell carcinoma. (**B**) Number of identified unique proteins (left) and unique peptides (right) in a healthy control urine supernatant sample using three different protein precipitation methods. (i) Precipitation only with acetone, (ii) precipitation only with chloroform and methanol, and (iii) double protein precipitation with acetone followed by chloroform and methanol. Two technical replicates per sample is shown. MeOH Methanol.

**Figure 1:**
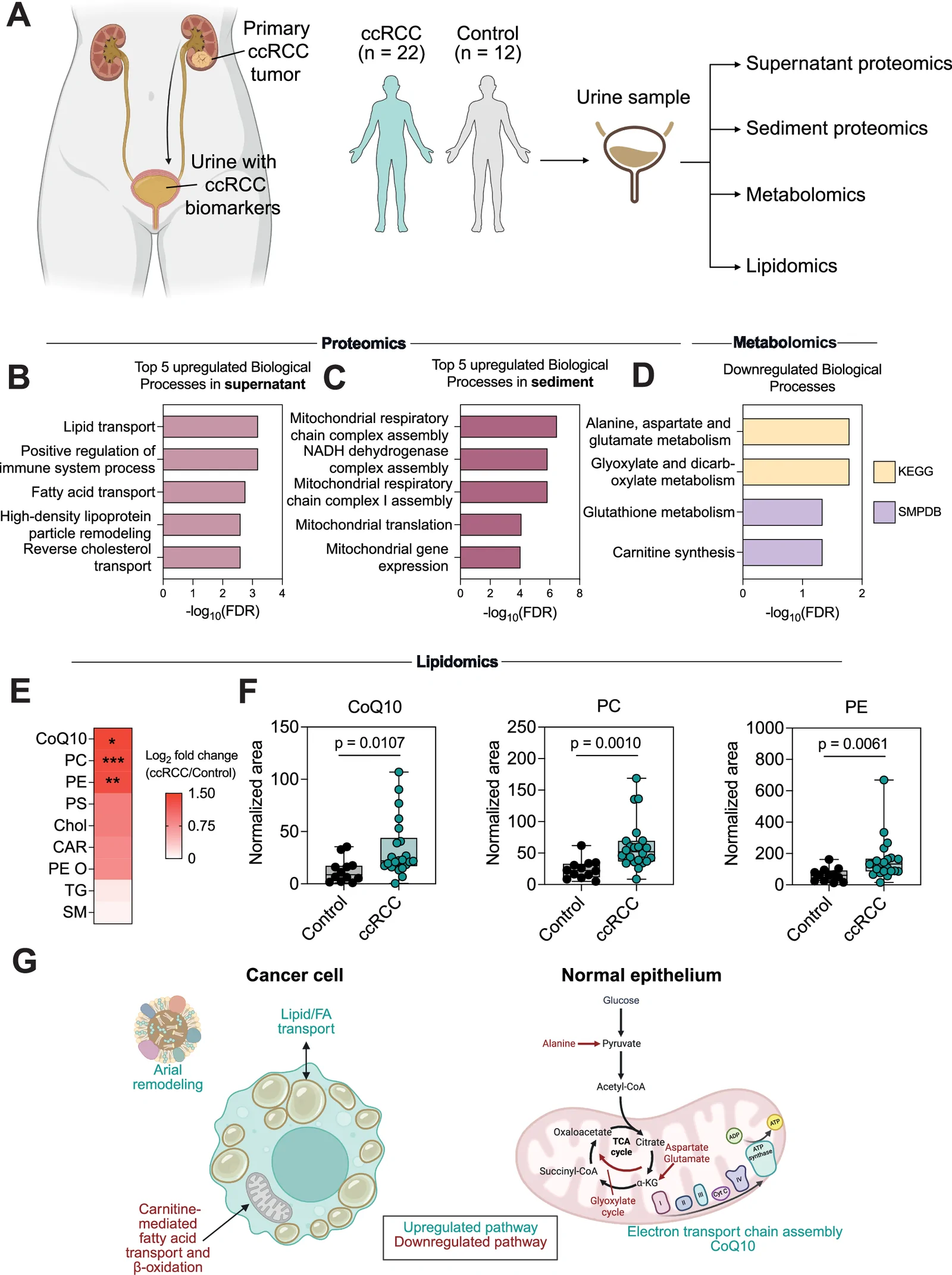
Multi-omics analysis of ccRCC patient urine indicates metabolic dysregulation. (**A**) Schematic of the detection of urine analytes in ccRCC patients passing through the urogenital tract and accumulating in the urinary bladder before discharge in a ccRCC patient cohort. (**B**) Gene ontology (GO) term analysis using Enrichr on all upregulated proteins in ccRCC urine (*n* = 22) supernatant compared to Control urine (*n* = 12) supernatant. FDR false discovery rate. (**C**) Gene ontology (GO) term analysis using Enrichr on all upregulated proteins in ccRCC urine (*n* = 21, the protein amounts were too low in 1 ccRCC sample for proper measurements) sediment compared to Control urine (*n* = 8, the protein amounts were too small in 4 control samples for proper measurements) sediment. FDR false discovery rate. (**D**) Gene ontology (GO) term analysis using Metaboanalyst on all downregulated metabolites in ccRCC urine (*n* = 22) supernatant compared to Control urine (*n* = 12) supernatant. The downregulated metabolites were matched against two different databases: KEGG and SMPDB. FDR false discovery rate. (**E**) Heatmap showing fold change between the detected lipid families in ccRCC patient urine (*n* = 22) and control urine (*n* = 12). CoQ10 coenzyme Q10, PC phosphatidylcholines, PE phosphatidylethanolamine, PS phosphatidylserine, Chol cholesterol, CAR acylcarnitines, PE O ether-linked phosphatidylethanolamine, TG triglyceride, SM sphingomyelin. *P < 0.05, **P < 0.01, ***P < 0.001. Exact *P* values are provided in (**F**) for the significantly different lipid families. Statistical significance assessed through limma-moderated Benjamini–Hochberg-corrected two-sided *t* test. (**F**) Boxplot of individual values for the three significantly higher lipid families from (**E**) in ccRCC patient urine (*n* = 22) and control urine (*n* = 12). CoQ10 coenzyme Q10, PC phosphatidylcholines, PE phosphatidylethanolamine. Statistical significance assessed through limma-moderated Benjamini–Hochberg-corrected two-sided *t* test. The line in the middle of the box denotes the mean of the data, whereas the lower and upper boundaries of the box represent the 25th (Q1) and 75th (Q3) percentiles, respectively. (**G**) Schematic of proposed altered metabolic network in the urogenital tract of ccRCC patients. Green represents upregulated pathways and red represents downregulated pathways. HDL high-density lipoprotein, CoA coenzyme A, α-KG alpha-ketoglutarate, CoQ10 coenzyme Q10. Source data are available online for this figure.

To characterize the metabolic landscape of ccRCC and to potentially derive urine biomarkers for the disease we initially performed proteomics on urine sediments and supernatants on a discovery cohort of ccRCC patients and age- and gender-matched controls (Fig. EV1A and Table EV1) recruited at the Vienna General Hospital (Fig. 1A). In pilot studies we found that precipitating proteins in two steps from the urine samples (tested on supernatant) starting with an acetone precipitation followed by a chloroform:methanol precipitation yielded the highest number of unique peptides and proteins identified (Fig. EV1B). This method was subsequently used for all protein preparations. All discovery-phase proteomics analysis was done using data-independent acquisition (DIA). Gene ontology (GO) term analysis of all upregulated proteins in ccRCC urine supernatants from proteomics, compared to controls, predominantly revealed metabolic dysregulation affecting lipid metabolism and function through fatty acid transport and high-density lipoprotein particle remodeling (Fig. 1B; Dataset EV1). Further, GO term analysis on sediment proteomics from control and ccRCC patient urine samples showed that mitochondrial respiration and electron transport processes were upregulated in the urine of ccRCC patients (Fig. 1C; Dataset EV2).

**Figure EV2:**
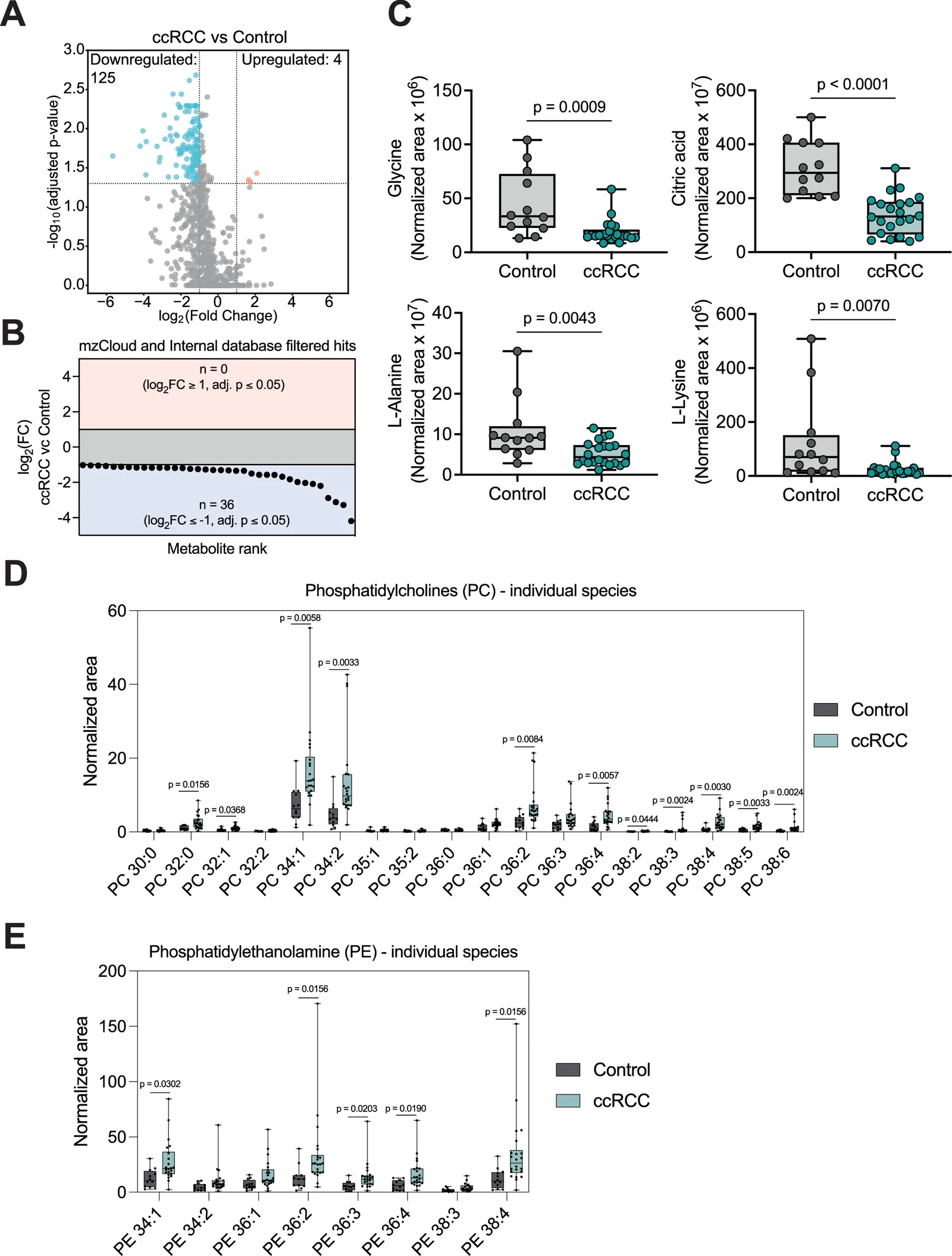
Metabolites and lipids in the urine of ccRCC patients. (**A**) Volcano plot showing upregulated (red) and downregulated (blue) metabolites in ccRCC (*n* = 22) patients compared to controls (*n* = 12). Adjusted *P* values calculated via limma-moderated Benjamini–Hochberg-corrected two-sided *t* test. FC fold change. (**B**) Waterfall plot of significantly metabolites remaining when comparing control (*n* = 12) and ccRCC (*n* = 22) urine after filtering all hits from (**A**) through the mzCloud and an internal database. 36 significantly downregulated metabolites remain. (**C**) Boxplots showing the expression levels of 4 representative metabolites (glycine, citric acid, L-alanine, and L-lysine), as determined by metabolomics for controls (*n* = 12) and ccRCC (*n* = 22). Statistical significance assessed through limma-moderated Benjamini–Hochberg-corrected two-sided *t* test. The line in the middle of the box denotes the mean of the data, whereas the lower and upper boundaries of the box represent the 25th (Q1) and 75th (Q3) percentiles, respectively. (**D**) Boxplots of individual lipid species from the PC family for controls (*n* = 12) and ccRCC (*n* = 22). PC phosphatidylcholines. *P < 0.05, **P < 0.01. Statistical significance assessed through limma-moderated Benjamini–Hochberg-corrected two-sided *t* test. The line in the middle of the box denotes the mean of the data, whereas the lower and upper boundaries of the box represent the 25th (Q1) and 75th (Q3) percentiles, respectively. (**E**) Boxplots of individual lipid species from the PE family for controls (*n* = 12) and ccRCC (*n* = 22). PE phosphatidylethanolamine. *P < 0.05. Statistical significance assessed through limma-moderated Benjamini–Hochberg-corrected two-sided *t* test. The line in the middle of the box denotes the mean of the data, whereas the lower and upper boundaries of the box represent the 25th (Q1) and 75th (Q3) percentiles, respectively.

Since the proteomics data indicated dysregulated metabolism throughout the urogenital tract, we further performed metabolomics and lipidomics on the urine samples. Untargeted metabolomics predominantly revealed downregulated metabolites in ccRCC patients (Fig. EV2A). After filtering the hits further to only include metabolites from the mzCloud database and our in-house validated database, 36 downregulated metabolites remained (Fig. EV2B; Dataset EV3), some examples of which are shown in Fig. EV2C. GO term analysis on the 36 downregulated metabolites, matched against the KEGG and SMPDB databases, revealed downregulation of (i) aspartate, alanine and glutamate metabolism, (ii) glyoxylate metabolism, (iii) glutathione metabolism and (iv) carnitine synthesis (Fig. 1D). Lipidomics revealed that 3 out of 9 detected lipid classes were significantly upregulated in ccRCC compared to controls: Coenzyme Q10 (CoQ10), phosphatidylcholines (PCs) and phosphatidylethanolamines (PEs) (Fig. 1E,F; Dataset EV4). Combined, most of the measured individual PC and PE species were significantly upregulated in ccRCC compared to controls (Fig. EV2D,E).

In summary, the proteomics, metabolomics, and lipidomics data indicate dysregulated metabolism with increased electron transport chain activity and increased lipid metabolism throughout the urogenital tract. Since ccRCC cells are known to be lipid-laden and accumulate high levels of lipids, increased PC and PE levels in the urine likely come from increased lipid transport in these cells. ccRCC is also known to be independent of oxidative phosphorylation (Zhang et al, 2021), indicating that upregulated respiratory chain pathways in the urine sediment most likely originate from increased shedding of healthy epithelium or other shed urogenital cells. These scenarios are summarized in Fig. 1G.

### Lipidomics and metabolomics provide putative diagnostic biomarkers for ccRCC

**Figure 2:**
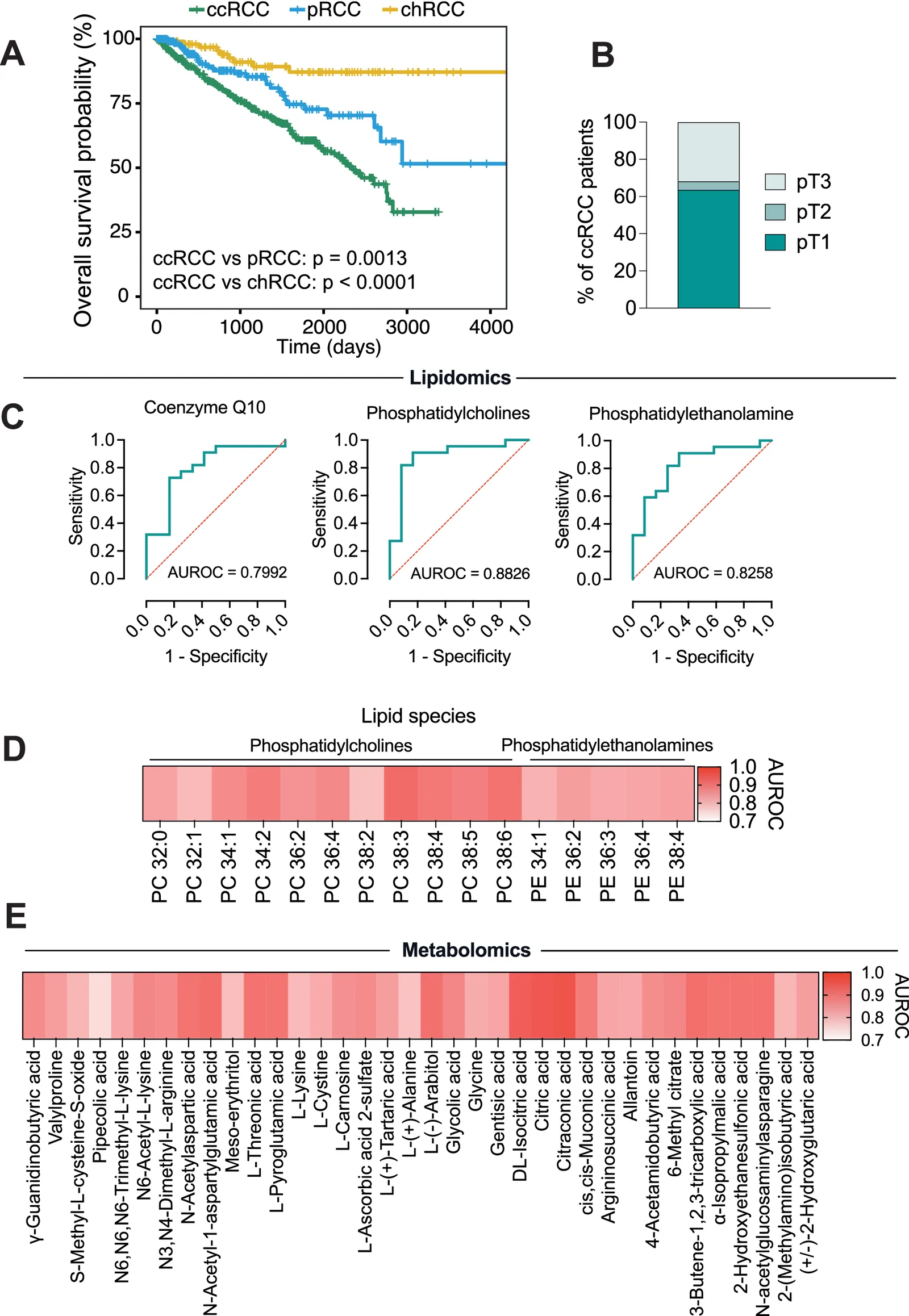
Lipidomics and metabolomics reveal biomarkers that distinguish between controls and ccRCC patients. (**A**) Overall survival curves for ccRCC (*n* = 537), pRCC (*n* = 287), and chRCC (*n* = 65) patients present in the TCGA pan-kidney database. TCGA The Cancer Genome Atlas, ccRCC clear cell renal cell carcinomas, pRCC papillary renal cell carcinomas, chRCC chromophobe renal cell carcinoma. Statistical significance was assessed through Log-rank tests. (**B**) Stacked barplot showing the distribution of tumor stages at the time of presentation in the clinic for ccRCC patients. pT1 = tumor stage 1 (*n* = 13). The tumor is a maximum of 7 cm across. pT2 =  tumor stage 2 (*n* = 1). The tumor is larger than 7 cm across. pT3 = tumor stage 3 (*n* = 8). The tumor has grown into a major renal vein (e.g., vena cava or renal vein) or into neighboring tissues but has not spread past Gerota’s fascia or into the adrenal gland. (**C**) Assessment of the ability of the three significantly upregulated lipid families (CoQ10, PC, and PE, respectively) in distinguishing between ccRCC (*n* = 22) and control (*n* = 12) cases using area under the receiver operating curve (AUROC) analysis. CoQ10 coenzyme Q10, PC phosphatidylcholines, PE phosphatidylethanolamine, AUROC area under the receiver operating characteristic curve. (**D**) Heatmap showing AUROC values for individual lipid species from PC and PE that were upregulated in ccRCC (*n* = 22) compared to controls (*n* = 12). PC phosphatidylcholines, PE phosphatidylethanolamine, AUROC area under the receiver operating characteristic curve. (**E**) Heatmap showing AUROC values for individual metabolites that were downregulated in ccRCC (*n* = 22) compared to controls (*n* = 12). Source data are available online for this figure.

ccRCC is the deadliest type of renal carcinoma amongst the most common subtypes with a tendency to appear asymptomatic at early stages leading to complications at the later stages of disease. Survival analysis of the three most common RCCs (ccRCC, pRCC, and chRCC) in TCGA confirmed the poor survival of ccRCC (Fig. 2A). Compared to many other malignancies, ccRCC lacks liquid biopsy biomarkers for early diagnosis and prognosis (Li et al, 2023; Farber et al, 2017). We hypothesized that our multi-omics dataset of urine from ccRCC patients (Fig. 2B) could enable the identification of diagnostic biomarkers, leveraging the non-invasive nature of urine collection and the multi-compartment proteomic and lipidomic profiling approach. Initially, we assessed the ability of the three significantly upregulated lipid classes (CoQ10, PC, and PE) to distinguish between ccRCC and controls in an area under the receiver operating characteristic curve (AUROC) analysis (Fig. 2C). CoQ10, PC, and PE all had a good performance of AUROC = 0.7992, AUROC = 0.8826, and AUROC = 0.8258, respectively, in distinguishing between ccRCC and control cases. Furthermore, when we assessed all significantly upregulated individual lipid species, all of them had an AUROC > 0.7, with PC 38:3 and PC 38:6 having the highest AUROC of 0.9129 and 0.8996, respectively (Fig. 2D), indicating the potential use of PC family members as biomarkers in ccRCC diagnosis.

Similar trends were observed when assessing downregulated metabolites. All significantly downregulated metabolites presented with an AUROC > 0.7 to distinguish between ccRCC and controls, with citraconic acid, citric acid, and DL-Isocitric acid scoring the highest AUROC values of 0.9545, 0.9432, and 0.9356, respectively (Fig. 2E). These data indicate that altered lipid and metabolite signatures could be used as biomarkers to distinguish ccRCC from healthy controls in urine.

### Proteomics provides high-confidence diagnostic biomarkers for ccRCC

Due to the overall low levels of metabolites present in urine, metabolomics was not further considered for the purpose of diagnostic detection of ccRCC. We only detected significantly downregulated metabolites in ccRCC patients. Urine as a sample has low levels of analytes, and establishing a diagnosis on the downregulation of already lowly abundant analytes poses additional challenges, despite the promising performance.

**Figure 3:**
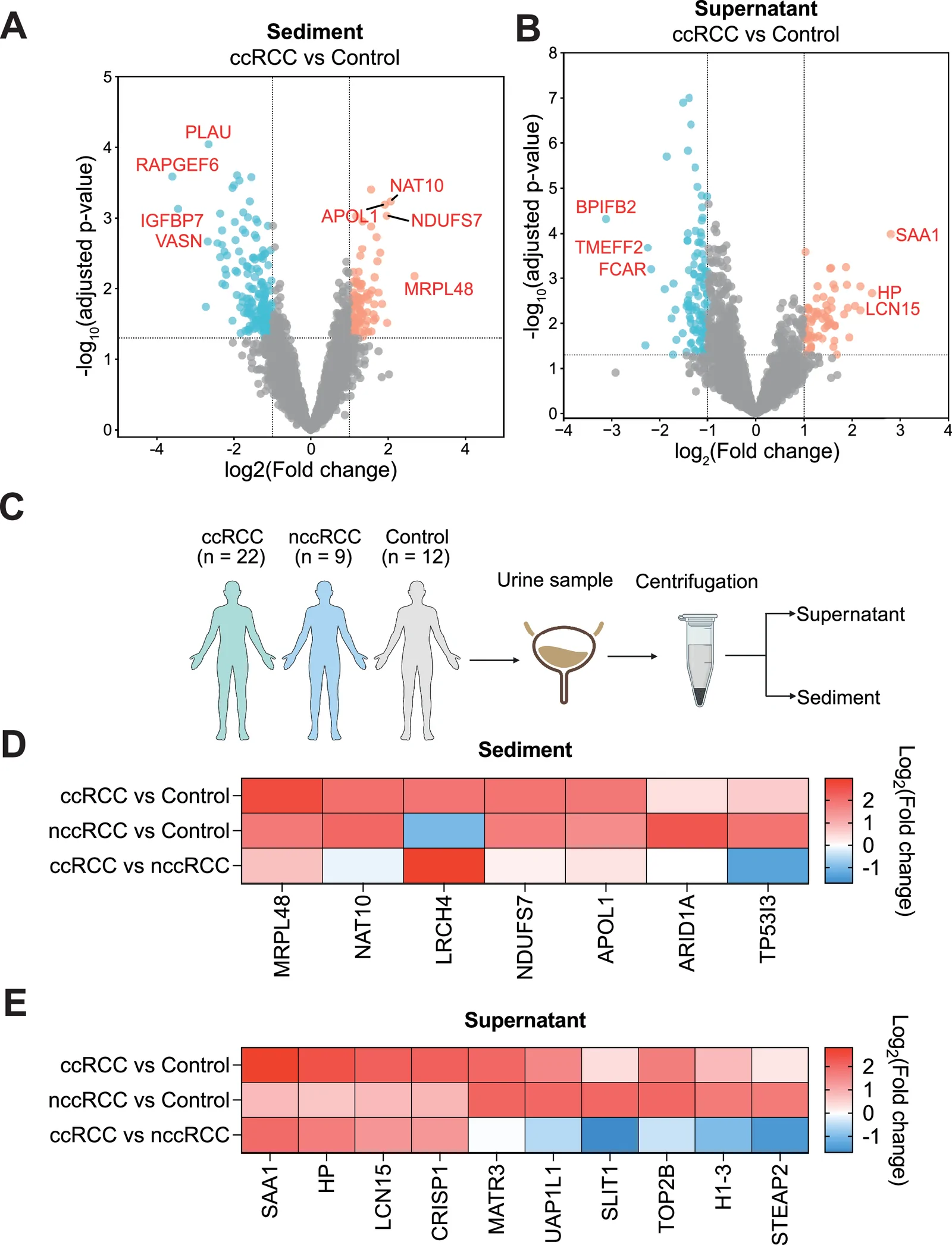
Identification of protein biomarkers for ccRCC diagnosis. (**A**) Volcano plot showing upregulated (red) and downregulated (blue) proteins in ccRCC patients (*n* = 21, the protein amounts were too low in 1 ccRCC sample for proper measurements) compared to controls (*n* = 8, the protein amounts were too low in 4 control samples for proper measurements) in urine sediment. Adjusted *P* values calculated via limma-moderated Benjamini–Hochberg-corrected two-sided *t* test. FC fold change. (**B**) Volcano plot showing upregulated (red) and downregulated (blue) proteins in ccRCC patients (*n* = 22) compared to controls (*n* = 12) in urine supernatant. Adjusted *P* values calculated via limma-moderated Benjamini–Hochberg-corrected two-sided *t* test. FC fold change. (**C**) Schematic of sample acquisition from ccRCC patients, nccRCC patients, and controls for urine supernatant and sediment proteomics. (**D**) Heatmap of the highest upregulated proteins in ccRCC (*n* = 21, the protein amounts were too low in 1 ccRCC sample for proper measurements) vs control (*n* = 8, the protein amounts were too low in 4 controls samples for proper measurements) urine (top row) and nccRCC (*n* = 7, the protein amounts were too low in 2 nccRCC sample for proper measurements) vs control urine (middle row) in urine sediments. A comparison between ccRCC and nccRCC is shown in the bottom row. (**E**) Heatmap of the highest upregulated proteins in ccRCC (*n* = 22) vs control (*n* = 12) urine (top row) and nccRCC (*n* = 9) vs control urine (middle row) in urine supernatants. A comparison between ccRCC and nccRCC is shown in the bottom row.

For these reasons, we focused our efforts on finding more readily assessable biomarkers in our proteomics dataset to potentially be combined with lipidomic signatures for diagnosis. Proteins also provide additional benefits in the clinic. Mass spectrometry-based proteomics is frequently available and established in hospital diagnostic laboratories compared to both lipidomics and metabolomics. Protein-level diagnosis further offers the benefit of being able to develop routine diagnostic solutions, e.g., ELISA-based approaches for cost-effective and routine disease screening. Our bulk proteomics data on both urine sediment and supernatant revealed upregulated proteins with potential as diagnostic biomarkers, e.g., NAT10, APOL1, NDUFS7, and MRPL48 for sediment (Fig. 3A) and SAA1, HP, and LCN15 for the supernatant (Fig. 3B). Mass spectrometry protein data are available in Datasets EV1 and 2.

**Figure EV3:**
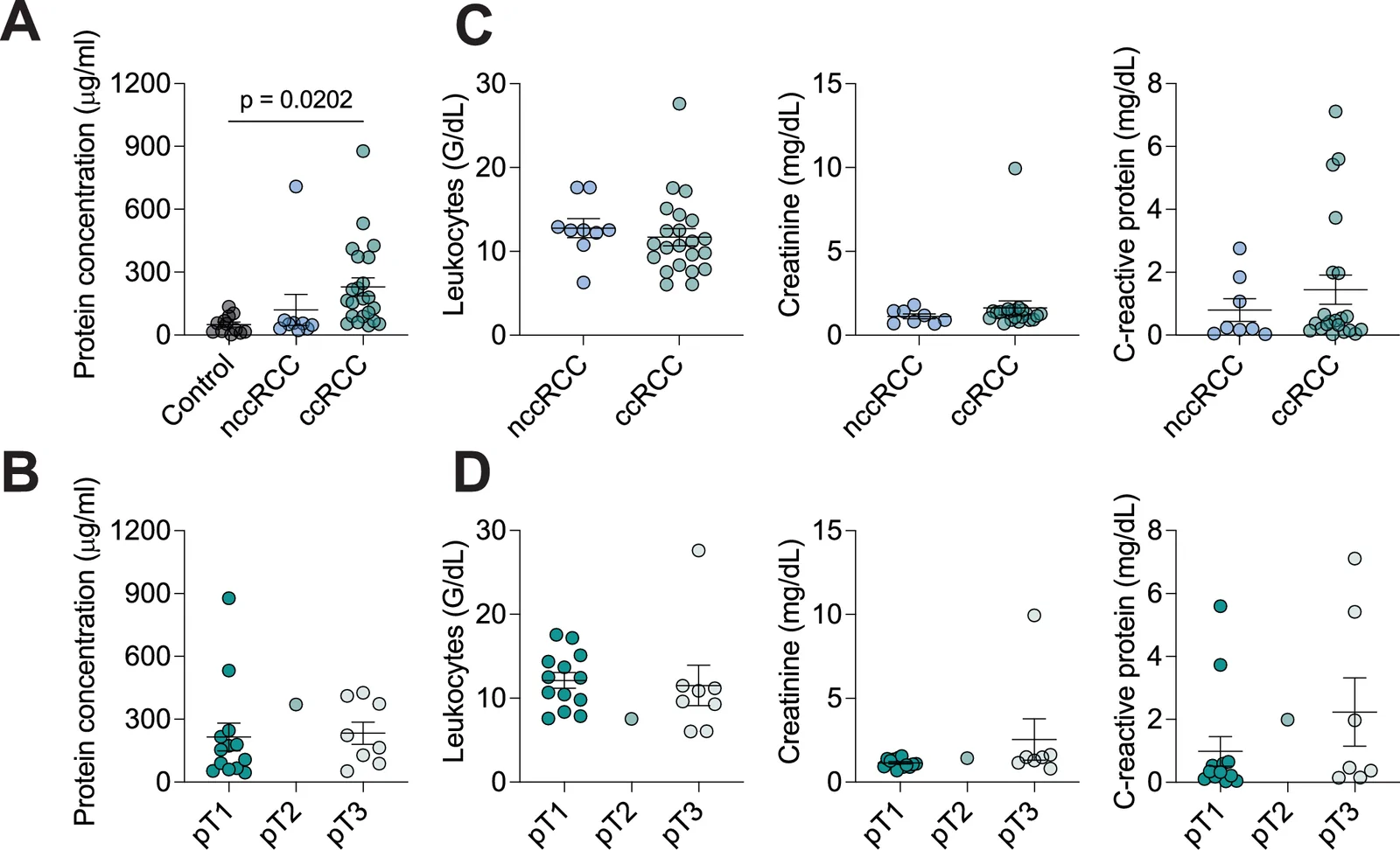
Clinical cohort overview data. (**A**) Protein concentration in urine supernatant from control (*n* = 12), nccRCC (*n* = 9) patients, and ccRCC (*n* = 22) patients determined via Bradford protein assay. Statistical significance assessed through the Kruskal–Wallis test with Dunn’s multiple comparisons test. The mean is shown ± SEM. (**B**) Protein concentration in urine supernatant from ccRCC (*n* = 22) patients divided into pT1 (*n* = 13), pT2 (*n* = 1), and pT3 (*n* = 8) stages determined via Bradford protein assay. pT1 = tumor stage 1. The tumor is a maximum of 7 cm across. pT2 = tumor stage 2. The tumor is larger than 7 cm across. pT3 = tumor stage 3. The tumor has grown into a major renal vein (e.g., vena cava or renal vein) or into neighboring tissues but has not spread past Gerota’s fascia or into the adrenal gland. The mean is shown ± SEM. (**C**) Leukocyte, Creatinine, and C-reactive protein levels in serum, respectively, for nccRCC (*n* = 9) patients and ccRCC (*n* = 22) patients. The mean is shown ±  SEM. (**D**) Leukocyte, Creatinine, and C-reactive protein levels in serum of ccRCC (*n* = 22) patients, stratified at pT1 (*n* = 13), pT2 (*n* = 1), and pT3 (*n* = 8) stages. pT1 = tumor stage 1. The tumor is a maximum of 7 cm across. pT2 = tumor stage 2. The tumor is larger than 7 cm across. pT3 = tumor stage 3. The tumor has grown into a major renal vein (e.g., vena cava or renal vein) or into neighboring tissues but has not spread past Gerota’s fascia or into the adrenal gland. The mean is shown ± SEM.

For any potential diagnostic biomarker, in addition to being able to distinguish between ccRCC and controls, we also wanted to test if such markers can distinguish between ccRCC and non-clear cell renal cell carcinomas (nccRCC), such as pRCC and chRCC. For this purpose, we extended our cohort to also include nine nccRCC patients, from which eight patients had pRCC and one patient had chRCC (Fig. 3C; Table EV1). ccRCC patients had the highest concentration of protein in the urine supernatant (Fig. EV3A). The overall amount of protein found in the urine of ccRCC patients was independent of the tumor stage (Fig. EV3B). Furthermore, for leukocytes and inflammatory damage markers (Creatinine and C-reactive protein) in the serum, no differences were found between ccRCC and nccRCC patients, nor between different stages of ccRCC (Fig. EV3C,D).

**Figure EV4:**
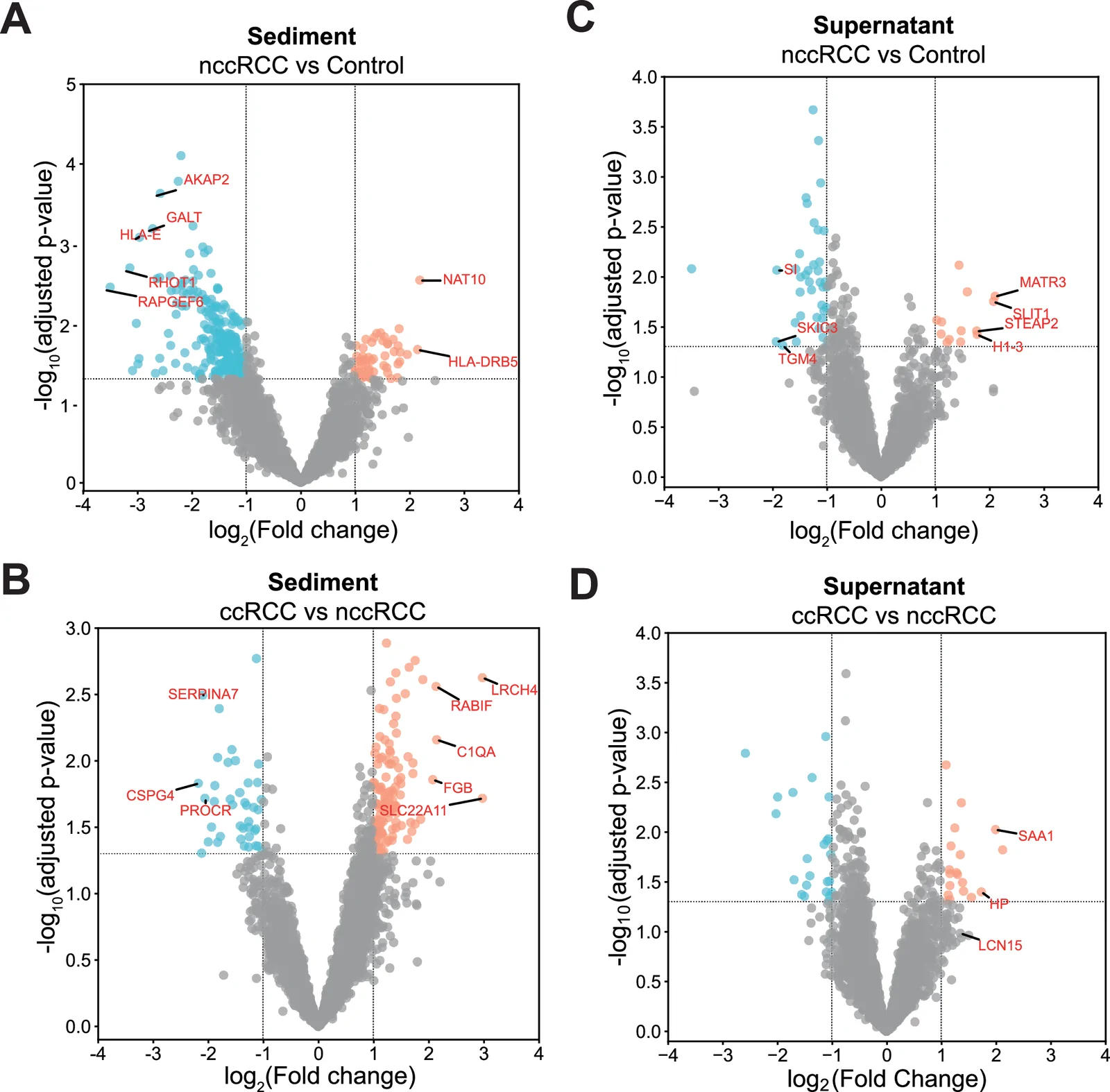
Distinct urinary protein landscapes in ccRCC and nccRCC patients. (**A**) Volcano plot showing upregulated (red) and downregulated (blue) proteins in nccRCC (*n* = 7) patients compared to controls (*n* = 8) in urine sediment. Adjusted *P* values calculated via limma-moderated Benjamini–Hochberg-corrected two-sided *t* test. (**B**) Volcano plot showing upregulated (red) and downregulated (blue) proteins in nccRCC (*n* = 7) patients compared to ccRCC (*n* = 21) patients in urine sediment. Adjusted *P* values calculated via limma-moderated Benjamini–Hochberg-corrected two-sided *t* test. (**C**) Volcano plot showing upregulated (red) and downregulated (blue) proteins in nccRCC (*n* = 9) patients compared to controls (*n* = 12) in urine supernatant. Adjusted *P* values calculated via limma-moderated Benjamini–Hochberg-corrected two-sided *t* test. FC fold change. (**D**) Volcano plot showing upregulated (red) and downregulated (blue) proteins in ccRCC (*n* = 22) patients compared to nccRCC (*n* = 9) patients in urine supernatant. Adjusted *P* values calculated via limma-moderated Benjamini–Hochberg-corrected two-sided *t* test. FC fold change.

When comparing nccRCC urine sediments to controls, NAT10 and MRPL48 were the highest upregulated proteins, just as for ccRCC urine sediments compared to controls (Figs. 3D and EV4A). Overall, the upregulated protein profiles were similar for both ccRCC and nccRCC, with LRCH4 and TP53I3 being the only proteins with the potential of stratifying between ccRCC and nccRCC patient samples (Figs. 3D and EV4B). For supernatants, however, different proteins were upregulated between cancer and control for ccRCC and nccRCC, except for MATR3 (Figs. 3E and EV4C,D). Urine supernatant proteins were chosen as the preferred source of diagnostic biomarkers due to some sediment samples not having sufficient protein quantities for MS-based detection (Dataset EV2): 4/12 controls, 1/22 ccRCC and 2/9 nccRCC sediment samples did not have sufficient protein amounts for MS. Serum Amyloid A1 (SAA1), Haptoglobin (HP), and Lipocalin 15 (LCN15) were selected as the putative supernatant urinary biomarkers for ccRCC for follow up studies based on the following criteria: (i) having a log_2_(Fold change) >2 in ccRCC compared to controls, (ii) upregulated in the ccRCC compared to controls, but not in nccRCC compared to controls, eliminating MATR3, (iii) having a log_2_(Fold change) >1 in ccRCC compared to nccRCC and (iv) not being male or female specific, e.g., CRISP1 is male specific.

### Parallel reaction monitoring mass spectrometry rapidly detects SAA1, HP, and LCN15 in ccRCC urine

Conventional bulk proteomics allows discovery at the full proteome level but is not suitable for rapid diagnostics and population screenings in a clinical setting due to long instrument run times, costs, and computationally intensive data analysis. Therefore, we utilized parallel reaction monitoring mass spectrometry (PRM-MS) to develop a tractable diagnostic modality for SAA1, HP, and LCN15. PRM-MS is an ion monitoring technique allowing parallel high-resolution detection of peptides of interest, drastically reducing the run time per sample and increasing specificity compared to bulk proteomics and other ion monitoring techniques (Peterson et al, 2012).

**Figure 4:**
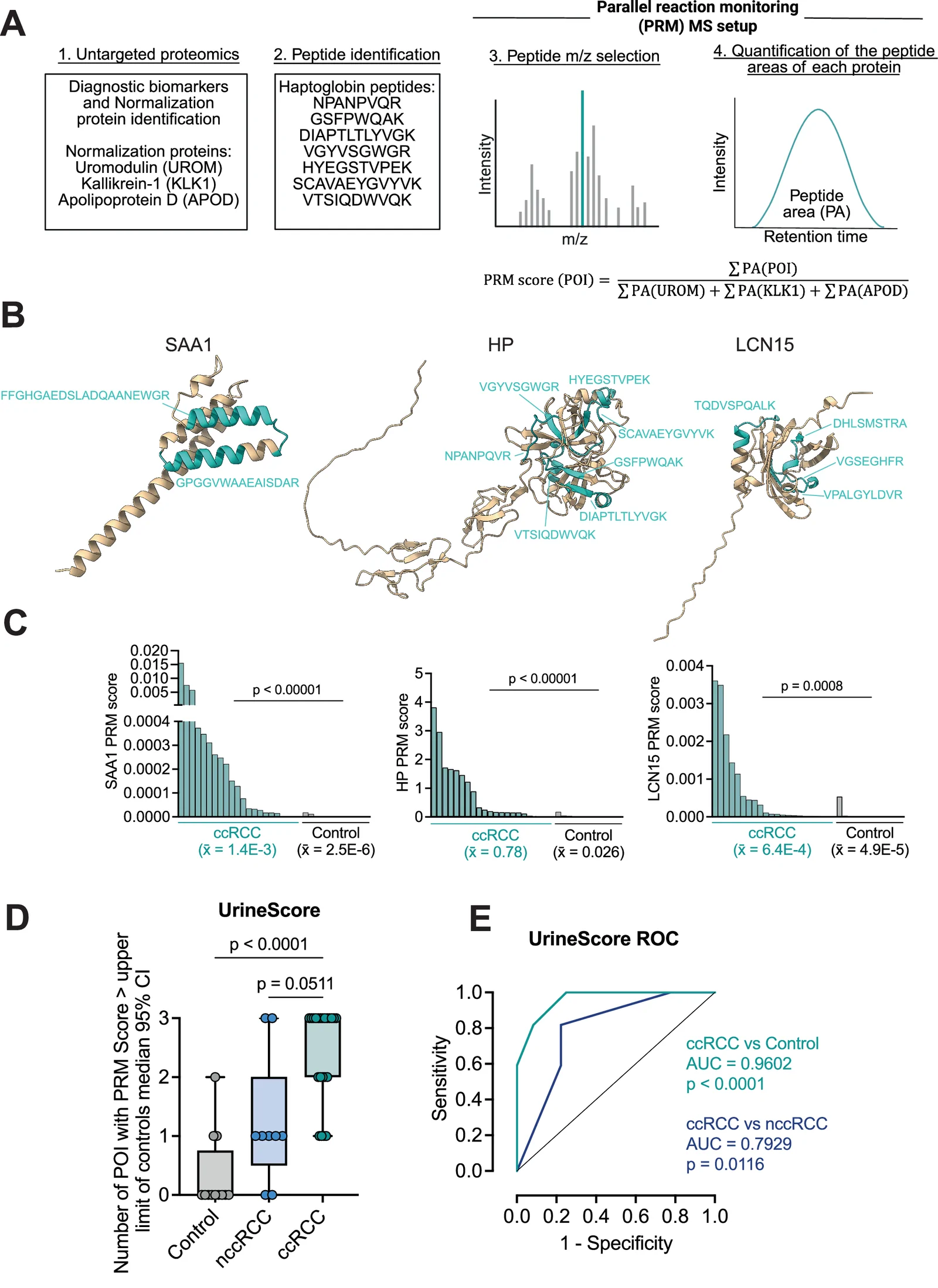
Parallel reaction monitoring mass spectrometry accurately diagnoses ccRCC. (**A**) Schematic of the PRM-MS method used to quantify the HP, SAA1, and LCN15 levels in urine samples. Levels are determined based on normalization to three normalization proteins (UMOD, KLK1, and APOD). The peptide areas for each detected peptide from a specified protein were quantified. Detected peptides from Haptoglobin are shown as an example. PA peptide area, POI protein of interest (SAA1, HP, or LCN15). (**B**) AlphaFold2 structures of HP, SAA1, and LCN15 with the individual peptides detected per protein in PRM-MS highlighted in teal. (**C**) Waterfall plot of PRM score for Haptoglobin, SAA1, and LCN15, respectively, compared between ccRCC (*n* = 22) and control (*n* = 12) cohorts. Statistical significance assessed through a two-sided Mann–Whitney *U* test. x̄ = sample mean. (**D**) Boxplot of UrineScore for controls (*n* = 12), nccRCC (*n* = 9), and ccRCC (*n* = 22) patients. Per protein of interest (POI) per patient or control, a value of 1 is derived if the PRM score is higher than the upper limit of the 95% CI for the control. Statistical significance assessed through the Kruskal–Wallis test with Dunn’s multiple comparisons test. The line in the middle of the box denotes the mean of the data, whereas the lower and upper boundaries of the box represent the 25th (Q1) and 75th (Q3) percentiles, respectively. (**E**) Receiver operating characteristic curve to assess the performance of the UrineScore in differentiating between ccRCC (*n* = 22) and control (*n* = 12) samples, and between ccRCC and nccRCC (*n* = 9) samples. Performance quantified through the area under the receiver operating characteristic curve (AUROC) analysis. Source data are available online for this figure.

**Figure EV5:**
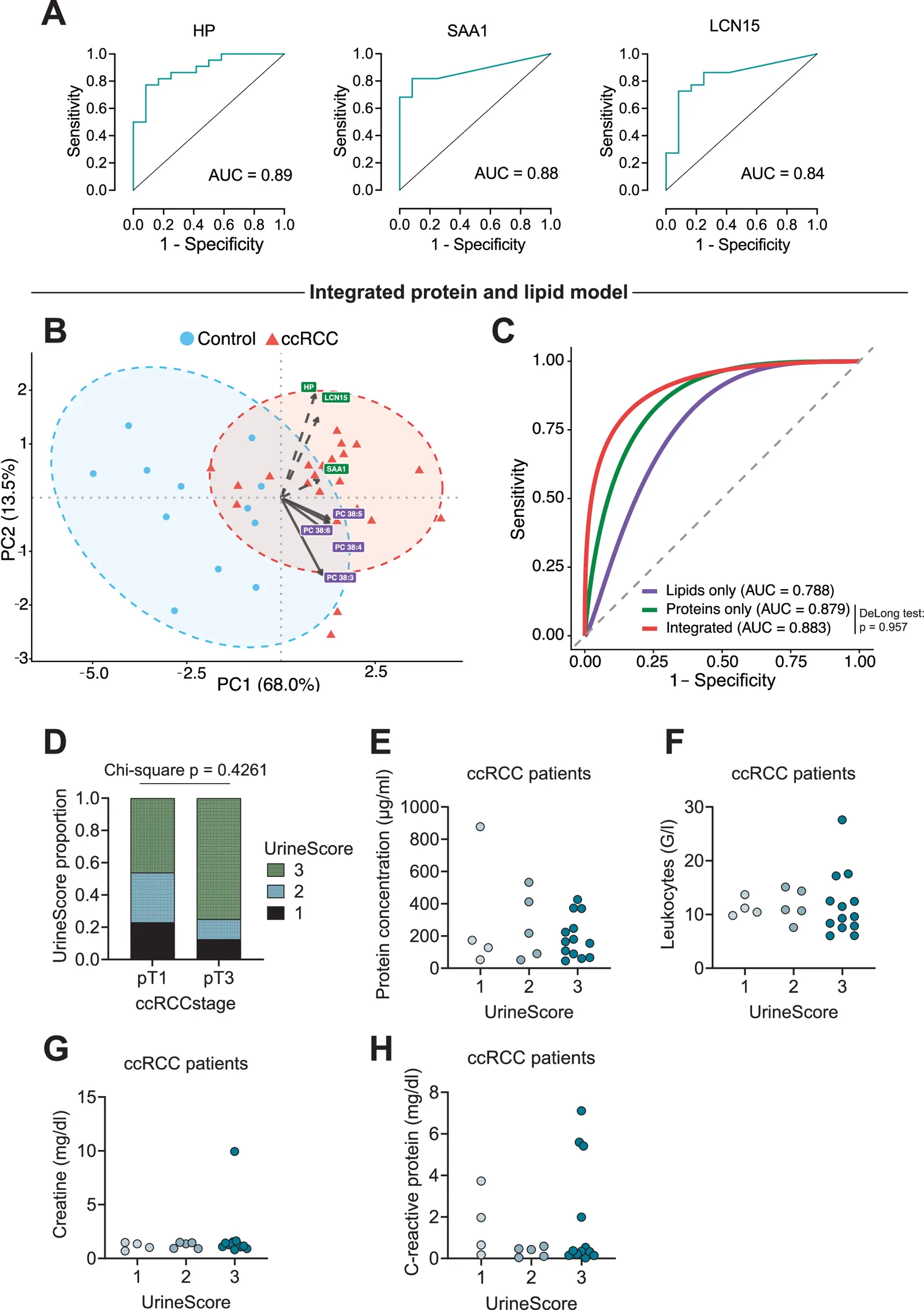
UrineScore components are detectable with PRM-MS in the urine of ccRCC patients and do not correlate with clinical parameters. (**A**) Receiver operating characteristic curve to assess the performance of the PRM score of Haptoglobin, SAA1, and LCN15, respectively, in differentiating between control (*n* = 12) and ccRCC (*n* = 22) samples. Performance quantified through the area under the receiver operating characteristic curve (AUC) analysis. (**B**) Principal component analysis (PCA) of the integrated feature matrix comprising the three UrineScore protein PRM scores (HP, SAA1, LCN15) and the four best-performing urinary phosphatidylcholine species (PC 38:3, PC 38:4, PC 38:5, PC 38:6) in the discovery cohort (*N* = 34; 22 ccRCC, 12 controls). All features were log-transformed prior to analysis, and the feature matrix was mean-centered and scaled to unit variance. PC1 and PC2 explain 68.0% and 13.5% of the total variance, respectively. Data points represent individual samples; ellipses indicate 95% confidence regions. Arrows indicate feature loadings; solid arrows denote lipid species; dashed arrows denote protein markers. (**C**) Receiver operating characteristic (ROC) curves comparing discriminatory performance of three logistic regression models evaluated by leave-one-out cross-validation (LOOCV) in the discovery cohort: lipid species only (PC 38:3, PC 38:4, PC 38:5, PC 38:6; AUC = 0.788), UrineScore proteins only (HP, SAA1, LCN15; AUC = 0.879), and an integrated model incorporating all seven features (AUC = 0.883). Statistical comparison of the integrated and protein-only models was performed using the DeLong test (*P* = 0.957). ROC curves are smoothed using a binormal model for visualization; reported AUC values reflect empirical estimates. All features were log-transformed prior to modeling using the same offsets described in the “Methods”. (**D**) Stacked bar plots showing the distribution of the UrineScore in stage 1 (pT1, *n* = 13) and stage 3 (pT3, *n* = 8) tumors. Stage 2 tumors are excluded from this analysis due to there only being *n* = 1 tumor in this group. Statistical significance assessed through the chi-square test. (**E**) Scatter plot showing the distribution of urinary protein concentrations (measured using BCA) for ccRCC samples scoring 1 (*n* = 4), 2 (*n* = 5), or 3 (*n* = 13) in the UrineScore. Statistical significance assessed through one-way ANOVA. (**F**) Scatter plot showing the distribution of circulating leukocytes for ccRCC samples scoring 1 (*n* = 4), 2 (*n* = 5), or 3 (*n* = 12, leukocytes were not assessed for one UrineScore = 3 sample, in the UrineScore. Statistical significance assessed through one-way ANOVA. (**G**) Scatter plot showing the distribution of serum creatinine levels for ccRCC samples scoring 1 (*n* = 4), 2 (*n* = 5), or 3 (*n* = 12, creatinine was not assessed for one UrineScore = 3 sample) in the UrineScore. Statistical significance assessed through one-way ANOVA. (**H**) Scatter plot showing the distribution of serum C-reactive protein levels for ccRCC samples scoring 1 (*n* = 4), 2 (*n* = 5), or 3 (*n* = 12, C-reactive protein was not assessed for one UrineScore = 3 sample) in the UrineScore. Statistical significance assessed through one-way ANOVA.

In our PRM-MS approach, we included all peptides for SAA1, HP, and LCN15 detected by our bulk proteomics approach with clearly defined elution patterns (Table EV2). For standardization, we included peptides from three normalization proteins, namely Uromodulin (UMOD), Kallikrein-1 (KLK1), and Apolipoprotein D (APOD). A normalization protein was defined as a protein that was found in every sample in all three groups (Control, nccRCC, and ccRCC) and with low variability in expression levels across all three experimental groups per protein. The peptide area was calculated for every selected peptide and the PRM score for each diagnostic protein was determined by dividing the sum of all peptide areas from individual proteins of interest (SAA1, HP, LCN15) with the sum of all peptides from all three normalization proteins (Fig. 4A). The detected peptides for the three diagnostic proteins used in the PRM-MS analysis are structurally shown in Fig. 4B. The PRM scores for SAA1, HP, and LCN15 were all significantly higher in ccRCC patients compared to controls (Fig. 4C). The classification performance of each PRM score was calculated by the AUROC method and was evaluated to be 0.88, 0.89, and 0.84 for SAA1, HP, and LCN15, respectively (Fig. EV5A). Furthermore, we attempted to include an integrated model of both lipidomics and proteomics to increase the diagnostic power in detecting ccRCC in urine. Combining the three protein PRM values with the four lipid species which had the best performance in differentiating between ccRCC and controls (Fig. 2D; PC 38:3, PC 38:4, PC 38:5, and PC 38:6) resulted in a clear separation of controls and ccRCC cases (Fig. EV5B). To quantify the performance in distinction for proteins alone, lipids alone, and integrated, we created a Leave-one-out cross-validation (LOOCV) logistic regression model yielding an AUC of 0.879 for proteins alone and 0.788 for lipids alone, and 0.883 for the integrated model (Fig. EV5C). While the integrated model did not reach statistical superiority over the protein-only model (DeLong test *P* = 0.957), likely reflecting limited statistical power at *n* = 34, the PCA demonstrates that lipid and protein features capture partially complementary variance (PC1 = 68%). Therefore, the lipidomics approach was not included for further diagnostic method development. In our smaller cohort, we deliberately maintained separate workflows for lipidomics and proteomics to ensure maximal sensitivity and specificity for each analyte class, and the current discovery cohort proteins alone were used to construct a dichotomous scoring model.

Hence, all three individual protein PRM scores were combined into a cumulative UrineScore, detailed in the Methods section. In brief, the 95% confidence interval (CI) of the median was calculated for all PRM parameters (SAA1, HP, and LCN15 PRM scores) for the control samples. Subsequently, each sample from the control, nccRCC, and ccRCC groups was attributed a score of 1 per protein (SAA1, HP, and LCN15) if the corresponding PRM score is higher than the control 95% CI. If the corresponding PRM score is lower, a value of 0 is attributed, meaning that the UrineScore is an integer between 0 and 3 per sample. The resulting UrineScores were the highest for the ccRCC group (Fig. 4D) and had a high performance in distinguishing between control and ccRCC samples (AUROC = 0.96, 95% CI: 0.873–1.000) and also performed well between nccRCC and ccRCC samples (AUROC = 0.78, 95% CI: 0.560–0.964) (Fig. 4E). These data indicate that PRM-MS indeed allows for rapid and sensitive diagnostics compared to bulk proteomics in the context of ccRCC, and that SAA1, HP and LCN15 can be used to diagnose ccRCC in our discovery cohort.

Overall, in our discovery cohort, there was no significant difference in the distribution of the UrineScore in pT1 and pT3 patients (Fig. EV5D), indicating that the UrineScore is stable in ccRCC patients across different stages. Furthermore, the different UrineScores did not correlate with various clinical parameters, such as overall urine protein concentration (Fig. EV5E), immune activation measured through leukocytes (Fig. EV5F), kidney function measured through creatinine levels in the blood (Fig. EV5G), nor overall inflammation measured through C-reactive protein in blood (Fig. EV5H).

### The UrineScore validates in other cohorts

**Figure 5:**
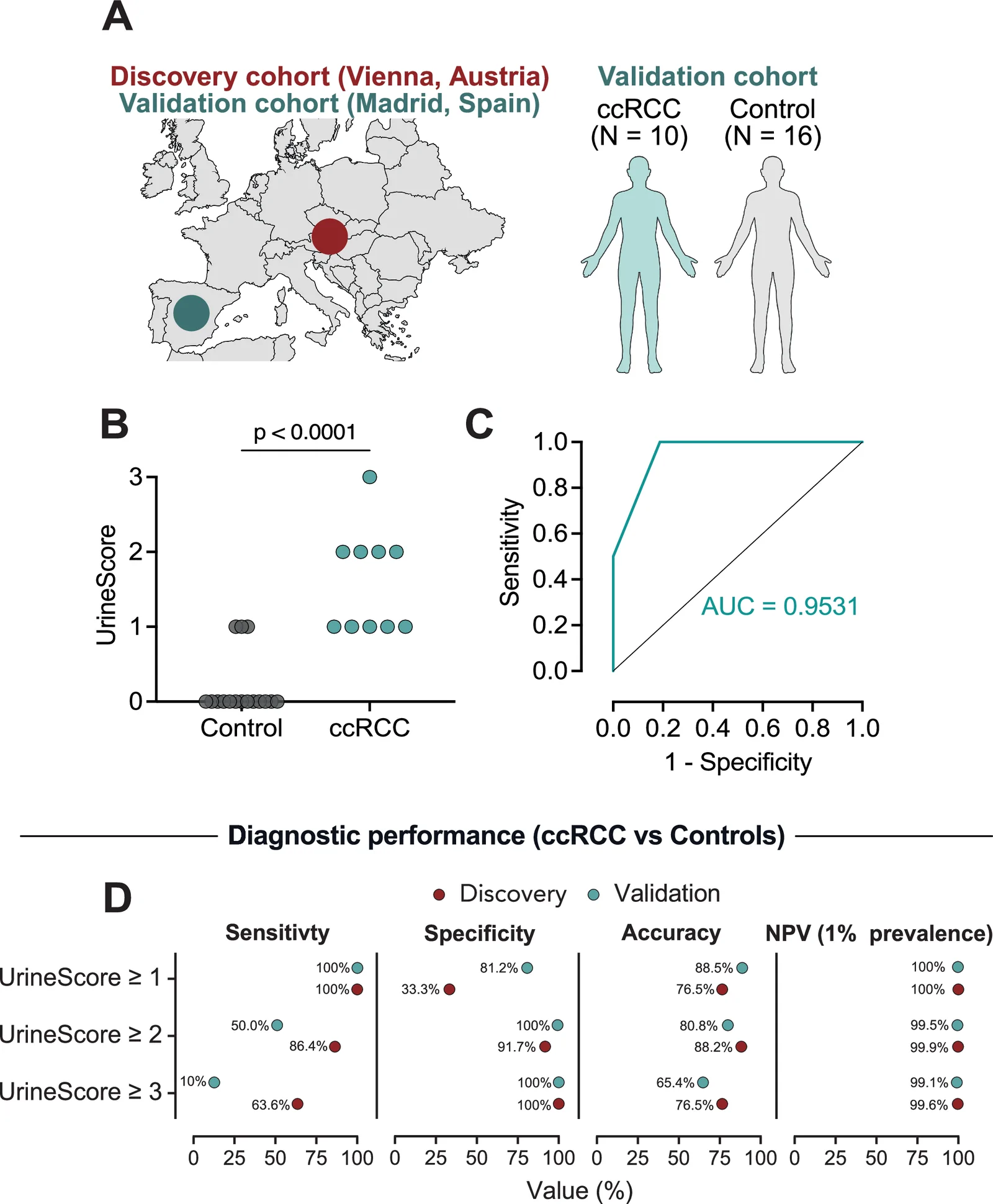
The UrineScore validates in clinical and computational cohorts. (**A**) Schematic of ccRCC patients (*n* = 10) and controls (*n* = 16) from which samples were acquired in a validation cohort from the biobank of Ramón y Cajal University Hospital, Madrid. (**B**) Scatter plot of UrineScore for controls (*n* = 16) and ccRCC (*n* = 10) patients from the validation cohort. Normality assessed through Shapiro–Wilk normality test followed by statistical significance assessed through a two-sided Mann–Whitney *U* test. (**C**) Receiver operating characteristic curve to assess the performance of the UrineScore in differentiating between ccRCC (*n* = 10) and control (*n* = 16) samples in the validation cohort. Performance quantified through the area under the receiver operating characteristic curve (AUROC) analysis. (**D**) Performance evaluation of the UrineScore at three different thresholds (UrineScore ≥1, ≥2, ≥3) in the discovery and validation cohort. Performance is evaluated through the sensitivity, specificity, accuracy, and NPV at 1% prevalence. NPV negative predictive value. Further performance metric data is available in Table EV4. Source data are available online for this figure.

To validate the UrineScore, we analyzed urine samples from ccRCC (*n* = 10) patients and controls (*n* = 16) from the biobank at Ramón y Cajal University Hospital in Madrid (Fig. 5A; Table EV3). All samples were subjected to the UrineScore PRM pipeline, and the scoring baseline based on the control was re-calculated for the validation cohort. As in the discovery cohort, the UrineScore was significantly higher in the ccRCC group (Fig. 5B) and performed similarly well in distinguishing between ccRCC patients and controls (AUROC = 0.95, 95% CI: 0.887–1.000) (Fig. 5C).

To more rigorously evaluate the performance of the UrineScore, we calculated the specificity, sensitivity, accuracy, positive predictive value (PPV), and negative predictive value (NPV) at all three putative diagnostic thresholds of UrineScore ≥ 1, ≥2, and ≥3 (Fig. 5D; Table EV4). At the most sensitive threshold (UrineScore ≥1), the assay achieved 100% sensitivity and 100% NPV in the discovery cohort, with 100% sensitivity and 100% NPV in the validation cohort. At a UrineScore threshold of ≥2, the assay achieved a sensitivity of 86.4% and specificity of 91.7% in the discovery cohort, with specificity maintained at 100% in the independent validation cohort. Critically, the negative predictive value exceeded 99.0% at all thresholds across both cohorts when calculated at a realistic population screening prevalence of 1%, supporting the UrineScore as a rule-out tool for ccRCC in at-risk populations.

**Figure 6:**
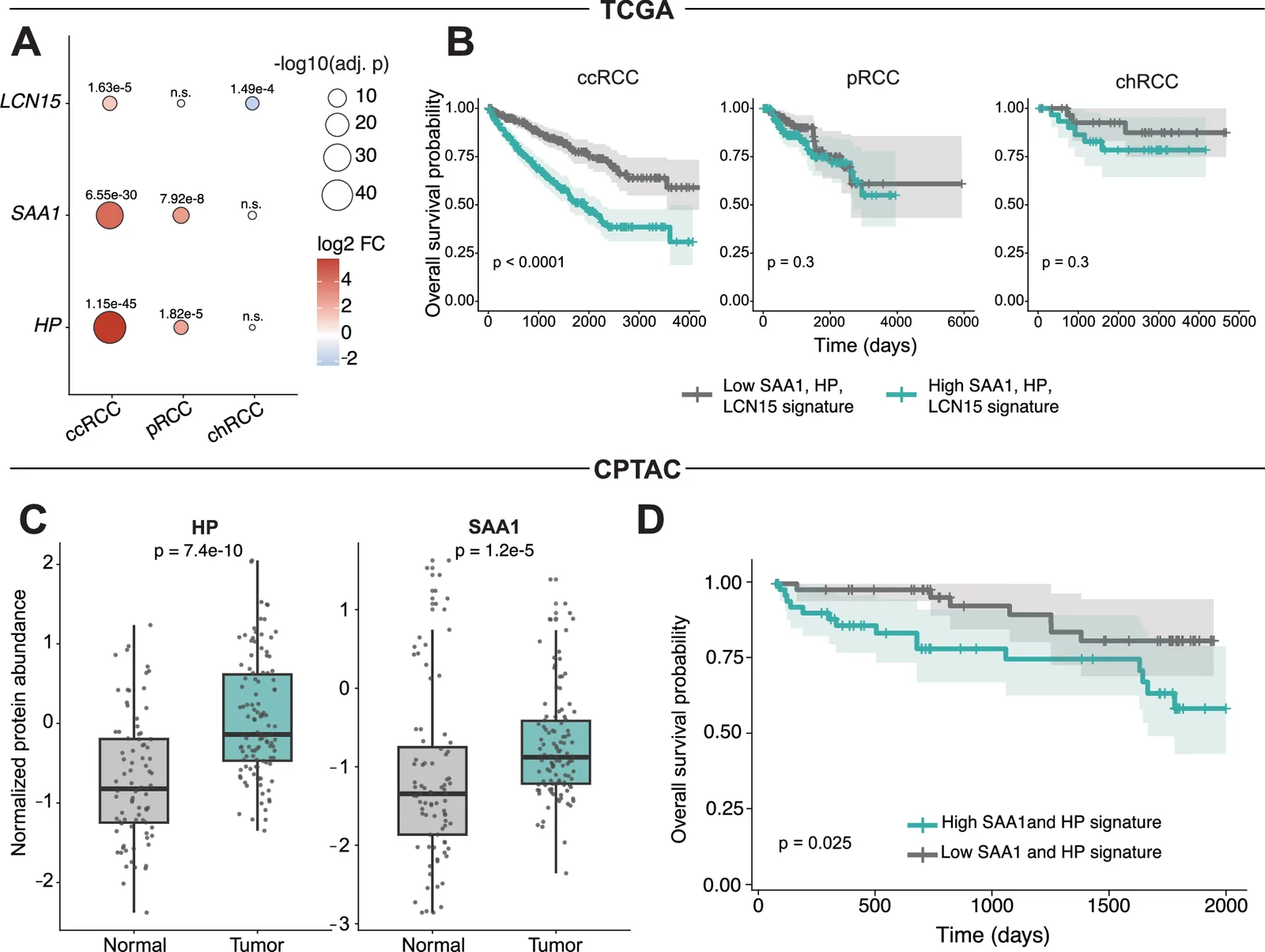
HP, SAA1, and LCN15 are upregulated in ccRCC tumors and predict survival. (**A**) Bubble plot showing the expression upregulation of HP, SAA1, and LCN15 in tumor samples compared to normal samples (ccRCC: *n* = 72, pRCC: *n* = 32, chRCC: *n* = 25) in ccRCC (*n* = 541), pRCC (*n* = 290), and chRCC (*n* = 66) TCGA cohorts. Statistical significance assessed through Wald test with Benjamini–Hochberg correction for multiple testing on the entire transcriptome per cancer type. (**B**). Kaplan–Meier overall survival curves based on a *HP*, *SAA1*, and *LCN15* combined transcriptome signature for the ccRCC (*n* = 537), pRCC (*n* = 287), and chRCC (*n* = 65) TCGA cohorts. Lower *n* numbers in this panel compared to (**A**) is derived from the survival analysis only using primary tumor samples with overall survival data, which was not available for all samples. The cohorts were split into high-signature and low-signature groups along the median. Statistical significance was assessed through Log-rank tests. (**C**) Boxplot showing the expression upregulation of HP and SAA1 in tumor samples (*n* = 110) compared to normal tissue samples (*n* = 84) in the ccRCC CPTAC cohort. Statistical significance assessed through the Wald test with Benjamini–Hochberg correction for multiple testing on the entire proteomics dataset. The line in the middle of the box denotes the mean of the data, whereas the lower and upper boundaries of the box represent the 25th (Q1) and 75th (Q3) percentiles, respectively. (**D**) Kaplan–Meier overall survival curves based on HP and SAA1 combined proteome signature for the ccRCC CPTAC (*n* = 104) cohort. Six samples were lacking overall survival data, leading to a lower *n* number compared to (**C**). The cohorts were split into high-signature and low-signature groups along the median. Statistical significance was assessed through Log-rank tests.

Finally, we validated our UrineScore biomarkers in TCGA datasets containing ccRCC, pRCC and chRCC samples; *HP*, *SAA1*, and *LCN15* mRNA showed a strong and specific upregulation in KIRC tumors compared to normal tissue, unlike KIRP where the upregulation was markedly lower and KICH which did not show upregulation of these markers (Fig. 6A). Furthermore, a combined signature of high *HP*, *SAA1*, and *LCN15* expression showed prognostic potential in ccRCC where high expression was associated with worse overall survival (Fig. 6B). Interestingly, the UrineScore had no prognostic effect in pRCC nor chRCC. Lastly, in a proteomics-based ccRCC cohort from the Clinical Proteomic Tumor Analysis Consortium (CPTAC), both HP and SAA1 were upregulated in ccRCC tumors compared to healthy controls (Fig. 6C), and a high combined signature of the two was negatively correlated to overall survival (Fig. 6D). LCN15 was not detected at the protein level in the CPTAC ccRCC cohort. The convergent identification of the UrineScore in the discovery and validation cohorts highlights the potential of HP, SAA1, and LCN15 as biomarkers for ccRCC.

## Discussion

In 2020, ~430,000 new cases of renal cell carcinomas were diagnosed worldwide (Bukavina et al, 2022). Out of all of these cases, it is estimated that 80% of the renal cell carcinomas are ccRCC. With a growing incidence rate, there is an unmet need for cost-effective, rapid, and sensitive diagnostic tools. Despite the high clinical interest, there are currently no clinically validated biomarkers for clinical screening, and common diagnostic procedures such as computed tomography are not suitable for large-scale monitoring (Diana et al, 2023; Usher-Smith et al, 2022).

Utilizing urinary proteomics, metabolomics, and lipidomics, here we uncovered aberrant metabolism throughout the urogenital tract of ccRCC patients. Our data suggests increased mitochondrial respiration and lipid metabolism. ccRCC is known to have a lipid-rich cytoplasm (Du et al, 2017). We also discovered increased lipid transport and a reduction in carnitine synthesis. Although our data comes from the urine and not directly from the tumor, one potential explanation for the increased lipid accumulation in ccRCC tumors could be a reduction of carnitine, which is essential for the breakdown of free fatty acids and potentially lipid transport to the tumor microenvironment (Marques et al, 2018). Among the individual lipid species detected, we noticed an increase in certain PE and PC species, indicative of increased tissue damage and membrane shedding into the excreted compartment. We further found increased levels of CoQ10, corroborating our observations of increased mitochondrial respiration. Generally, ccRCC tend to be independent of oxidative phosphorylation as a source of energy (Zhang et al, 2021), suggesting that the mitochondrial respiration phenotypes we identified in urine sediment are likely coming from shed epithelium and/or surrounding tissue damage from the tumor.

Proteomics on the urine supernatants proved to be the most reliable and feasible for diagnostic biomarker discovery. Metabolomics only revealed significantly downregulated biomarkers in ccRCC compared to controls, making reliable assessment and case distinction difficult due to the overall low levels of analytes available in the urine. Lipidomics, on the other hand, performed well even though the overall number of identified lipid species was low but showing clear upregulation in ccRCC urine compared to controls. The overall performance, however, was lower than that of proteomics. Furthermore, in an integrated model of both lipids and proteins, the model performance was not significantly increased over proteomics alone. Therefore, we proceeded with only proteomics for further method development. Integrating lipidomics and proteomics into one unified PRM-MS workflow is challenging due to, e.g., conflicts in ionization chemistry, incompatibilities in dynamic range, and differences in sample preparation. For future larger cohorts, PRM-MS-based lipidomics should still be investigated as a complementary approach to proteomics, either as an integrated score or as a stand-alone modality.

Bulk proteomics uncovered three putative diagnostic biomarkers (SAA1, HP, and LCN15) in our clinical discovery cohort. Furthermore, through the establishment of PRM-MS for the rapid and sensitive detection of these three proteins of interest, we could derive PRM scores for SAA1, HP, and LCN15 with high diagnostic power in the discovery cohort. When these PRM scores were combined into an UrineScore, we calculated an overall performance in an AUROC analysis of 96% in distinguishing between healthy controls and ccRCC. The UrineScore also performed well in distinguishing between ccRCC and nccRCC patients, though this needs to be confirmed in larger patient cohorts. Lastly, we were able to extend our analysis to an independent validation cohort, in which the UrineScore had comparable performance to the discovery cohort. However, the sensitivity of the UrineScore at the ≥2 operating point was lower in the validation cohort (50.0%) than in the discovery cohort (86.4%), which we attribute to the limited number of ccRCC cases in the validation cohort (*n* = 10) rather than a systematic difference between cohorts, as specificity was well-maintained. The positive predictive value of the UrineScore at realistic population screening prevalence (1%) is inherently low, as expected for a screening-oriented test applied to a low-prevalence condition. The confidence intervals around sensitivity and specificity estimates, particularly in the validation cohort (*n* = 10 ccRCC, *n* = 16 controls), are wide and preclude definitive conclusions about absolute diagnostic performance at this stage. Therefore, larger, clinically representative cohorts, including patients with benign renal masses and other urological conditions, will be essential to establish stable performance estimates before any clinical translation. Lastly, the current UrineScore is easily expanded to include additional proteins measured by PRM-MS if additional cohorts are measured using bulk proteomics in which extra putative diagnostic biomarkers are uncovered. Our combined results emphasize the relevance of urine-based diagnostic in urogenital malignancies, while at the same time highlighting the need for further biomarker panel discovery and development.

A key limitation of the present study is the use of healthy volunteer donors as control subjects in both the discovery and validation cohorts. While this design is appropriate for an initial proof-of-concept study aimed at establishing whether a signal exists between ccRCC patients and a healthy baseline, it does not reflect the clinical reality of the urological setting in which such a test would ultimately be deployed. In practice, patients presenting with suspected renal masses or hematuria frequently carry comorbidities, including urinary tract infections, nephrolithiasis, chronic kidney disease, or benign renal tumors such as oncocytomas and angiomyolipomas, all of which could plausibly influence urinary protein levels. SAA1 and HP, in particular, as hepatic acute-phase reactants, may be elevated in any systemic inflammatory condition. Thus, the cellular source of SAA1, HP, and LCN15, and how these three proteins are being filtered/shed into the urine, remains to be answered. All three proteins are secreted proteins, meaning that they could be directly produced at high levels in the tumors and subsequently secreted into the urine. However, according to the Human Protein Atlas, SAA1 is produced as an acute-phase protein in the liver, HP is produced in the liver to counteract free hemoglobin, and LCN15 is produced in the gastrointestinal tract (Uhlén et al, 2015). It is possible that circulating serum proteins are found at higher levels in the urine of ccRCC patients due to tumor-induced tissue damage of the filtration unit of the kidney, effectively increasing the leakiness of serum proteins into the urine that is found in ccRCC-associated hematuria. However, our TCGA and CPTAC analyses presented here provide biological support for tumor-specific upregulation of all three markers at the tissue level, and the ccRCC-favored expression pattern across RCC subtypes provides some confidence in their specificity, but this does not substitute for prospective evaluation in a clinically representative control population. Future validation studies should prioritize the inclusion of patients with benign renal masses, inflammatory renal conditions, and other urological malignancies as comparators, and should systematically collect inflammatory markers, renal function parameters, and urinalysis data to allow confounding to be formally assessed and, where necessary, corrected for in the scoring model.

The urine proteome of ccRCC patients has been studied before. For example, label-free quantitative proteomics on urine from ccRCC patients identified HP as a urinary marker of ccRCC, confirming HP as a UrineScore component (Sandim et al, 2016). Notably, UMOD was identified as a differentially secreted protein in ccRCC urine. In our study, UMOD is used as a normalizer in our PRM-MS assay together with KLK1 and APOD. UMOD is the most abundant protein in urine under physiological conditions, present at concentrations orders of magnitude higher than SAA1 or LCN15. Its identification as differentially secreted in ccRCC urine in untargeted discovery proteomics could reflect genuine tumor-associated regulation or, alternatively, inter-individual variation in urinary dilution and/or renal tubular function. We chose UMOD as a normalizer due to its constitutive expression in the loop of Henle, making it a pragmatic and widely used urinary reference protein to control for sample-to-sample variations. Its use as a normalizer rather than a candidate marker in our study is consistent with its established role in urinary proteomics normalization pipelines. The previously reported differential regulation of UMOD in ccRCC urine is nevertheless an interesting finding that merits independent investigation in future studies with dedicated normalization strategies.

Beyond urinary liquid biopsies, both SAA1 and HP proteins have been identified in the serum of ccRCC patients and were proposed as candidate serum biomarkers (Tolson et al, 2004). The convergent identification of these proteins in both serum and urine across independent cohorts, institutions, and analytical platforms spanning two decades provides compelling cross-matrix validation of their biological relevance to ccRCC. Other studies have also identified distinct signatures in ccRCC urine, including N-glycoproteomic markers associated with disease progression (Santorelli et al, 2020), proteomic signatures of renal vein infiltration (Chinello et al, 2019), or prognostic models derived from urine proteomics (Yang et al, 2023; Di Meo et al, 2020). All these studies support the notion that the urine proteome carries diagnostically and prognostically relevant information in ccRCC. However, no study has identified SAA1, HP, and LCN15 as a composite diagnostic signature. Of note, unlike HP and SAA1, LCN15 has historically been poorly characterized. A recent systematic mapping of human lipid transfer proteins formally established LCN15 as a functional lipid-binding protein with a structurally distinctive binding pocket (Titeca et al, 2026), placing it in the same functional lipocalin class as the well-validated urinary biomarker NGAL/LCN2 in acute kidney injury (Mishra et al, 2005). This pattern is consistent with efficient apical secretion rather than intracellular accumulation, a hallmark of the lipocalin family (Flower, 1996), which would result in concentration in the urinary compartment rather than retention in tissue lysates, potentially explaining why we detect LCN15 at the mRNA, but not the protein, level in ccRCC tumors. Taken together, these findings suggest that LCN15 is locally produced in the ccRCC tumor microenvironment, secreted into the tubular lumen, and concentrated in urine, providing a mechanistic rationale for its robust detection by PRM-MS in patient urine samples despite its absence from tissue proteomics databases. This distinguishes LCN15 from HP and SAA1, which are systemically derived acute-phase reactants, and underscores the complementary biological origins of the three UrineScore components.

Beyond urine proteomics, the broader liquid biopsy landscape for ccRCC has expanded considerably in recent years, encompassing cell-free DNA and epigenomic approaches. Notably, it has been demonstrated that cell-free methylated DNA immunoprecipitation sequencing (cfMeDIP-seq) could classify RCC patients across all disease stages with an AUROC of 0.99 in plasma and 0.86 in urine cell-free DNA (Nuzzo et al, 2020). However, cfMeDIP-seq requires deep sequencing infrastructure and substantial bioinformatic pipelines that currently limit its accessibility in routine clinical settings. By contrast, our PRM-MS assay targets three defined protein analytes, offering a workflow that is more readily implementable on existing mass spectrometry infrastructures and more directly scalable to clinical laboratory adoption, especially with the potential to develop ELISA kits. Epigenomic and proteomic approaches are, however, not mutually exclusive and may capture complementary biological signals. Thus, multi-analyte liquid biopsy panels combining methylomic and proteomic, and even lipidomic, readouts could be a productive direction for future diagnostics. Despite the accumulation of promising candidates across these diverse platforms, no urine-based or blood-based biomarker for ccRCC has yet reached clinical implementation, underscoring both the unmet need and the importance of prospective, multicenter validation as the necessary next step for any candidate platform, including the UrineScore.

## Methods

**Table:** Reagents and tools table

Reagent/resource	Reference or source	Identifier or catalog number
**Experimental models**		
N/A		
**Recombinant DNA**		
N/A		
**Antibodies**		
N/A		
**Oligonucleotides and other sequence-based reagents**		
N/A		
**Chemicals, enzymes, and other reagents**		
Acetone	Sigma-Aldrich	179124
Methanol (LC–MS grade)	Thermo Fisher Scientific	047192.K2
Chloroform	Sigma-Aldrich	650498
Urea	Sigma-Aldrich	U5128
Dithiothreitol (DTT)	Roche	10197777001
Indole-3-acetic acid (IAA)	Merck	I3750-5G-A
Lysyl Endopeptidase (LysC), Mass Spectrometry Grade	FUJIFILM Wako	125-05061
Trypsin Gold, Mass Spectrometry Grade	Promega	V5280
Acetonitrile (ACN, LC–MS grade)	Thermo Fisher Scientific	047138.K2
Water (LC–MS grade)	Thermo Fisher Scientific	047146.K2
Trifluoroacetic acid (TFA)	Sigma-Aldrich	T6399
Formic acid (FA)	Sigma-Aldrich	695076
Ammonium bicarbonate	Sigma-Aldrich	A6141
Ammonium acetate	Sigma-Aldrich	238074
Phosphoric acid	Sigma-Aldrich	695017
2-propanol	Sigma-Aldrich	190764
Pierce HeLa Protein Digest Standard	Thermo Fisher Scientific	88329
PE 34:0 (internal standard)	Avanti Polar Lipids	830456P
PS 34:0 (internal standard)	Avanti Polar Lipids	840028P
LPC 17:1 (internal standard)	Avanti Polar Lipids	855677C
SM d35:1 (internal standard)	Avanti Polar Lipids	860585
PC 34:0 (internal standard)	Larodan	37-1700-7
TG 54:0 (internal standard)	Larodan	33-1835-9
**Software**		
Spectronaut 18.5	Biognosys	RRID:SCR_016617
MS2Go (in-house software)	Max Perutz Labs Proteomics Facility	N/A
msReport (Python library)	Max Perutz Labs Proteomics Facility	N/A
Skyline (v22.2.0.351, 64-bit)	MacCoss Lab, University of Washington	RRID:SCR_014080
Compound Discoverer 3.3 SP2	Thermo Fisher Scientific	COMPOUNDDISC3
MS-DIAL	RIKEN	RRID:SCR_016280
Lipid Data Analyzer 2.8.3_2	Graz University of Technology	RRID:SCR_023040
ChimeraX	UCSF	RRID:SCR_015872
AlphaFold Protein Structure Database	EMBL-EBI/DeepMind	https://alphafold.ebi.ac.uk/
GraphPad Prism 10	GraphPad Software	RRID:SCR_002798
RStudio (Posit)	Posit PBC	RRID:SCR_000432
R (v4.5)	R Core Team	RRID:SCR_001905
TCGAbiolinks (v2.4)	Bioconductor	RRID:SCR_017683
DESeq2 (v1.52)	Bioconductor	RRID:SCR_015687
limma	Bioconductor	RRID:SCR_010943
survival (R package)	CRAN	RRID:SCR_021137
survminer (R package)	CRAN	RRID:SCR_021094
ggplot2/ggsurvplot (R package)	CRAN	RRID:SCR_014601
Enrichr	Ma’ayan Laboratory, Icahn School of Medicine	RRID:SCR_001575
MetaboAnalyst	Wishart Research Group	RRID:SCR_015539
**Other**		
PepMap C18 pre-column (5 mm × 300 μm × 5 μm, 100 Å)	Thermo Scientific	164535
Aurora ULTIMATE C18 HPLC column with emitter (25 cm × 75 μm ID, 1.7 μm, CSI)	IonOpticks	AUR3-25075C18-CSI
Column Oven PRSO-V1-BR	Sonation	https://www.sonation.com/en/products/analytics/column-oven/prso-v1-br.php
iHILIC-(P) Classic HPLC column (100 × 2.1 mm, 5 μm, 200 Å)	HILICON AB	HIL160-102-0520
BEH-C18 column (2.1 × 150 mm, 1.7 μm)	Waters	186009454
Double nanoViper PepMap C18 analytical column (500 mm × 75 μm ID, 2 μm, 100 Å)	Thermo Fisher Scientific	DNV75500PN
PepSep sprayer 1 with 10 μm fused silica emitter	Bruker	PN 1893527
Monolith column (LC-UV, peptide quantification)	Thermo Scientific	Technical note 72602
Vanquish Neo UHPLC system	Thermo Fisher Scientific	VN-S10-A-01
UltiMate 3000 RSLCnano system	Thermo Scientific	
Ultimate 3000 HPLC system (metabolomics)	Thermo Fisher Scientific	
timsTOF HT mass spectrometer	Bruker Daltonics	
CaptiveSpray nano-ESI source	Bruker Daltonics	
Orbitrap Exploris 480 mass spectrometer	Thermo Fisher Scientific	
Q-Exactive Focus mass spectrometer	Thermo Fisher Scientific	
1290 Infinity II UHPLC system (lipidomics)	Agilent	
6560 IM-QTOF-MS (lipidomics)	Agilent	
Human UniProt database (20230710, 20,586 sequences)	UniProt Consortium	https://www.uniprot.org/
LinkedOmics portal (CPTAC ccRCC proteomic data)	LinkedOmics	http://linkedomics.org/
mzCloud database (metabolite annotation)	Thermo Fisher Scientific	https://www.mzcloud.org/

### Patient cohorts

#### Discovery cohort

The study was approved by the ethical commission at the Medical University of Vienna (Ethik-Kommission Medizinische Universität Wien), study number 2224/2021, project title: Urinproteomik zur Validierung von Biomarkern für das klarzellige Nierenkarzinom–Pilotstudie (UrineProt). Urine was collected from 40 patients who presented with a suspected primary renal mass at the Department of Urology of the Medical University of Vienna. Out of the 40 patients, 9 were excluded due to the renal mass being identified as something other than a renal cell carcinoma, such as oncocytomas, cysts, angiomyolipoma, papillary adenoma, or a kidney-lodged metastasis. From the remaining 31 patients, 22 patients were characterized as ccRCC, 8 as pRCC, and 1 as chRCC through histological assessment by a trained pathologist. For further analysis, the pRCC and chromophobe RCC patients were combined into one group of nccRCC patients. Leukocytes, creatinine, and C-reactive protein levels were obtained in the routine blood analysis using standard methods at the Institute of Laboratory Medicine, Vienna General Hospital. All patients provided informed consent before donation, and all experiments were carried out in accordance with the Declaration of Helsinki and the Belmont Report. For all downstream experiments and analysis, experimenters were blinded to the disease or control state of individual samples (single blinding). Sample sizes were not pre-determined but rather allocated to groups depending on pathological assessment.

#### Validation cohort

The samples were provided by the BioBank Hospital Ramón y Cajal-IRYCIS (National Registry of Biobanks B.0000678), integrated into the Biobanks and Biomodels Platform of the ISCIII (PT23/00098), with the approval of both the Ethical and Scientific Committees, study number 21_2024, and were processed following standard operating procedures. The cohort consisted of 13 RCC patients and 16 controls. Three RCC samples were excluded due to one of them being a chromophobe subtype, one being a granular eosinophilic type, and the other being in a metastatic setting involving the breast with a history of chemotherapy. The 16 controls were healthy blood donors at the hospital. Upon urine donation at the hospital, the urine was centrifuged at 1500 × *g* at 4 °C for 10 min and stored at −80 °C. All patients provided informed consent before donation, and all experiments were carried out in accordance with the Declaration of Helsinki and the Belmont Report. For all downstream experiments and analysis, experimenters were blinded to the disease or control state, and other factors relating to the validation cohort, of individual samples (single blinding).

### Protein precipitation and protein digestion

In all, 3 ml of ccRCC patient, nccRCC patient, or control urine was centrifuged at 1000 × *g* for 10 min. The urine supernatant was isolated and mixed with 12 ml of acetone and incubated for a minimum of 2 h at −20 °C to allow for protein precipitation. Samples were centrifuged at 3000 × *g* for 60 min and the supernatant discarded. The protein pellet was then resuspended in 100 μl of 8 M Urea, and a second precipitation step was performed to increase protein yields and to discard residual contaminants using chloroform and methanol (Wessel and Flügge, 1984). In total, 400 μl methanol was added to the protein solution and vortexed extensively. Subsequently, 200 μl of chloroform and 300 μl of water were added with vortexing steps in between. The mixtures were centrifuged at 10,000 × *g* for 15 min at room temperature to ensure phase separation. Following centrifugation, the top aqueous layer was removed, leaving the lower chloroform fraction and white protein interface. To the remaining mixture, 400 μl of methanol was added, followed by vortexing and centrifugation at 10,000 × *g* for 5 min. Supernatant was removed, and the methanol wash was repeated twice. After the last methanol wash, the pellet was dried in a speed vacuum and resuspended in 8 M Urea.

Precipitated proteins were reduced in 10 mM dithiothreitol (DTT, Roche) at 37 °C for 1 h and diluted to 4 M Urea. Following reduction, proteins were subsequently alkylated with 20 mM Indole-3-acetic acid (Merck) for 1 h at room temperature in the dark and diluted to 2 M Urea. Following reduction and alkylation, proteins were initially enzymatically digested with LysC (Lysyl Endopeptidase®, Mass Spectrometry Grade, FUJIFILM) at 37 °C for 2 h according to the manufacturer’s instructions. Lastly, peptides were further digested with trypsin (Trypsin Gold, Mass Spectrometry Grade, Promega) overnight at 37 °C according to the manufacturer’s instructions. 4/12 controls, 1/22 ccRCC, and 2/9 nccRCC urine sediment samples did not produce sufficient peptide amounts for reliable MS analysis.

### Bulk proteomics

#### Data-independent acquisition mass spectrometry

Individual peptide samples were analyzed by liquid chromatography-tandem mass spectrometry (LC–MS/MS). The nano high-performance liquid chromatography (HPLC) system used was a Thermo Scientific UltiMate 3000 RSLCnano (Thermo Scientific) equipped nano-electrospray source (CaptiveSpray source, Bruker Daltonics), coupled to a trapped ion mobility spectrometry time-of-flight high-throughput (timsTOF HT) mass spectrometer (Bruker Daltonics). Peptide samples were injected on a pre-column (PepMap C18, 5 mm × 300 μm × 5 μm, 100 Å pore size, Thermo Scientific) with 2% acetonitrile (CAN)/water (v/v) containing 0.1% trifluoroacetic acid (TFA) at a flow rate of 10 μL/min for 10 min. Peptides were then separated on a 25 cm Aurora ULTIMATE series HPLC column equipped with an emitter (CSI, 25 cm × 75 µm ID, 1.7 µm C18, IonOpticks) operating at 50 °C and controlled by the Column Oven PRSO-V1-BR (Sonation), using UltiMate 3000 (Thermo Scientific Dionex). The analytical column flow was run at 300 nL/min with two mobile phases: water with 0.1% formic acid (FA, A) and water with 80% ACN and 0.08% FA (B). A and B were applied in linear gradients as follows (only B percentages reported): starting from 2% B: 2%–10% B in 10 min, 10%–24% B in 35 min, 24%–35% B in 15 min, 35%–95% B in 1 min, 95% for 5 min, and finally the column was equilibrated in 2% B for next 10 min (all % values are v/v; Water and ACN solvents were purchased from Thermo Fisher Scientific at LC–MS grade).

The LC system was coupled to a quadrupole timsTOF HT mass spectrometer (Bruker Daltonics), and samples were measured in data-independent acquisition parallel accumulation-serial fragmentation (DIA-PASEF) mode. The CaptiveSpray source parameters were: 1600 V capillary voltage, 3.0 l/min dry gas, and 180 °C dry temperature. MS data was acquired in the MS scan mode, using positive polarity, 100–1700 *m/z* range, mobility range was set up from 0.64 to 1.42 V s/cm^2^, ramp time was set to 166 ms, and the estimated cycle time was 1.52 s. Collision energy was 20 eV at 1/K_0_ 0.6 V s/cm^2^, and 80 eV at 1/K_0_ 1.6 V s/cm^2^. Automatic calibration of ion mobility was enabled. The timsTOF HT was operated in data-independent acquisition (DIA) mode, where 1 MS1 scan was followed by 8 DIA-PASEF frames.

#### Proteomics data analysis

DIA data were analyzed in Spectronaut 18.5 (Bruderer et al, 2015) (Biognosys). Trypsin/P was specified as a proteolytic enzyme, and up to 2 missed cleavages were allowed in the Pulsar direct DIA search. Dynamic mass tolerance was applied for the TOF calibration. Peptides were matched against the human UniProt database (20230710, 20 586 sequences), with common contaminants (344 sequences) and common tags (28 sequences) appended. Carbamidomethylation of cysteine was searched as a fixed modification, whereas oxidation of methionine and acetylation at protein N-termini were defined as variable modifications. Peptides with a length between 7 and 52 amino acids were considered, and results were filtered for 1% false discovery rate (FDR) at the peptide spectrum match (PSM), Peptide and Protein Group Level. Quantification was performed as specified in Biognosys BGS Factory Default settings, grouping Peptides by Stripped Sequence, and performing protein inference using IDPicker. For normalization, Cross-Run Normalization in Spectronaut was activated.

Spectronaut results were exported using Pivot Reports on the Protein and Peptide level and converted to Microsoft Excel files using our in-house software MS2Go. For DIA data, MS2Go utilizes the Python library msReport (developed at the Max Perutz Labs Proteomics Facility) for data processing. Missing values were imputed with values obtained from a log-normal distribution with a mean of 30 in msReport, and statistical significance of differentially expressed proteins was determined using limma-moderated Benjamini–Hochberg-corrected two-sided *t* test (Smyth, 2004).

### Metabolomics

Metabolites were extracted from each sample by mixing 20 μl of urine supernatants with 200 μl methanol. Samples were subsequently dried down in a vacuum centrifuge and resuspended in 0.1% formic acid. Creatinine levels were determined in a targeted LC–MS/MS experiment. Normalized to the amount of creatinine determined, another aliquot of each extracted sample was evaporated and resuspended in 130 μl ACN:H_2_O (80:20). Samples were then centrifuged at 4 °C for 10 min at 16,000 × *g* and transferred to a glass HPLC vial. In all, 2 μl of all samples were pooled and used as a quality control (QC) sample. Samples were randomly assigned into the autosampler, and metabolites were separated on an iHILIC®-(P) Classic HPLC column (HILICON AB, 100 × 2.1 mm; 5 µm; 200 Å, Sweden) with a flow rate of 100 µl/min delivered through an Ultimate 3000 HPLC system (Thermo Fisher Scientific, Germany). The stepwise gradient started at 90% A (ACN) and took 21 min to 60% B (25 mM ammonium bicarbonate) followed by 5 min hold at 80% B and subsequent equilibration phase at 90% A with a total run time of 35 min. Sample spectra were acquired by a high-resolution tandem mass spectrometer (Q-Exactive Focus, Thermo Fisher Scientific, Germany) in full MS mode. Metabolites were ionized via electrospray ionization in polarity switching mode after Hydrophilic Interaction Liquid Chromatography (HILIC) separation. Ionization potential was set to +3.5/−3.0 kV, the sheet gas flow was set to 20, and an auxiliary gas flow of 5 was used. Samples were subjected to randomized analysis, flanked by a blank and a QC sample for background correction and data normalization, respectively, occurring after every set of eight samples. QC samples were additionally measured in data-dependent and confirmation mode to obtain MS/MS spectra for identification. The obtained dataset was processed by “Compound Discoverer 3.3 SP2” (Thermo Fisher Scientific). Compounds were annotated through searching against our internal mass list database, which was generated with authentic standard solutions. Additional compound annotation was conducted by searching the mzCloud database.

### Lipidomics

Lipids were extracted according to the Matyash protocol (Matyash et al, 2008) using 3 ml of Urinary supernatant. Internal standard mix (PE 34:0, 830456 P; PS 34:0, 840028 P; LPC 17:1, 855677 C; SM d35:1, 860585; purchased from Avanti Polar Lipids, USA, and PC 34:0, 37-1700-7; TG 54:0, 33-1835-9; purchased from Larodan, Sweden) was added to the samples before extraction. The organic phase of the final extraction was dried in a vacuum centrifuge and resolved in 500 µl of 2-propanol:MeOH:H_2_O (70:20:10, v:v:v) before injection.

Samples were analyzed with reversed phase-UHPLC (BEH-C18, 2.1 × 150 mm, 1.7 µm, Waters, Milford, USA) QTOF-MS (1290 Infinity II and 6560 IM-QTOF-MS, Agilent, Waldbronn, Germany) in positive/negative ESI QTOF-only mode. For the gradient elution, an aqueous eluent A and a 2-propanol eluent B were used, both with the following additives: Ammonium acetate (10 mM), phosphoric acid (8 μM), and FA (0.1 vol%). The gradient started with 60% eluent A for 0.5 min, followed by a linear decrease over 8.5 min to 20% and within 13 min to 0% A. This composition was held constant for 2.5 min and then returned to initial conditions for 5 min prior to the next injection. Eluent flow was constant at 150 µl/min. In positive mode 1 µl and in negative ion mode 5 µl were injected. Column temperature was 50 °C. The ESI instrument parameters were in positive mode: Gas temp 300 °C, flow 10 l/min, Nebulizer 50 psi, sheath gas temp 400 °C, flow 12 l/min), and in negative mode: Gas temp 300 °C, flow 5 l/min, Nebulizer 30 psi, sheath gas temp 350 °C, flow 12 l/min). The scan source parameters in pos and neg mode were (VCap 3500, Nozzle Voltage 500 V, Fragmentor 360, Skimmer 1, and OctopoleRFPeak 750). The data were annotated using MS-DIAL and its lipidomics database. For data integration and relative quantitation, we used Lipid Data Analyzer 2.8.3_2 (Hartler et al, 2017).

### TCGA survival analysis

TCGA clinical data were downloaded in RStudio with the *RTCGA* and *RTCGA.clinical* packages. The clinical data were analyzed using the *survival* and *survminer* packages and plotted using the *ggsurvplot* package in ggplot2.

### Parallel reaction monitoring mass spectrometry (PRM-MS)

#### Protein of interest peptide selection criteria

Peptides included in the PRM method were selected based on clean chromatographic elution profiles in the bulk proteomics data in ccRCC samples, reliable detection across samples, and the requirement of at least two unique peptides per protein of interest.

#### Relative peptide amount determination

Before NanoLC-MS/MS analysis, final peptide amounts were determined by separating an aliquot of each sample on an LC-UV system equipped with a monolith column (Thermo Scientific technical note 72602) and normalizing it to the peak area of 100 ng of Pierce HeLa protein digest standard (PN 88329; ThermoFisher Scientific).

#### NanoLC-MS/MS analysis

The nano HPLC system used was a Vanquish Neo ultra high-performance liquid chromatography (UHPLC) System coupled to an Orbitrap Exploris 480 mass spectrometer, equipped with a next-generation easy spray source (Thermo Fisher Scientific). Peptides were loaded onto a trap column (PepMap C18, 5 mm × 300 μm ID, 5 μm particles, 100 Å pore size, Thermo Fisher Scientific) by using 0.1% TFA. The trap column was switched in line with the analytical column (Double nanoViper™ PepMap C18, 500 mm × 75 μm ID, 2 μm, 100 Å, Thermo Fisher Scientific). The analytical column was connected to PepSep sprayer 1 (Bruker) equipped with a 10 μm ID fused silica electrospray emitter with an integrated liquid junction (Bruker, PN 1893527). Electrospray voltage was set to 2.3 kV. The analytical column flow was run at 230 nL/min, at 60 min binary gradient, with two mobile phases: water with 0.1% FA (A) and water with 80% ACN and 0.08% FA (B). A and B were applied in linear gradients as follows (only B percentages reported): starting from 2% B: 2%–10% B in 10 min, 10%–24% B in 35 min, 24%–35% B in 15 min, 35%–95% B in 1 min, 95% for 5 min, and finally the column was equilibrated in 2% B for 3 analytical column volumes at 30 °C (all % values are v/v; Water and ACN solvents were purchased from Thermo Fisher Scientific at LC–MS grade).

The Orbitrap Exploris 480 mass spectrometer was operated by a mixed MS method which consisted of one full scan (*m/z* range 380–1500; 15000 resolution; target value 100%) followed by the PRM of targeted peptides from an inclusion list (isolation window 0.8 *m/z*; normalized collision energy (NCE) 34; 30000 resolution, AGC target 200%). Spectra of unique peptides of the proteins of interest were recorded. For each protein, at least two unique peptides were measured. The maximum injection time was set to 125 ms. Each precursor was measured in a 5 min time window. Peptides included in the PRM method are listed in Table EV2. A scheduled PRM method (sPRM) development, data processing, and manual evaluation of results were performed in Skyline (MacLean et al, 2010) (64-bit, v22.2.0.351). To derive the PRM score for each protein of interest (POI), the area of each identified peptide (Peptide area, PA) was summed and divided by the sum of all areas from all identified peptides from the three normalization proteins using the following formula:

$$	{{\rm{PRM\; score}}}\,\left({{\rm{POI}}}\right)= \\ 	\frac{\sum {{\rm{PA}}}({{\rm{POI}}})}{\sum {{\rm{PA}}}\left({{\rm{UMOD}}}\right)+\sum {{\rm{PA}}}\left({{\rm{KLK}}}1\right)+\sum {{\rm{PA}}}({{\rm{APOD}}})}$$

### Protein structure visualization

The predicted protein structures of human HP, SAA1, and LCN15 were downloaded from the AlphaFold Protein Structure Database (https://alphafold.ebi.ac.uk/), and the detected peptides were highlighted on the three individual structures using ChimeraX (v1.12).

### UrineScore

The UrineScore was calculated in the following way: The PRM score of the three POIs was calculated for controls, nccRCC, and ccRCC patients. The 95% confidence interval (CI) of the median of the PRM scores was calculated for the three POIs for the controls. (i) Haptoglobin 95% CI of median = 0.003772 –  0.03508, (ii) SAA1 95% CI of median = 0 – 5.35E-9, (iii) LCN15 95% CI of median =  0 – 2.02E-5. Then, for each control and patient sample, the PRM scores for each POI were compared to the corresponding proteins 95% CI of the controls. If the PRM score was higher than the upper limit of the controls 95% CI, a value of 1 was attributed. This calculation was done for all three proteins, and the values were added together, meaning that each control and patient sample will receive an UrineScore, which is an integer between 0 and 3.

The UrineScore baselines were re-calculated for the validation cohort using exactly the same methodology and the following 95% CIs were obtained for the three POIs in this cohort: (i) Haptoglobin 95% CI of median = 0.004166 –  0.06186, (ii) SAA1 95% CI of median = 0 – 0 (only detected in two control samples), (iii) LCN15 95% CI of median = 0 – 3.422E-6.

### Diagnostic performance assessment

The diagnostic performance of the UrineScore was evaluated at three threshold levels (UrineScore ≥1, ≥2, and ≥3) in both the discovery and validation cohorts. At each threshold, samples were classified as positive (UrineScore ≥ threshold) or negative (UrineScore < threshold), and a 2 × 2 confusion matrix was constructed. Sensitivity was defined as the proportion of ccRCC patients correctly identified as positive (true positive rate), and specificity as the proportion of controls correctly identified as negative (true negative rate). Accuracy was calculated as the proportion of all samples correctly classified. Positive predictive value (PPV) and negative predictive value (NPV) were calculated both for the observed cohort prevalence and for a hypothetical population prevalence of 1%, the latter providing a more clinically relevant estimate of test performance in a screening context.

Exact 95% confidence intervals for sensitivity and specificity were calculated using the Clopper-Pearson method, which is preferred over normal approximation intervals at small sample sizes. Briefly, given x correct classifications out of n cases in the relevant group (ccRCC patients for sensitivity, controls for specificity), the lower and upper bounds of the 95% CIs were derived from the 2.5th and 97.5th percentiles of the Beta distribution: lower =  β(α/2; x, *n* − x + 1) and upper = β(1−α/2; x + 1, *n* − x), where α = 0.05. All calculations were performed in R (v4.5) using the scipy.stats.beta distribution via the binom.test function equivalent.

### TCGA and CPTAC UrineScore in silico validation

RNA-sequencing data and matched clinical annotations for ccRCC (TCGA-KIRC), pRCC (TCGA-KIRP), and chromophobe renal cell carcinoma (TCGA-KICH) were obtained from The Cancer Genome Atlas (TCGA) via the TCGAbiolinks R package (v2.4). STAR-aligned raw counts were downloaded for all available primary tumor and matched solid tissue normal controls and processed using DESeq2 (v1.52) for size factor estimation and differential expression analysis. A single DESeq2 object was constructed from the full count matrix, filtered to retain genes with summed counts ≥10 across all samples, and used for all downstream analyses to ensure consistency of normalization. Differential expression of HP, SAA1, and LCN15 between tumor and normal-adjacent tissue was assessed using the Wald test with Benjamini–Hochberg correction for multiple testing.

Proteomic data for the CPTAC ccRCC cohort were obtained from the LinkedOmics portal. Normalized protein abundance values were used directly without further transformation. Differential protein abundance between tumor and normal tissue was assessed using the Wilcoxon rank-sum test. As LCN15 was not detectable in the CPTAC tissue proteome, survival analysis for the CPTAC cohort was restricted to the two-protein signature of HP and SAA1. All statistical analyses were performed in R (v4.5).

### Statistical analysis

All statistical analyses were performed in Prism 10 (GraphPad) or in RStudio (Posit). When comparing large omics datasets (proteomics, lipidomics or metabolomics) *P* values were calculated with limma-moderated Benjamini–Hochberg-corrected two-sided *t* test. For comparisons of individual markers between groups, the distribution of the data was initially determined by the Shapiro–Wilk normality test. All individual marker comparisons shown in the paper did not pass the Shapiro–Wilk normality, and subsequent analysis was either performed using Mann–Whitney *U* tests (two groups) or Kruskal–Wallis test with Dunn’s multiple comparisons test (three groups). Receiver operating characteristic (ROC) curves were constructed, and areas under the curve (AUROC) were calculated using the pROC package, with 95% confidence intervals derived by bootstrap resampling (2000 iterations). Gene Ontology analysis was performed using online portals, Enrichr for proteomics data and MetaboAnalyst for metabolomics data. To evaluate the stability of the binary scoring approach and provide a bias-corrected performance estimate, a logistic regression model was fitted in the discovery cohort using the three continuous, log₁₀-transformed PRM scores as predictors (R function glm, family = binomial). Prior to transformation, a minimal offset equal to one-tenth of the minimum non-zero observed value across all three markers (2.05 × 10⁻¹⁰) was added to each score to accommodate zero values while preserving the dynamic range of the data. Given the small sample size of the discovery cohort (*N* = 34), leave-one-out cross-validation (LOOCV) was applied to obtain an unbiased estimate of discriminatory performance: at each iteration, the model was re-fitted on *N* − 1 samples and the predicted probability obtained for the excluded sample, yielding a cross-validated AUC of 0.879. The fixed model trained on the full discovery cohort was subsequently applied without refitting to the independent validation cohort (*N* = 26) to assess external generalizability. Statistical comparison of AUC values between models was performed using the DeLong test. To evaluate multi-omics integration, a logistic regression model incorporating the three protein PRM scores and four phosphatidylcholine lipid species (PC 38:3, PC 38:4, PC 38:5, PC 38:6) was fitted and evaluated using LOOCV as described above. Lipid abundance values were log₂-transformed with an offset of 0.001 prior to modeling. Statistical comparison of AUC values between the integrated and protein-only models was performed using the DeLong test. Principal component analysis was performed on the scaled and mean-centered integrated feature matrix using the prcomp function in R. Group separation was assessed visually using 95% confidence ellipses. A significance threshold of *P*adj <0.05 was applied throughout unless otherwise stated.

### Graphics

Figures 1A,G, 3C, 4A, 5A and Synopsis graphics were created with BioRender.com.

## Supplementary information

Table EV1

Table EV2

Table EV3

Table EV4

Peer Review File

Dataset EV1

Dataset EV2

Dataset EV3

Dataset EV4

Source data Fig. 1

Source data Fig. 2

Source data Fig. 4

Source data Fig. 5

Expanded View Figures

## Data Availability

Raw mass spectrometry proteomics data for urine supernatants, urine sediments, and PRM-MS have been deposited in the PRIDE database with accession number PXD078983. Raw mass spectrometry metabolomics data have been deposited in the MetaboLights database with the accession number MTBLS12025. Raw mass spectrometry lipidomics data have been deposited in the MetaboLights database with the accession number MTBLS14671. The source data of this paper are collected in the following database record: biostudies:S-SCDT-10_1038-S44321-026-00498-2.
