# Supplementary material for: Urinary multi-omics reveal non-invasive diagnostic biomarkers in clear cell renal cell carcinoma
Source: EMBO Mol Med. 2026 Aug 11;18(9):3649–76. doi: 10.1038/s44321-026-00498-2 (PMC13562635; doi:10.1038/s44321-026-00498-2)
Supplement: Supplementary file 1 [file 44321_2026_498_MOESM14_ESM.pdf]

Expanded View Figures

Figure EV1. Discovery cohort patient recruitment and urine precipitation.

(**A**) Flowchart describing the recruitment of patients and controls into the discovery cohort. Urine was collected from 40 patients presenting in the clinic with a foreign kidney mass. After pathological assessment, 22 patients were assigned to the ccRCC group and 9 patients to the nccRCC group, 9 patients were excluded due to the masses being non-malignant, e.g., cysts. In parallel, 12 healthy age-matched controls were recruited. ccRCC clear cell renal cell carcinoma, nccRCC non-clear cell renal cell carcinoma. (**B**) Number of identified unique proteins (left) and unique peptides (right) in a healthy control urine supernatant sample using three different protein precipitation methods. (i) Precipitation only with acetone, (ii) precipitation only with chloroform and methanol, and (iii) double protein precipitation with acetone followed by chloroform and methanol. Two technical replicates per sample is shown. MeOH Methanol.

© The Author(s)

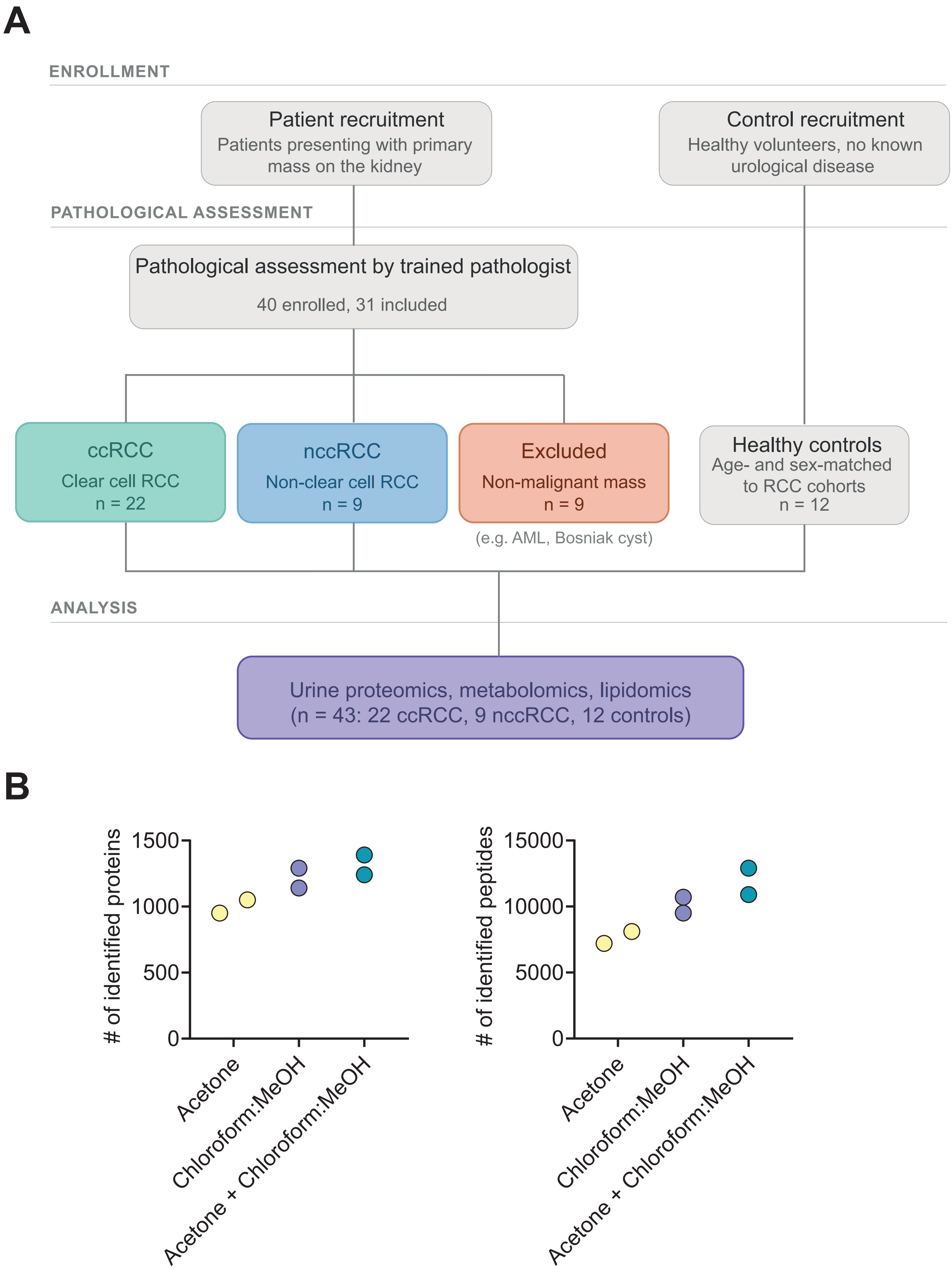
A
ENROLLMENT
Patient recruitment
Patients presenting with primary mass on the kidney
Control recruitment
Healthy volunteers, no known urological disease
PATHOLOGICAL ASSESSMENT
Pathological assessment by trained pathologist
40 enrolled, 31 included
ccRCC
Clear cell RCC
n = 22
nccRCC
Non-clear cell RCC
n = 9
Excluded
Non-malignant mass
n = 9
(e.g. AML, Bosniak cyst)
Healthy controls
Age- and sex-matched to RCC cohorts
n = 12
ANALYSIS
Urine proteomics, metabolomics, lipidomics
(n = 43: 22 ccRCC, 9 nccRCC, 12 controls)
B
# of identified proteins
1500
1000
500
0
Acetone
Chloroform:MeOH
Acetone + Chloroform:MeOH
# of identified peptides
15000
10000
5000
0
Acetone
Chloroform:MeOH
Acetone + Chloroform:MeOH

© The Author(s)

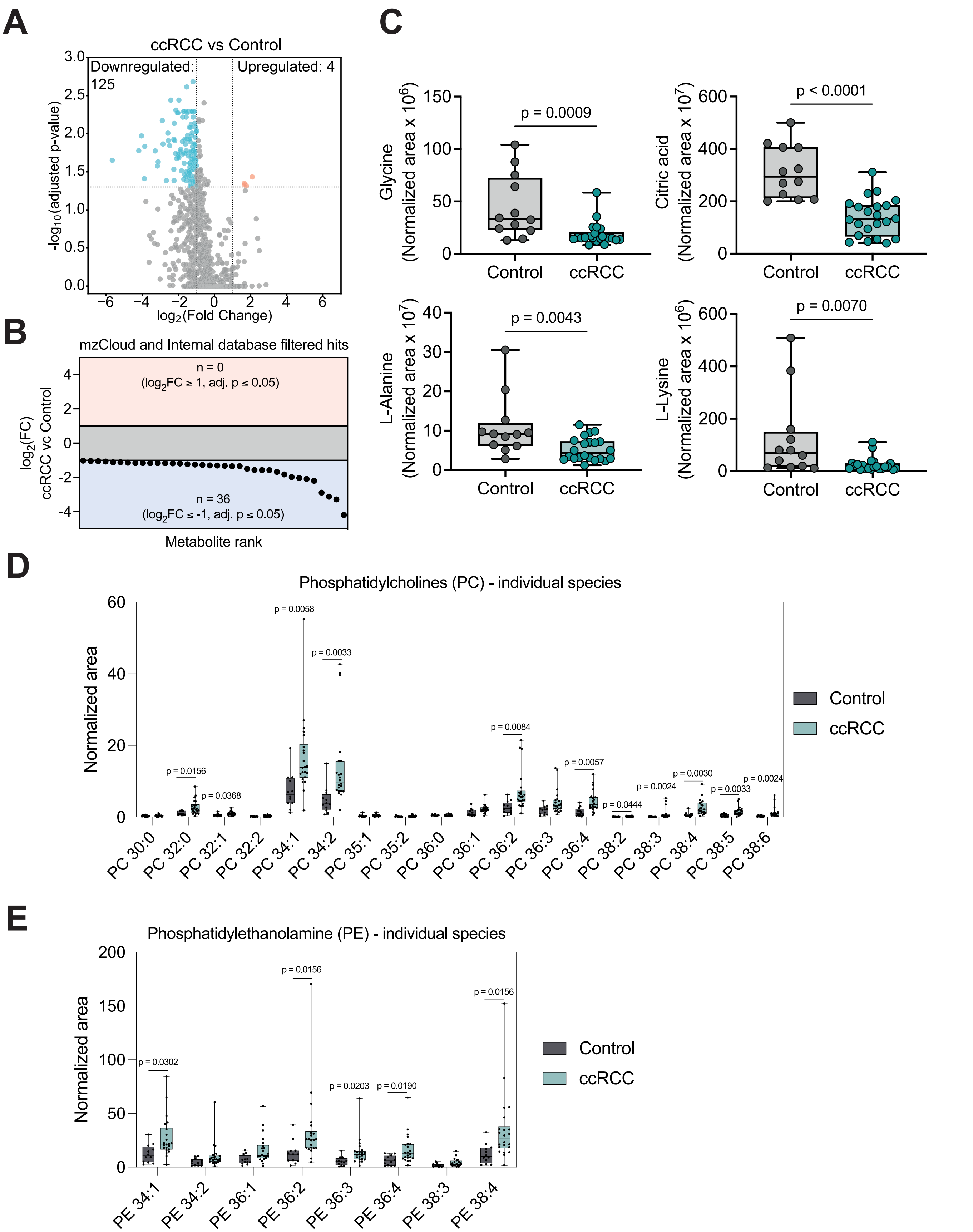
A
ccRCC vs Control
Downregulated: 125
Upregulated: 4
-log10(adjusted p-value)
log2(Fold Change)
B
mzCloud and Internal database filtered hits
n = 0
(log2FC ≥ 1, adj. p ≤ 0.05)
n = 36
(log2FC ≤ -1, adj. p ≤ 0.05)
log2(FC) ccRCC vc Control
Metabolite rank
C
Glycine (Normalized area x 10^6)
p = 0.0009
Citric acid (Normalized area x 10^7)
p < 0.0001
L-Alanine (Normalized area x 10^7)
p = 0.0043
L-Lysine (Normalized area x 10^6)
p = 0.0070
Control
ccRCC
D
Phosphatidylcholines (PC) - individual species
Normalized area
p = 0.0058
p = 0.0033
p = 0.0156
p = 0.0368
p = 0.0084
p = 0.0057
p = 0.0444
p = 0.0024
p = 0.0030
p = 0.0033
p = 0.0024
PC 30:0
PC 32:0
PC 32:1
PC 32:2
PC 34:1
PC 34:2
PC 35:1
PC 35:2
PC 36:0
PC 36:1
PC 36:2
PC 36:3
PC 36:4
PC 38:2
PC 38:3
PC 38:4
PC 38:5
PC 38:6
Control
ccRCC
E
Phosphatidylethanolamine (PE) - individual species
Normalized area
p = 0.0302
p = 0.0156
p = 0.0203
p = 0.0190
p = 0.0156
PE 34:1
PE 34:2
PE 36:1
PE 36:2
PE 36:3
PE 36:4
PE 38:3
PE 38:4
Control
ccRCC

Figure EV2. Metabolites and lipids in the urine of ccRCC patients.

(**A**) Volcano plot showing upregulated (red) and downregulated (blue) metabolites in ccRCC ($n = 22$) patients compared to controls ($n = 12$). Adjusted P values calculated via limma-moderated Benjamini–Hochberg-corrected two-sided t test. FC fold change. (**B**) Waterfall plot of significantly metabolites remaining when comparing control ($n = 12$) and ccRCC ($n = 22$) urine after filtering all hits from (**A**) through the mzCloud and an internal database. 36 significantly downregulated metabolites remain. (**C**) Boxplots showing the expression levels of 4 representative metabolites (glycine, citric acid, L-alanine, and L-lysine), as determined by metabolomics for controls ($n = 12$) and ccRCC ($n = 22$). Statistical significance assessed through limma-moderated Benjamini–Hochberg-corrected two-sided t test. The line in the middle of the box denotes the mean of the data, whereas the lower and upper boundaries of the box represent the 25th (Q1) and 75th (Q3) percentiles, respectively. (**D**) Boxplots of individual lipid species from the PC family for controls ($n = 12$) and ccRCC ($n = 22$). PC phosphatidylcholines. *$P < 0.05$, **$P < 0.01$. Statistical significance assessed through limma-moderated Benjamini–Hochberg-corrected two-sided t test. The line in the middle of the box denotes the mean of the data, whereas the lower and upper boundaries of the box represent the 25th (Q1) and 75th (Q3) percentiles, respectively. (**E**) Boxplots of individual lipid species from the PE family for controls ($n = 12$) and ccRCC ($n = 22$). PE phosphatidylethanolamine. *$P < 0.05$. Statistical significance assessed through limma-moderated Benjamini–Hochberg-corrected two-sided t test. The line in the middle of the box denotes the mean of the data, whereas the lower and upper boundaries of the box represent the 25th (Q1) and 75th (Q3) percentiles, respectively.

 © The Author(s)

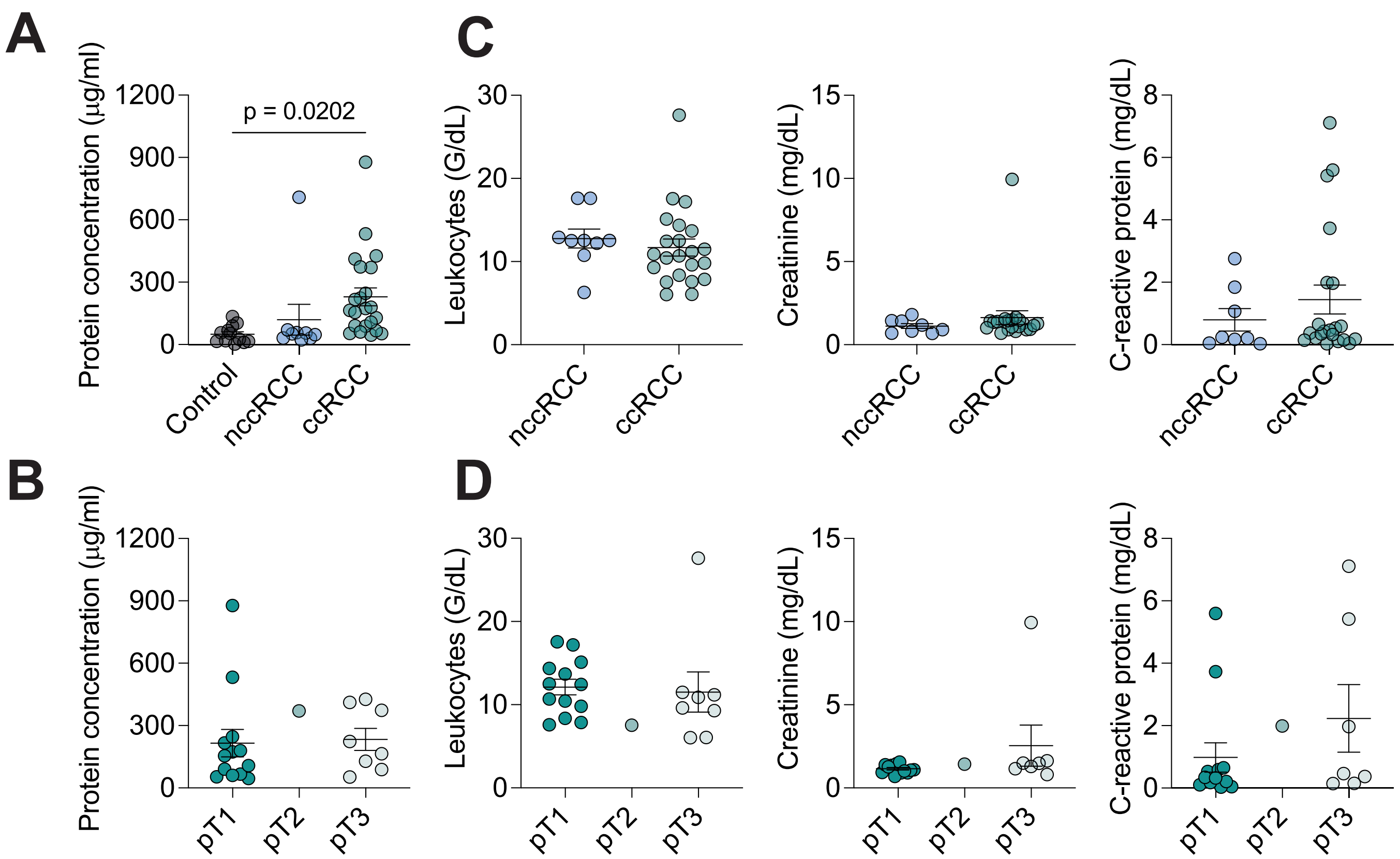

Figure EV3. Clinical cohort overview data.

(**A**) Protein concentration in urine supernatant from control ($n = 12$), nccRCC ($n = 9$) patients, and ccRCC ($n = 22$) patients determined via Bradford protein assay. Statistical significance assessed through the Kruskal–Wallis test with Dunn's multiple comparisons test. The mean is shown ± SEM. (**B**) Protein concentration in urine supernatant from ccRCC ($n = 22$) patients divided into pT1 ($n = 13$), pT2 ($n = 1$), and pT3 ($n = 8$) stages determined via Bradford protein assay. pT1 = tumor stage 1. The tumor is a maximum of 7 cm across. pT2 = tumor stage 2. The tumor is larger than 7 cm across. pT3 = tumor stage 3. The tumor has grown into a major renal vein (e.g., vena cava or renal vein) or into neighboring tissues but has not spread past Gerota's fascia or into the adrenal gland. The mean is shown ± SEM. (**C**) Leukocyte, Creatinine, and C-reactive protein levels in serum, respectively, for nccRCC ($n = 9$) patients and ccRCC ($n = 22$) patients. The mean is shown ± SEM. (**D**) Leukocyte, Creatinine, and C-reactive protein levels in serum of ccRCC ($n = 22$) patients, stratified at pT1 ($n = 13$), pT2 ($n = 1$), and pT3 ($n = 8$) stages. pT1 = tumor stage 1. The tumor is a maximum of 7 cm across. pT2 = tumor stage 2. The tumor is larger than 7 cm across. pT3 = tumor stage 3. The tumor has grown into a major renal vein (e.g., vena cava or renal vein) or into neighboring tissues but has not spread past Gerota's fascia or into the adrenal gland. The mean is shown ± SEM.

© The Author(s)

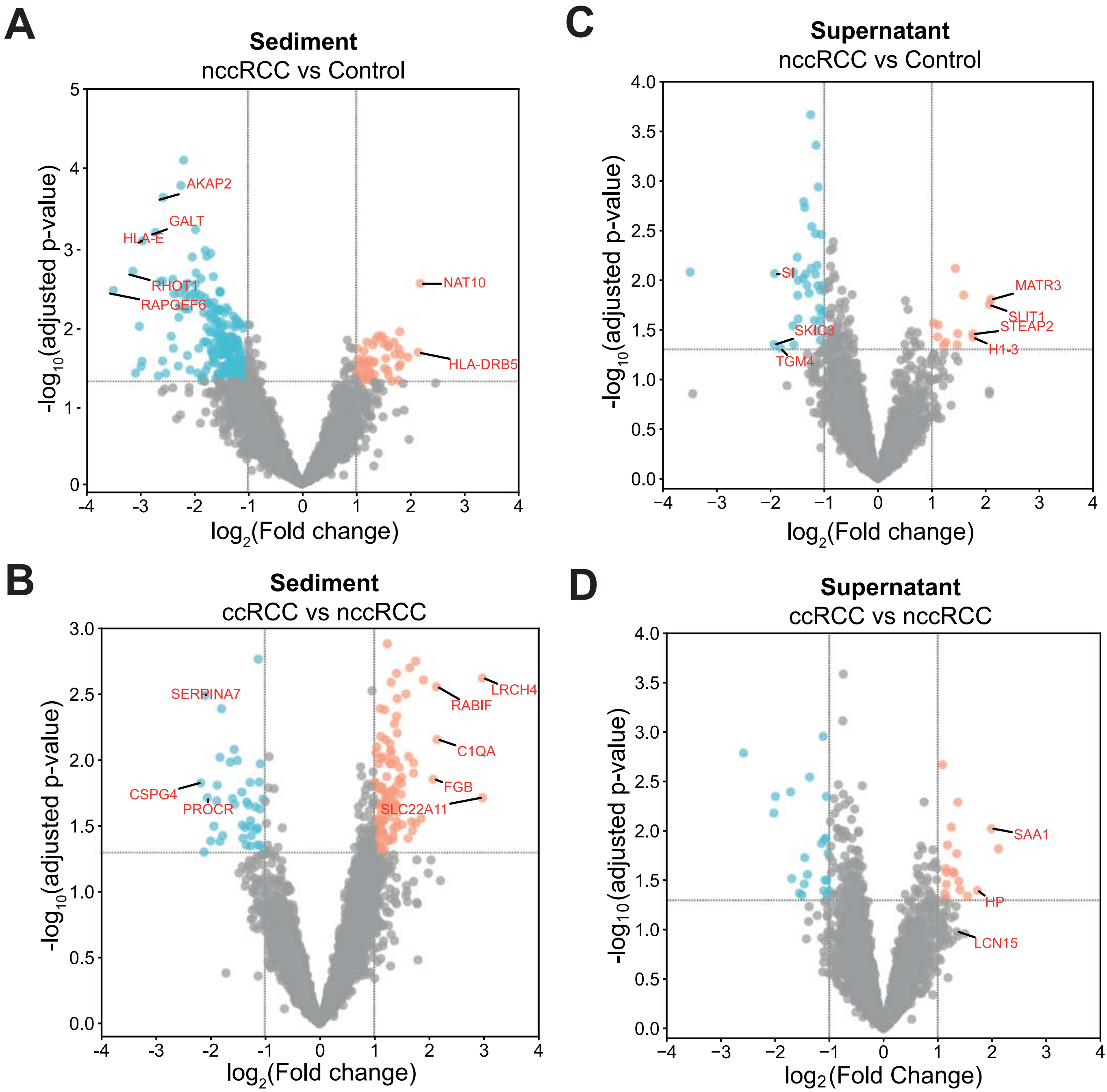

Figure EV4. Distinct urinary protein landscapes in ccRCC and nccRCC patients.

(**A**) Volcano plot showing upregulated (red) and downregulated (blue) proteins in nccRCC ($n = 7$) patients compared to controls ($n = 8$) in urine sediment. Adjusted *P* values calculated via limma-moderated Benjamini–Hochberg-corrected two-sided *t* test. (**B**) Volcano plot showing upregulated (red) and downregulated (blue) proteins in nccRCC ($n = 7$) patients compared to ccRCC ($n = 21$) patients in urine sediment. Adjusted *P* values calculated via limma-moderated Benjamini–Hochberg-corrected two-sided *t* test. (**C**) Volcano plot showing upregulated (red) and downregulated (blue) proteins in nccRCC ($n = 9$) patients compared to controls ($n = 12$) in urine
Q11 supernatant. Adjusted *P* values calculated via limma-moderated Benjamini–Hochberg-corrected two-sided *t* test. FC fold change. (**D**) Volcano plot showing upregulated (red) and downregulated (blue) proteins in ccRCC ($n = 22$) patients compared to nccRCC ($n = 9$) patients in urine supernatant. Adjusted *P* values calculated via limma-moderated Benjamini–Hochberg-corrected two-sided *t* test. FC fold change.

 © The Author(s)

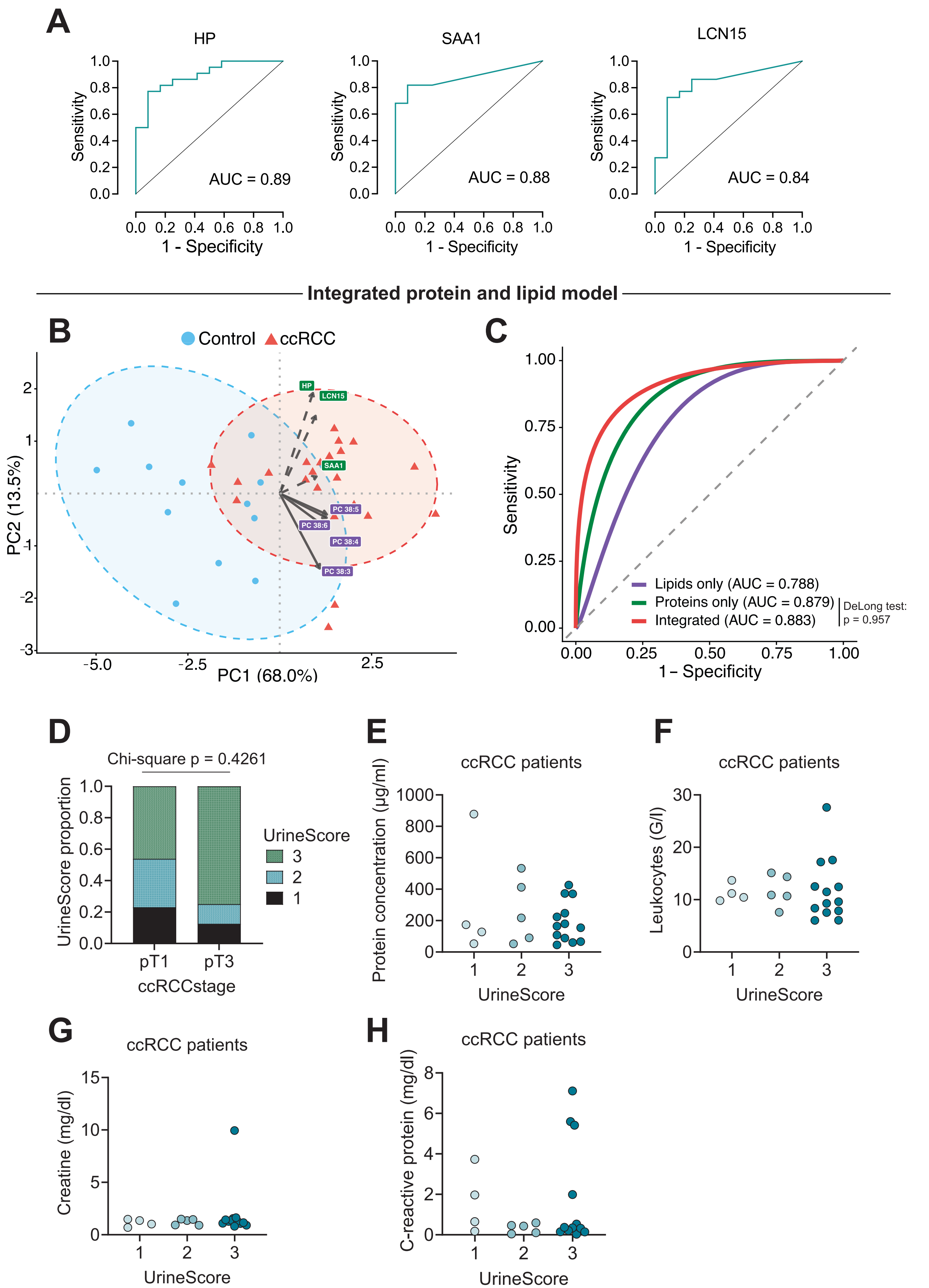
A
HP
SAA1
LCN15
Sensitivity
1 - Specificity
AUC = 0.89
AUC = 0.88
AUC = 0.84
Integrated protein and lipid model
B
Control
ccRCC
HP
LCN15
SAA1
PC 38:5
PC 38:6
PC 38:4
PC 38:3
PC2 (13.5%)
PC1 (68.0%)
C
Sensitivity
1 – Specificity
Lipids only (AUC = 0.788)
Proteins only (AUC = 0.879)
Integrated (AUC = 0.883)
DeLong test:
p = 0.957
D
Chi-square p = 0.4261
UrineScore proportion
UrineScore
pT1
pT3
ccRCCstage
E
ccRCC patients
Protein concentration (µg/ml)
UrineScore
F
ccRCC patients
Leukocytes (G/l)
UrineScore
G
ccRCC patients
Creatine (mg/dl)
UrineScore
H
ccRCC patients
C-reactive protein (mg/dl)
UrineScore

Figure EV5. UrineScore components are detectable with PRM-MS in the urine of ccRCC patients and do not correlate with clinical parameters.

(**A**) Receiver operating characteristic curve to assess the performance of the PRM score of Haptoglobin, SAA1, and LCN15, respectively, in differentiating between control ($n = 12$) and ccRCC ($n = 22$) samples. Performance quantified through the area under the receiver operating characteristic curve (AUC) analysis. (**B**) Principal component analysis (PCA) of the integrated feature matrix comprising the three UrineScore protein PRM scores (HP, SAA1, LCN15) and the four best-performing urinary phosphatidylcholine species (PC 38:3, PC 38:4, PC 38:5, PC 38:6) in the discovery cohort ($N = 34$; 22 ccRCC, 12 controls). All features were log-transformed prior to analysis, and the feature matrix was mean-centered and scaled to unit variance. PC1 and PC2 explain 68.0% and 13.5% of the total variance, respectively. Data points represent individual samples; ellipses indicate 95% confidence regions. Arrows indicate feature loadings; solid arrows denote lipid species; dashed arrows denote protein markers. (**C**) Receiver operating characteristic (ROC) curves comparing discriminatory performance of three logistic regression models evaluated by leave-one-out cross-validation (LOOCV) in the discovery cohort: lipid species only (PC 38:3, PC 38:4, PC 38:5, PC 38:6; AUC = 0.788), UrineScore proteins only (HP, SAA1, LCN15; AUC = 0.879), and an integrated model incorporating all seven features (AUC = 0.883). Statistical comparison of the integrated and protein-only models was performed using the DeLong test ($P = 0.957$). ROC curves are smoothed using a binormal model for visualization; reported AUC values reflect empirical estimates. All features were log-transformed prior to modeling using the same offsets described in the "Methods". (**D**) Stacked bar plots showing the distribution of the UrineScore in stage 1 (pT1, $n = 13$) and stage 3 (pT3, $n = 8$) tumors. Stage 2 tumors are excluded from this analysis due to there only being $n = 1$ tumor in this group. Statistical significance assessed through the chi-square test. (**E**) Scatter plot showing the distribution of urinary protein concentrations (measured using BCA) for ccRCC samples scoring 1 ($n = 4$), 2 ($n = 5$), or 3 ($n = 13$) in the UrineScore. Statistical significance assessed through one-way ANOVA. (**F**) Scatter plot showing the distribution of circulating leukocytes for ccRCC samples scoring 1 ($n = 4$), 2 ($n = 5$), or 3 ($n = 12$, leukocytes were not assessed for one UrineScore = 3 sample, in the UrineScore. Statistical significance assessed through one-way ANOVA. (**G**) Scatter plot showing the distribution of serum creatinine levels for ccRCC samples scoring 1 ($n = 4$), 2 ($n = 5$), or 3 ($n = 12$, creatinine was not assessed for one UrineScore = 3 sample) in the UrineScore. Statistical significance assessed through one-way ANOVA. (**H**) Scatter plot showing the distribution of serum C-reactive protein levels for ccRCC samples scoring 1 ($n = 4$), 2 ($n = 5$), or 3 ($n = 12$, C-reactive protein was not assessed for one UrineScore = 3 sample) in the UrineScore. Statistical significance assessed through one-way ANOVA.

 © The Author(s)
