# Supplementary material for: Urinary multi-omics reveal non-invasive diagnostic biomarkers in clear cell renal cell carcinoma
Source: EMBO Mol Med. 2026 Aug 11;18(9):3649–76. doi: 10.1038/s44321-026-00498-2 (PMC13562635; doi:10.1038/s44321-026-00498-2)
Supplement: Supplementary file 2 [file 44321_2026_498_MOESM5_ESM.pdf]

EMBO Molecular Medicine

Urinary multi-omics reveal non-invasive diagnostic biomarkers in clear cell renal cell carcinoma

Gustav Jonsson, Tiago Oliveira, Maura Hofmann, Ursula Lemberger, Karel Stejskal, Gabriela Krššáková, Irma Sakic, Maria Novatchkova, Stefan Mereiter, Gerlinde Grabmann, Thomas Koecher, Rubina Koglgruber, Zeljko Kikic, Sonia Camano Páez, Bárbara Luna Sanches, Víctor Díez Nicolás, Gerald Rechberger, Thomas Züllig, Astrid Hagelkruys, Bernhard Englinger, Manuela Schmidinger, and Josef Penninger

Corresponding authors: Josef Penninger (josef.penninger@helmholtz-hzi.de) , Gustav Jonsson (gustav.jonsson@medunigraz.at)

Review Timeline:

Submission Date:	13th Jan 26
Editorial Decision:	23rd Feb 26
Revision Received:	12th Jun 26
Editorial Decision:	9th Jul 26
Revision Received:	16th Jul 26
Accepted:	22nd Jul 26

Editor: Lise Roth

Transaction Report:

(Note: With the exception of the correction of typographical or spelling errors that could be a source of ambiguity, letters and reports are not edited. Depending on transfer agreements, referee reports obtained elsewhere may or may not be included in this compilation. Referee reports are anonymous unless the Referee chooses to sign their reports.)

23rd Feb 2026

Dear Dr. Penninger,

Thank you for submitting your manuscript to our editorial office, and please accept my apologies for the delay in getting back to you, as one referee needed more time to complete their report. We have now received feedback from the three reviewers who agreed to evaluate your study. As you will see from the reports below, while they acknowledge the potential interest of the findings, they nevertheless raise several major concerns about the study. They agree in particular that the validation cohort should be expanded.

Addressing the reviewers' concerns in full will be necessary for further considering the manuscript in our journal, and acceptance of the manuscript will entail a second round of review. EMBO Molecular Medicine encourages a single round of revision only and therefore, acceptance or rejection of the manuscript will depend on the completeness of your responses included in the next, final version of the manuscript. For this reason, and to save you from any frustrations in the end, I would strongly advise against returning an incomplete revision.

If you would like to discuss further the points raised by the referees, I am available to do so via email or video. Let me know if you are interested in this option.

We are expecting your revised manuscript within three months, if you anticipate any delay, please contact us.

When preparing your revised manuscript, please refer to our guidelines: https://link.springer.com/journal/44321/submission-guidelines#cms-Revised-submissions. We perform an initial quality control of all revised manuscripts before re-review; failure to include requested items will delay the evaluation of your revision.

We require:

1) A .docx formatted version of the manuscript text (including legends for main figures, EV figures and tables). Please make sure that the changes are highlighted to be clearly visible.

2) Individual production quality figure files as .eps, .tif, .jpg (one file per figure). For guidance, download the 'Figure Guide PDF': https://media.springernature.com/original/springer-cms/rest/v1/content/27825798/data/v1.

3) A .docx formatted letter INCLUDING the reviewers' reports and your detailed point-by-point responses to their comments. As part of the EMBO Press transparent editorial process, the point-by-point response is part of the Review Process File (RPF), which will be published alongside your paper.

4) A complete author checklist, which you can download from our author guidelines. Please insert information in the checklist that is also reflected in the manuscript. The completed author checklist will also be part of the RPF.

5) Please note that all corresponding authors are required to supply an ORCID ID for their name upon submission of a revised manuscript.

6) It is mandatory to include a 'Data Availability' section after the Materials and Methods. Before submitting your revision, primary datasets produced in this study need to be deposited in an appropriate public database, and the accession numbers and database listed under 'Data Availability'. Please remember to provide a reviewer password if the datasets are not yet public.

In case you have no data that requires deposition in a public database, please state so in this section. Note that the Data Availability Section is restricted to new primary data that are part of this study.

7) For data quantification: please specify the name of the statistical test used to generate error bars and P values, the number (n) of independent experiments (specify technical or biological replicates) underlying each data point and the test used to calculate p-values in each figure legend. The figure legends should contain a basic description of n, P and the test applied. Graphs must include a description of the bars and the error bars (s.d., s.e.m.).

8) At EMBO Press we ask authors to provide source data for the main manuscript figures. You will receive a separate email with instructions for providing source data with your revised manuscript, including how to upload and organize the files.

9) Our journal encourages inclusion of *data citations in the reference list* to directly cite datasets that were re-used and obtained from public databases. Data citations in the article text are distinct from normal bibliographical citations and should directly link to the database records from which the data can be accessed. In the main text, data citations are formatted as

follows: "Data ref: Smith et al, 2001" or "Data ref: NCBI Sequence Read Archive PRJNA342805, 2017". In the Reference list, data citations must be labeled with "[DATASET]". A data reference must provide the database name, accession number/identifiers and a resolvable link to the landing page from which the data can be accessed at the end of the reference.

10) We replaced Supplementary Information with Expanded View (EV) Figures and Tables that are collapsible/expandable online. A maximum of 5 EV Figures can be typeset. EV Figures should be cited as 'Figure EV1, Figure EV2" etc... in the text and their respective legends should be included in the main text after the legends of regular figures.

- For the figures that you do NOT wish to display as Expanded View figures, they should be bundled together with their legends in a single PDF file called *Appendix*, which should start with a short Table of Content. Appendix figures should be referred to in the main text as: "Appendix Figure S1, Appendix Figure S2" etc.

- Additional Tables/Datasets should be labeled and referred to as Table EV1, Dataset EV1, etc. Legends have to be provided in a separate tab in case of .xls files. Alternatively, the legend can be supplied as a separate text file (README) and zipped together with the Table/Dataset file.

11) The paper explained: EMBO Molecular Medicine articles are accompanied by a summary of the articles to emphasize the major findings in the paper and their medical implications for the non-specialist reader. Please provide a draft summary of your article highlighting
- the medical issue you are addressing,
- the results obtained and
- their clinical impact.

This may be edited to ensure that readers understand the significance and context of the research. Please refer to any of our published articles for an example.

12) Author contributions: You will be asked to provide CRediT (Contributor Role Taxonomy) terms in the submission system. These replace a narrative author contribution section in the manuscript.

13) A "Disclosure and Competing Interests Statement" should be provided in the main text.

14) Every published paper includes a 'Synopsis' to further enhance discoverability. Synopses are displayed on the journal webpage and are freely accessible to all readers. They include a short stand first (maximum of 300 characters, including space) as well as 2-5 one-sentences bullet points that summarizes the paper. Please write the bullet points to summarize the key NEW findings. They should be designed to be complementary to the abstract - i.e. not repeat the same text. We encourage inclusion of key acronyms and quantitative information (maximum of 30 words / bullet point). Please use the passive voice. Please attach these in a separate file or send them by email, we will incorporate them accordingly.

Please also suggest a striking image or visual abstract to illustrate your article as a PNG file 550 px wide x 300-600 px high.

15) Include a Reagents and Tools Table as part of the Methods section, which can be downloaded from our author guidelines.

16) As part of the EMBO Publications transparent editorial process initiative (see our Editorial at http://embomolmed.embopress.org/content/2/9/329), EMBO Molecular Medicine will publish online a Review Process File (RPF) to accompany accepted manuscripts.
In the event of acceptance, this file will be published in conjunction with your paper and will include the anonymous referee reports, your point-by-point response and all pertinent correspondence relating to the manuscript. Let us know whether you agree with the publication of the RPF and as here, if you want to remove or not any figures from it prior to publication.
Please note that the Authors checklist will be published at the end of the RPF.

EMBO Molecular Medicine has a "scooping protection" policy, whereby similar findings that are published by others during review or revision are not a criterion for rejection. Should you decide to submit a revised version, I do ask that you get in touch after three months if you have not completed it, to update us on the status.

I look forward to receiving your revised manuscript.

Yours sincerely,

Lise Roth

Lise Roth, PhD
Senior Editor

***** Reviewer's comments *****

Referee #1 (Comments on Novelty/Model System for Author):

The discovery cohort is relatively small but may be suitable for selecting candidate markers. However, the validation cohort is far too small and was not biometrically planned based on the results of the discovery cohort. Therefore, the actual value of the markers cannot be definitively assessed.

Referee #1 (Remarks for Author):

The work of Jonsson et al. is dedicated to an interesting and important issue, namely the identification of potential tumor markers for clear cell renal cell carcinoma (ccRCC), for which no suitable biomarkers are currently available. The analysis of such markers in urine appears particularly attractive, as urine samples can be obtained non-invasively and repeatedly without great effort. By applying proteomic, lipidomic, and metabolomic analyses, the authors succeeded in identifying the three protein biomarkers serum amyloid A1 (SAA1), haptoglobin (HP) and lipocalin 15 (LCN15) in urine supernatant as promising candidates. They developed a diagnostic marker score from them that allows ccRCC patients to be distinguished from both healthy controls and patients with another RCC subtype (nccRCC).
Even though this approach appears to be very well thought out and comprehensive, and the complex methodology is described well in detail, the study has certain shortcomings.
On the one hand, there is no real need for a population-based screening for this type of tumor, but it would be very helpful, for example in patients with hematuria or suspicious renal masses, to confirm the suspected diagnosis with the aid of non-invasively determinable tumor markers. Therefore, control subjects with hematuria or other renal masses appeared to be more suitable than healthy subjects. For that reason, the use of healthy control subjects should be justified more comprehensively, as, in the reviewer's opinion, they do not necessarily represent a suitable control group. In addition, the controls should be matched to the patient cohort at least in terms of age and gender. However, no information on the control subjects is provided here. The authors should follow the reporting guidelines for diagnostic studies and provide at least a flow chart of cohort selection and study design.
The size of the discovery cohort is not very large, with 22 ccRCC patients and 12 control subjects. In particular, the comparison with only nine nccRCC patients (with only one chromophobe RCC) appears to be very limiting. In any case, the validation cohort is far too small. For a reliable statement, it must be expanded after biometric planning based on the results from the discovery cohort.
The authors draw interesting conclusions from the different protein patterns in urine supernatant and urine sediment. They suspect that electron transport chain phenotypes are linked to healthy, shed epithelium in the urine sediment and that upregulated respiratory chain pathways in the urine sediment most likely come from increased shedding of healthy epithelium or other shed urogenital cells. In this context, it would have been interesting to examine the urine sediments more closely for their cellular composition using urine cytology in order to prove that no or only a few RCC cells are detectable and which non-malignant cells dominate the sediment (epithelial cells, erythrocytes, immune cells?). This would have allowed potential correlations with protein and metabolite patterns to be evaluated.
Nevertheless, the associations between biomarkers at the protein and metabolite level in the various urine compartments (supernatant and cellular sediment) are interesting. Based on this, the authors draw comprehensible and insightful conclusions, which, however, should be examined and verified in greater depth in conjunction with the relevant literature. Overall, the discussion section of the manuscript primarily summarizes the results once again, but does not compare them in depth with those of other similar studies.
Since the authors have already used the TCGA data from the relevant RCC cohorts (no group sizes and references are given for these cohorts!) with regard to survival (it remains unclear whether this refers to overall survival), they should also evaluate their identified markers in these RCC cohorts compared to normal tissue. It would also be interesting to examine the prognostic relevance of the three markers for ccRCC based on TCGA data.
With regard to the diagnostic suitability and value of the three identified protein markers, their relevance in relation to ccRCC should also be discussed in more detail. There are a number of studies on SAA1 that demonstrate the prognostic potential of this factor in RCC. There are also reports on haptoglobin that should be examined more closely, e.g., the study by Tolson et al. (PMID: 15107802), which also identified SAA1 and haptoglobin in the blood of ccRCC patients. In the study by Sandim et al. (PMID: 26420021), which the authors briefly cite but do not discuss in detail, haptoglobin was also identified as differentially secreted protein in the urine of ccRCC patients by proteomic analysis. However, this study also identified uromodulin and apolipoprotein D (which serve as reference proteins herein) as differentially secreted proteins, which should be considered in more detail by the authors of this manuscript. In this context, the usefulness of uromodulin (also known as Tamm-Horsfall protein) should also be discussed more thoroughly. This protein is the most abundant protein in urine and shows values in urine

supernatant that are orders of magnitude higher than those of the three identified tumor markers (especially in comparison to SAA1 and LCN15), which may level out larger differences. This should be critically considered.
Several other studies conducted on urine samples using proteomics are mentioned, but their results are not compared with the obtained results. However, this is necessary for a better understanding of the significance of the reported findings. Technical aspects such as analysis and standardization approaches could also be discussed.
The classification of the markers using the described methodology is comprehensible and considers them independently of their protein concentration in urine supernatant, which varies considerably between the three markers. However, the authors do not propose specific cut-off values for distinguishing between the different groups, meaning that it is not really possible to assess the diagnostic performance (specificity, sensitivity, accuracy, PPV, NPV), even though the AUC values look promising.
Finally, the introduction of the abbreviations used should be reviewed in the methods section.

Referee #2 (Comments on Novelty/Model System for Author):

This is an interesting and technically solid multi-omics study with a pragmatic PRM-MS assay concept. However, before strong claims about screening/diagnosis are justified, the authors should temper conclusions and better address (i) small-cohort performance uncertainty, (ii) specificity against common urologic/renal confounders, and (iii) tissue-of-origin/biological plausibility for the proposed markers and the broader metabolic signatures.

Referee #2 (Remarks for Author):

Jonsson et al. report an integrated urine multi-omics stuy in ccRCC, identifying three urinary protein markers and deriving a PRM-MS-based composite "UrineScore" that discriminates ccRCC from controls. The clinical goal-non-invasive early detection-is important and clearly unmet. The multi-omics approach and targeted PRM workflow are strengths. However, key limitations around cohort size, specificity, and biological interpretability currently constrain confidence in clinical applicability.

Major concerns :
1. Cohort size and robustness of model performance
The cohorts are small, particularly for validation, which raises concerns about overestimation of diagnostic performance and limited generalizability. The discovery cohort includes 22 ccRCC and 12 controls, with an additional nccRCC group (n=9) for some analyses (pRCC n=8; chRCC n=1). The independent validation cohort includes 10 ccRCC and 16 controls (and excludes 3 RCC samples for histology/clinical reasons). Given these sample sizes, the reported AUROC/accuracy (e.g., UrineScore AUROC ~0.96 in discovery and ~0.95 in validation) may be optimistic, and confidence intervals should be emphasized (or added if missing), particularly for sensitivity/specificity at clinically relevant thresholds. The UrineScore construction uses a dichotomization approach relative to the control 95% CI of the median per marker, then sums the binary calls (0-3). This choice can be unstable in small cohorts and is sensitive to control distribution and pre-analytical factors.

2. Specificity and confounding by non-malignant renal/urologic conditions
A critical issue for any urine biomarker intended for screening is specificity versus common confounders (hematuria of non-malignant origin, nephrolithiasis, CKD, glomerular disease, inflammatory states). The authors acknowledge that the cellular source and mechanism of urinary appearance of SAA1/HP/LCN15 remain unclear and note that Human Protein Atlas suggests liver (SAA1, HP) and GI tract (LCN15) as major production sites, raising the possibility that systemic inflammation or altered glomerular permeability could contribute. While the manuscript argues against "general protein leakage" based on the limited number of serum proteins identified as diagnostic markers, the controls are described as controls but not clearly defined as disease controls, and the validation controls are "healthy blood donors." This design does not adequately address real-world differential diagnosis in urology clinics, where inflammatory and structural renal conditions are common. I would recommend the authors (i) more clearly characterize controls (comorbidities, urinalysis, renal function, inflammatory markers), and (ii) ideally include relevant disease controls or at least discuss how such conditions might impact these proteins and the UrineScore.
3. Biological interpretation: mitochondrial respiration signature and tissue-of-origin vs "cancer-derived" biomarkers
The manuscript reports enrichment of mitochondrial respiration/electron transport chain signatures in urine sediment proteomics, and increased CoQ10 and lipid classes in urine lipidomics; it then proposes that the lipid phenotype may relate to tumor biology, whereas the mitochondrial respiration signal likely derives from shed epithelium or surrounding tissue damage rather than ccRCC cells, which are described as relatively independent of oxidative phosphorylation. This creates a conceptual tension: the stated goal is biomarker discovery for ccRCC diagnosis, but part of the omics signal appears to reflect non-tumor processes (e.g., epithelial shedding, injury response). Even if diagnostically useful, such markers may be less specific to ccRCC and more reflective of kidney injury or local inflammation. The authors should address this more directly and consider additional analyses to strengthen linkage to ccRCC, for example by doing correlation with hematuria, proteinuria, urinary leukocytes, and renal function measures if available.

Minor concerns
1. Inaccurate statement on RCC cell of origin
The introduction states that "RCCs are a heterogenous group of cancers arising from the proximal convoluted tubule." This is not accurate across RCC subtypes: while ccRCC is classically linked to proximal tubular epithelium, other subtypes (e.g., chromophobe RCC) are generally associated with more distal nephron/collecting duct-related lineages. The statement should be

revised to reflect subtype-specific origins.

Referee #3 (Remarks for Author):

The study by Jonsson et al. aims to identify clinically relevant urinary biomarkers for clear cell renal cell carcinoma (ccRCC), an area of clear and unmet clinical need. The concept of developing non-invasive biomarkers for early detection is highly relevant, and the authors report clear signals between healthy individuals and ccRCC patients despite a relatively modest cohort size. However, several issues warrant clarification.
First, the discovery cohort includes a limited number of ccRCC cases (n=22), alongside non-ccRCC (n=9) and healthy controls (n=12). While the reported signals appear robust, the relatively small sample size raises concerns regarding statistical power, generalizability, and potential overfitting. Additional discussion of cohort heterogeneity and robustness of the findings would strengthen the manuscript.
Second, the authors state that the study aims to identify early-stage biomarkers. However, only approximately half of the tumors in the discovery cohort are stage pT1 (13/22), with the remainder consisting of more advanced disease (1 pT2 and 8 pT3). A similar distribution is observed in the validation cohort, where pT1 and pT3 tumors are represented in roughly equal proportions. Given this stage composition, it is unclear whether the identified biomarkers truly reflect early-stage disease biology or are primarily driven by signals from more advanced tumors.
Moreover, the identified biomarkers are compared against healthy controls rather than against patients with small renal masses of indeterminate nature or other urological conditions that may mimic RCC. These cases were excluded from the initial cohort of 40 cases included in the study. This raises questions regarding their specificity and real-world diagnostic applicability. If the intent is to propose these markers for early detection or clinical decision-making, additional analyses stratified by tumor stage and size would be highly informative. In particular, it would be important to demonstrate that the biomarkers can discriminate pT1 tumors from controls independently of higher-stage cases.

The authors perform comprehensive proteomic, lipidomic, and metabolomic profiling of urine samples and report discriminatory signals across all three platforms. However, the proposed diagnostic "UrineScore" is derived solely from the proteomic dataset. The rationale for excluding lipidomic and metabolomic features from the integrated diagnostic model is not sufficiently explained. Given that multi-omics integration is often a key strength in biomarker development, it would be important to clarify whether combined models were explored and, if so, how their performance compared to the proteomics-only approach. An integrative model might further improve robustness, specificity, or generalizability, particularly in small cohorts where single-omics signals may be vulnerable to overfitting.
While the reported performance of the UrineScore appears promising, its stage-specific performance remains unclear. As the stated aim is early detection, it is particularly important to demonstrate that the score retains diagnostic accuracy in low-stage (pT1) disease independently of higher-stage cases. Validation restricted to early-stage tumors would substantially strengthen the translational relevance of the findings.
Overall, while the study addresses an important clinical question and presents promising initial findings, further clarification regarding stage-specific performance and diagnostic applicability is needed to support the claim of early-stage biomarker identification.

Point-by-Point reply to Reviewer 1:

The discovery cohort is relatively small but may be suitable for selecting candidate markers. However, the validation cohort is far too small and was not biometrically planned based on the results of the discovery cohort. Therefore, the actual value of the markers cannot be definitively assessed.

We are grateful to the reviewer for taking the time to review our study and for raising important points of critique which should be investigated further. Below our replies are attached in which we address all of the raised critiques.

The work of Jonsson et al. is dedicated to an interesting and important issue, namely the identification of potential tumor markers for clear cell renal cell carcinoma (ccRCC), for which no suitable biomarkers are currently available. The analysis of such markers in urine appears particularly attractive, as urine samples can be obtained non-invasively and repeatedly without great effort. By applying proteomic, lipidomic, and metabolomic analyses, the authors succeeded in identifying the three protein biomarkers serum amyloid A1 (SAA1), haptoglobin (HP) and lipocalin 15 (LCN15) in urine supernatant as promising candidates. They developed a diagnostic marker score from them that allows ccRCC patients to be distinguished from both healthy controls and patients with another RCC subtype (nccRCC).
Even though this approach appears to be very well thought out and comprehensive, and the complex methodology is described well in detail, the study has certain shortcomings.

We are grateful to the reviewer for the overall positive review of our work and for the constructive and helpful suggestions. All suggested points and raised concerns are listed below with point-to-point comments in blue.

On the one hand, there is no real need for a population-based screening for this type of tumor, but it would be very helpful, for example in patients with hematuria or suspicious renal masses, to confirm the suspected diagnosis with the aid of non-invasively determinable tumor markers. Therefore, control subjects with hematuria or other renal masses appeared to be more suitable than healthy subjects. For that reason, the use of healthy control subjects should be justified more comprehensively, as, in the reviewer's opinion, they do not necessarily represent a suitable control group. In addition, the controls should be matched to the patient cohort at least in terms of age and gender. However, no information on the control subjects is provided here. The authors should follow the reporting guidelines for diagnostic studies and provide at least a flow chart of cohort selection and study design.

We thank the reviewer for this clinically important point. We fully agree that the most diagnostically impactful application of a urine-based kidney cancer biomarker test would be in patients presenting with hematuria, suspicious renal masses, and other urological conidtions where a non-invasive confirmatory tool would have immediate clinical utility. We share this vision and are actively working to validate the UrineScore in such urological patient cohorts as the logical next step in the translational development of this diagnostic. This is now discussed in the Discussion section. Looking ahead, we consider the two most important follow-up priorities to be: (i) expanding overall sample sizes, and (ii) broadening the comparator cohort to include patients with other urological malignancies and benign renal masses, including the nine non-malignant mass patients enrolled in the present study who were excluded from this analysis. Active recruitment toward these goals is ongoing; however, this expanded validation is outside the scope of the current early-phase discovery study.

Regarding the use of healthy controls in the current study: we would like to clarify the scientific rationale for this design decision. The primary objective of this discovery cohort study was proof-of-concept, to establish whether ccRCC-associated signals are detectable and quantifiable in urine, and whether a composite score could achieve robust discrimination of ccRCC from a non-cancer baseline. Healthy controls represent the most stringent comparator for this purpose, as any signals identified against this background are conservative estimates of diagnostic power with minimal confounding from urological comorbidities.

We would also note that patients presenting with non-malignant renal masses (angiomyolipoma, Bosniak cysts, etc; n=9) were enrolled in the patient arm of this study and underwent full pathological assessment but were excluded from analysis on pathological grounds rather than used as controls (see Fig. EV1 for STARD recruitment chart). This reflects a deliberate design choice: including benign mass patients as controls in a discovery cohort could embed confounders specific to mass-related urinary changes into the baseline, which would complicate biomarker interpretation. Their inclusion in the enrolled cohort does, however, demonstrate that the study was not naive to the clinical context of renal mass presentation. Further, due to the heterogenous nature of this group and the large range of other renal conditions which would fall into this group, inclusion of such samples into the study will be done once more samples have been collected.

Regarding cohort reporting: we agree that transparent reporting is essential for diagnostic studies. Age and sex data for all subjects, including controls, have now been added to Table EV1 for the discovery cohort. For the validation cohort, this data was unfortunately not available in the biobank. Furthermore, a clinical trial recruitment flowchart has been included as a new supplementary figure (Fig. EV1), designed in accordance with STARD reporting guidelines for diagnostic accuracy studies. As shown in the flowchart, patient recruitment and control recruitment were conducted as two independent arms: patients presenting with a primary renal mass (n=40 enrolled, n=31 included after pathological exclusion of non-malignant masses) and healthy volunteers with no known urological disease (n=12), age- and sex-matched to the RCC cohorts. The final analysis cohort comprised n=43 subjects (22 ccRCC, 9 nccRCC, 12 healthy controls).

The size of the discovery cohort is not very large, with 22 ccRCC patients and 12 control subjects. In particular, the comparison with only nine nccRCC patients (with only one chromophobe RCC) appears to be very limiting. In any case, the validation cohort is far too small. For a reliable statement, it must be expanded after biometric planning based on the results from the discovery cohort.

We thank the reviewer for this important comment and fully agree that our cohorts are on the smaller side. To expand the analysis to a larger setting, and to look at the behavior of the biomarkers from patients on a global scale, we have opted for analyzing The Cancer Genome Atlas (TCGA) and Clinical Proteome Tumor Analysis Consortium (CPTAC) data from kidney cancer. We have included a completely new main figure (**Fig. 6**) analyzing these publicly available datasets. This data can be found in the new **Fig. 6A** and **Fig. 6C** and is more thoroughly addressed below in the TCGA specific comment.

Regarding the nccRCC comparison, we fully acknowledge that with only nine nccRCC patients, of whom only one has chromophobe RCC, definitive conclusions about subtype specificity cannot be drawn. The nccRCC comparison is therefore presented as exploratory and hypothesis-generating rather than definitive, and we have toned down the associated claims in the revised manuscript accordingly. The TCGA data, which includes substantially larger pRCC

and chRCC cohorts, provides more robust support for the ccRCC-preferential expression pattern of the three markers across RCC subtypes.

As the reviewer states, further recruitment of larger cohorts, including other kidney disease entities and a greater representation of non-ccRCC subtypes, is of high importance for definitive validation. This is unfortunately outside the scope of the current study, but we have now thoroughly expanded our discussion on the limitations of the present study in the Discussion explicitly stating that prospective multicenter validation with biometric sample size planning based on the current results is the necessary and planned next step before clinical translation of the UrineScore.

The statistical performance of the UrineScore is described in detail below, in response to the reviewer's final comment.

The authors draw interesting conclusions from the different protein patterns in urine supernatant and urine sediment. They suspect that electron transport chain phenotypes are linked to healthy, shed epithelium in the urine sediment and that upregulated respiratory chain pathways in the urine sediment most likely come from increased shedding of healthy epithelium or other shed urogenital cells. In this context, it would have been interesting to examine the urine sediments more closely for their cellular composition using urine cytology in order to prove that no or only a few RCC cells are detectable and which non-malignant cells dominate the sediment (epithelial cells, erythrocytes, immune cells?). This would have allowed potential correlations with protein and metabolite patterns to be evaluated.
Nevertheless, the associations between biomarkers at the protein and metabolite level in the various urine compartments (supernatant and cellular sediment) are interesting. Based on this, the authors draw comprehensible and insightful conclusions, which, however, should be examined and verified in greater depth in conjunction with the relevant literature. Overall, the discussion section of the manuscript primarily summarizes the results once again, but does not compare them in depth with those of other similar studies.

The reviewer raises a very important point regarding how urine cytology could be used to more thoroughly evaluate how different urinary compartments might contribute to our observed signatures. This is unfortunately no longer possible for the present cohort(s) since the sediment has already been processed for proteomic analysis, but it is certainly something that we will keep in mind and incorporate for the planned follow-up studies.

Regarding the depth of the discussion section, we thank the reviewer for appreciating our conclusions based on the data at hand and fully agree that the manuscript would greatly benefit from contextualizing these findings more to the current literature. This has now been included in the discussion section. Please also see our more thorough in the discussion-centric critique below.

Since the authors have already used the TCGA data from the relevant RCC cohorts (no group sizes and references are given for these cohorts!) with regard to survival (it remains unclear whether this refers to overall survival), they should also evaluate their identified markers in these RCC cohorts compared to normal tissue. It would also be interesting to examine the prognostic relevance of the three markers for ccRCC based on TCGA data.

We thank the reviewer for pointing out the additionally required details in the survival curve in **Fig. 2A**. We have updated the y-axis label and figure legend to make it clear that the shown survival refers to overall survival. Furthermore, this is indeed survival in the TCGA pan-kidney

cohort which we have now indicated in both the results and the associated figure legend. Furthermore, we have also added the n numbers for each type of included kidney cancer in the figure legend.

We fully agree with the reviewer on the value of comparing our markers to normal tissue. As previously mentioned, we have analyzed the behavior of SAA1, LCN15 and HP in both TCGA and CPTAC data (**Fig. 6A, C**) as well as the prognostic value of the UrineScore (**Fig. 6B, D**). On top of being upregulated in the tumor compartment of ccRCC in the TCGA dataset, a combined signature of SAA1, HP and LCN15 carries prognostic value in ccRCC and predicts the overall survival of the patients with a high signature score associating with reduced survival. Overall, the tumor levels of SAA1, LCN15 and HP are higher in ccRCC compared to pRCC and chRCC, and in neither pRCC nor chRCC did the signature have any significant prognostic value.

Furthermore, in CPTAC a combined signature of SAA1 and HP (LCN15 not detected at the protein level, we have included discussions regarding the discrepancy between mRNA and protein data for LCN15 at length in the Discussion) also predicts survival in this cohort.

With regard to the diagnostic suitability and value of the three identified protein markers, their relevance in relation to ccRCC should also be discussed in more detail. There are a number of studies on SAA1 that demonstrate the prognostic potential of this factor in RCC. There are also reports on haptoglobin that should be examined more closely, e.g., the study by Tolson et al. (PMID: 15107802), which also identified SAA1 and haptoglobin in the blood of ccRCC patients. In the study by Sandim et al. (PMID: 26420021), which the authors briefly cite but do not discuss in detail, haptoglobin was also identified as differentially secreted protein in the urine of ccRCC patients by proteomic analysis. However, this study also identified uromodulin and apolipoprotein D (which serve as reference proteins herein) as differentially secreted proteins, which should be considered in more detail by the authors of this manuscript. In this context, the usefulness of uromodulin (also known as Tamm-Horsfall protein) should also be discussed more thoroughly. This protein is the most abundant protein in urine and shows values in urine supernatant that are orders of magnitude higher than those of the three identified tumor markers (especially in comparison to SAA1 and LCN15), which may level out larger differences. This should be acritically considered.
Several other studies conducted on urine samples using proteomics are mentioned, but their results are not compared with the obtained results. However, this is necessary for a better understanding of the significance of the reported findings. Technical aspects such as analysis and standardization approaches could also be discussed.

We agree with the reviewer that further discussion and contextualizing our results compared to other studies would be of high value. We have therefore amended the discussion accordingly and also further justified our inclusion of uromodulin (UMOD) as a normalization factor.

SAA1 and HP in the RCC literature. We have added a dedicated discussion of the prior literature on SAA1 and HP in RCC. Regarding SAA1, a number of independent studies have demonstrated its prognostic relevance in ccRCC at both the mRNA and protein level. These findings are consistent with our own, new TCGA and CPTAC analyses, in which SAA1 shows significant upregulation in ccRCC tumour tissue compared to normal-adjacent tissue at the mRNA level and at the protein level in CPTAC (please see the new **Fig. 6A, C)**. Regarding HP, Tolson et al. (PMID: 15107802) identified both HP and SAA1 in the serum of renal cell carcinoma patients using SELDI mass spectrometry, proposing them as candidate serum biomarkers and notably detecting multiple SAA1 variants not previously described in cancer

patient sera. The convergent identification of these proteins in both serum and urine across independent cohorts, institutions, and analytical platforms spanning two decades provides compelling cross-matrix validation of their biological relevance to ccRCC that we now discuss explicitly in the revised manuscript.

Sandim et al. and the identification of HP in ccRCC urine. We have substantially expanded our discussion of Sandim et al., which we previously cited without adequate contextualization. This study performed label-free quantitative proteomics on urine from ccRCC patients and identified haptoglobin as a significantly upregulated secreted protein - a finding that directly and independently corroborates our identification of HP as a UrineScore component. We now discuss this convergence explicitly as cross-platform validation of HP as a urinary ccRCC marker.

UMOD and APOD as differentially secreted proteins in Sandim et al. We thank the reviewer for drawing attention to this specific finding, which we had not addressed in the original manuscript. Sandim et al. also reported uromodulin and apolipoprotein D as differentially secreted in ccRCC urine — both of which we use as normalizers in our PRM-MS assay. We have now addressed this directly in the revised Discussion. UMOD was chosen as a normalizer on the basis of its constitutive and abundant expression by epithelial cells of the thick ascending limb of the loop of Henle, its kidney-specific origin, and its status as the most abundant protein in healthy human urine — properties that make it a pragmatic reference protein for controlling sample-to-sample variation in urinary concentration and processing. Its use as a normalizer rather than a candidate marker is therefore a deliberate analytical choice consistent with its established role in urinary proteomics normalization pipelines. While Sandim et al. identified UMOD as differentially secreted in an untargeted discovery proteomics study, this finding has not been independently replicated and may reflect variation in urinary dilution rather than tumor-driven regulation. We acknowledge, however, that if UMOD is genuinely differentially regulated in ccRCC urine, this could theoretically confound our normalization strategy by compressing the observed differences in the three UrineScore markers.

Uromodulin as the most abundant urinary protein. We have added an explicit discussion of the dynamic range challenge posed by UMOD. As the reviewer correctly notes, UMOD is present at concentrations orders of magnitude higher than SAA1 and LCN15 under physiological conditions. This is precisely why we use it as a denominator in the PRM score formula, dividing the summed peptide areas of each marker of interest by the summed peptide areas of UMOD, KLK1 and APOD normalizes each sample's marker abundance to its overall urinary protein content, thereby controlling for dilution differences between samples. The use of a three-protein normalizer panel rather than a single protein further reduces the impact of any individual normalizer fluctuation. We now discuss this normalization rationale explicitly in both the Discussion sections of the revised manuscript.

Comparison with other urine proteomics studies. We have substantially revised the Discussion to provide a detailed comparison of our findings with the broader ccRCC urine proteomics literature, including Sandim et al., Santorelli et al., Chinello et al., Yang et al., and Di Meo et al. None of these studies identified SAA1, HP and LCN15 as a composite diagnostic signature, and critically none validated their findings in an independent cohort using a targeted quantitative assay. The UrineScore therefore represents a methodological advance over these discovery-phase studies: by translating candidate markers identified by DIA proteomics into a targeted PRM-MS assay with explicit cut-off values, defined normalization strategy, and validation in a geographically independent cohort, we move from biomarker discovery towards a framework that is reproducible and prospectively applicable. Beyond the urine proteomics

literature, we have also added a discussion of Freedman et al. (Nature Medicine 2020), who demonstrated that cfMeDIP-seq of urine cell-free DNA can classify RCC patients with high accuracy, contextualizing our proteomic approach within the broader liquid biopsy landscape and noting that epigenomic and proteomic approaches are complementary rather than competing.

Technical aspects and standardization. We have expanded the Methods and Discussion to provide greater detail on the analytical approach and performance evaluations, including the rationale for the scheduled PRM method, the selection of normalization peptides, the use of Skyline for data processing and manual evaluation, and the criteria for peptide inclusion in the PRM inclusion list.

The classification of the markers using the described methodology is comprehensible and considers them independently of their protein concentration in urine supernatant, which varies considerably between the three markers. However, the authors do not propose specific cut-off values for distinguishing between the different groups, meaning that it is not really possible to assess the diagnostic performance (specificity, sensitivity, accuracy, PPV, NPV), even though the AUC values look promising.

We fully concur with the reviewer that additional performance metrics would greatly strengthen the manuscript. We have added a full statistical performance table (**Fig. 5D**, **Table EV4)** reporting sensitivity, specificity, accuracy, positive predictive value (PPV) and negative predictive value (NPV) at each UrineScore threshold (≥1, ≥2, ≥3) in both the Vienna discovery cohort and the Madrid validation cohort. Explicit cut-off values for each marker are now stated in the Methods section, derived from the upper bound of the 95% bootstrapped confidence interval of the median PRM score in discovery controls: HP > 2.50×10^{-2}, SAA1 > 2.67×10^{-9}, and LCN15 > 1.23×10^{-5}. At the optimal operating threshold of UrineScore ≥2, the assay achieves 86.4% sensitivity and 91.7% specificity in the discovery cohort, with specificity maintained at 93.8% in the independent validation cohort. Across all thresholds and both cohorts, the negative predictive value exceeds 99.5% at a realistic population screening prevalence of 1%, calculated via Bayes' theorem, supporting the utility of the UrineScore as a robust rule-out tool. The sensitivity drops from discovery (86.4%) to validation (53.8%) at UrineScore ≥2 is acknowledged as a limitation and is co1.nsistent with the small number of ccRCC cases in the validation cohort (n = 13) rather than a systematic biological difference between cohorts, given that specificity was maintained at across both cohorts.

Finally, the introduction of the abbreviations used should be reviewed in the methods section.

We have more thoroughly and systematically introduced abbreviations in the methods section.

Point-by-Point reply to Reviewer 2:
This is an interesting and technically solid multi-omics study with a pragmatic PRM-MS assay concept. However, before strong claims about screening/diagnosis are justified, the authors should temper conclusions and better address (i) small-cohort performance uncertainty, (ii) specificity against common urologic/renal confounders, and (iii) tissue-of-origin/biological plausibility for the proposed markers and the broader metabolic signatures.

We thank the reviewer for the positive assessment of our manuscript and constructive critiques for its improvement. We have thoroughly revised the entire manuscript and toned down all conclusions, plus we have added extensive additional discussion regarding limitations, shortcomings and the need for larger follow-up studies. We have also addressed all technical comments regarding sample size etc. with additional analysis in the manuscript highlighted below with point-to-point comments in blue.

Jonsson et al. report an integrated urine multi-omics stuy in ccRCC, identifying three urinary protein markers and deriving a PRM-MS-based composite "UrineScore" that discriminates ccRCC from controls. The clinical goal-non-invasive early detection-is important and clearly unmet. The multi-omics approach and targeted PRM workflow are strengths. However, key limitations around cohort size, specificity, and biological interpretability currently constrain confidence in clinical applicability.

Major concerns:
1. Cohort size and robustness of model performance
The cohorts are small, particularly for validation, which raises concerns about overestimation of diagnostic performance and limited generalizability. The discovery cohort includes 22 ccRCC and 12 controls, with an additional nccRCC group (n=9) for some analyses (pRCC n=8; chRCC n=1). The independent validation cohort includes 10 ccRCC and 16 controls (and excludes 3 RCC samples for histology/clinical reasons). Given these sample sizes, the reported AUROC/accuracy (e.g., UrineScore AUROC ~0.96 in discovery and ~0.95 in validation) may be optimistic, and confidence intervals should be emphasized (or added if missing), particularly for sensitivity/specificity at clinically relevant thresholds. The UrineScore construction uses a dichotomization approach relative to the control 95% CI of the median per marker, then sums the binary calls (0-3). This choice can be unstable in small cohorts and is sensitive to control distribution and pre-analytical factors.

We thank the reviewer for this detailed and very important critique. The concern about small cohort sizes is valid and one we share — this is explicitly acknowledged as the primary limitation of the current study. We have addressed each component of this comment systematically below and have made several additions to the revised manuscript in response.

AUROC confidence intervals and full diagnostic performance metrics and explicit cut-off values.
We have added bootstrapped 95% confidence intervals to the UrineScore AUROCs. The discovery AUROC of 0.957 (95% CI: 0.873–1.000) and validation AUROC of 0.949 (95% CI: 0.588–0.941) confirm meaningful discriminatory performance across both cohorts. In direct response to the reviewer's concern that AUROCs alone are insufficient for diagnostic assessment, we have added a full diagnostic performance table (Fig. 5D, Table EV4) reporting sensitivity, specificity, accuracy, PPV and NPV at each UrineScore threshold (≥1, ≥2, ≥3) in both cohorts, with Clopper-Pearson 95% CIs for all sensitivity and specificity estimates. Explicit cut-off values for each marker are now stated in the Methods, derived from the upper

bound of the 95% bootstrapped CI of the median control PRM score: HP > 2.50×10^{-2}, SAA1 > 2.67×10^{-9}, and LCN15 > 1.23×10^{-5}.
At the optimal operating threshold of UrineScore ≥2, the assay achieves 86.4% sensitivity (95% CI: 65.1–97.1%) and 91.7% specificity (95% CI: 61.5–99.8%) in the discovery cohort, with specificity maintained at 93.8% (95% CI: 69.8–99.8%) in the validation cohort. The sensitivity drops from 86.4% to 53.8% (95% CI: 18.7–81.3%) at this threshold is acknowledged as a limitation, attributable to the small validation ccRCC group (n = 13) rather than a systematic biological difference. Across all thresholds and both cohorts, the NPV exceeds 99.5% at 1% population prevalence, supporting the UrineScore as a robust rule-out tool. The wide CIs honestly reflect the small sample sizes of this early-phase discovery study, and we have added a statement to the Discussion acknowledging that larger cohorts are required to establish stable performance estimates.

Stability of the dichotomization approach. To directly address the concern about scoring instability, we implemented a continuous logistic regression model using the three log-transformed PRM-MS scores as predictors, making no assumptions about thresholds and using the full continuous information in the data. Given the small discovery cohort (N = 34), we applied leave-one-out cross-validation (LOOCV) — the most conservative cross-validation strategy for small samples — yielding a fully bias-corrected discovery AUC of 0.799 (95% CI: 0.609–0.989). This fixed model was then applied without refitting to the independent Madrid validation cohort, yielding an external validation AUC of 0.750. Critically, the binary UrineScore outperforms the logistic regression model in the validation cohort (AUROC 0.949 vs 0.750), directly contradicting the hypothesis that the binary approach is the less stable method. The zero-inflated distributions of SAA1 and LCN15 in healthy controls, SAA1 undetectable in 10 of 12 discovery controls and LCN15 undetectable in 8 of 12, reflect a biological dichotomy that the binary threshold captures. The full logistic regression results are presented in **Rebuttal Figure 1** below:

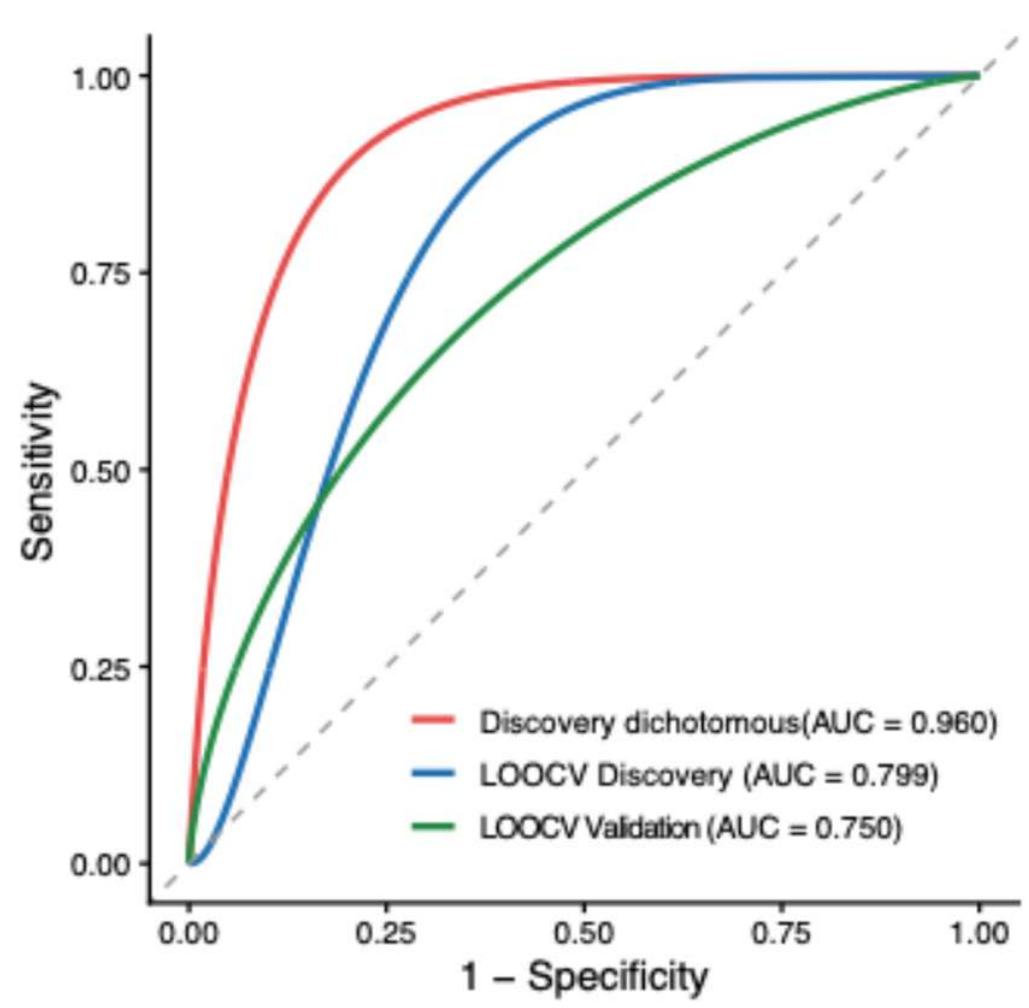

Rebuttal Figure 1. The dichotomous UrineScore outperforms logistic regression analysis in the discovery and validation cohort.

Generalizability and robustness across cohorts. Several aspects of our data argue for the robustness of the findings despite the cohort size. First, the signal is replicated in two geographically independent cohorts recruited at different institutions (Vienna and Madrid) with no overlap in samples, personnel, or processing. Second, the biological validity of the three

markers is now independently supported by TCGA and CPTAC data showing significant upregulation of HP, SAA1 and LCN15 in ccRCC tumour tissue at the mRNA level and HP and SAA1 at the protein level in CPTAC (**Fig. 6A, C)**, providing orthogonal evidence that the urinary signal reflects genuine tumour biology rather than a cohort-specific artefact. Third, the zero-inflated distribution of SAA1 and LCN15 in healthy controls means the discriminatory signal is driven by near-complete absence in controls versus detectable presence in cases — a robust binary biological phenomenon rather than a subtle continuous signal vulnerable to overfitting.

Nonetheless, the reviewer is correct that the sample size is a big concern. We have extended the limitations section of the Discussion to explicitly acknowledge the sensitivity drop from discovery to validation, the wide confidence intervals reflecting small sample sizes, and the need for larger prospective multicentre validation as the necessary next step before clinical translation. We have also added a statement that logistic regression should be evaluated as the preferred scoring framework as cohort sizes increase, while noting that the binary UrineScore demonstrated superior external generalization in the current study.

2. Specificity and confounding by non-malignant renal/urologic conditions
A critical issue for any urine biomarker intended for screening is specificity versus common confounders (hematuria of non-malignant origin, nephrolithiasis, CKD, glomerular disease, inflammatory states). The authors acknowledge that the cellular source and mechanism of urinary appearance of SAA1/HP/LCN15 remain unclear and note that Human Protein Atlas suggests liver (SAA1, HP) and GI tract (LCN15) as major production sites, raising the possibility that systemic inflammation or altered glomerular permeability could contribute. While the manuscript argues against "general protein leakage" based on the limited number of serum proteins identified as diagnostic markers, the controls are described as controls but not clearly defined as disease controls, and the validation controls are "healthy blood donors." This design does not adequately address real-world differential diagnosis in urology clinics, where inflammatory and structural renal conditions are common. I would recommend the authors (i) more clearly characterize controls (comorbidities, urinalysis, renal function, inflammatory markers), and (ii) ideally include relevant disease controls or at least discuss how such conditions might impact these proteins and the UrineScore.

We thank the reviewer for these critical comments. To address the issue regarding the localization of SAA1, HP and LCN15 expression we have utilized publicly available The Cancer Genome Atlas (TCGA) and Clinical Proteome Tumor Analysis Consortium (CPTAC) data, please see the new **Fig. 6**. First, the markers were upregulated on both RNA level (TCGA) and protein level (CPTAC; LCN15 was not detected at the protein level in tumor samples. However, the absence of LNC15 protein in the CPTAC data could be explained by the fact that LCN15 is readily secreted; thus, not detecting it at the protein level might reflect its secretion). These results indicate that urinary levels of SAA1, HP and LCN15 in ccRCC samples are due to their upregulation in tumors; of course, sources outside the tumor cannot excluded. Furthermore, the TCGA data also indicates cancer specificity in this finding since the upregulation of the 3 markers in papillary RCC is much milder compared to ccRCC, and in chromophobe RCC the upregulation is not detected at all.

Regarding the benefit of more clearly defining the control population and recruiting disease controls, we fully agree with the reviewer that more systematically assessing the levels of the 3 diagnostic markers in general inflammatory conditions (e.g. nephritis) and other renal conditions (e.g. cysts, angiomyolipomas, oncozytomas, etc.) would be of importance for final conclusive proof of the ccRCC specificity of the UrineScore. At the current state, more

information has been added regarding the healthy controls in the discovery cohort to show that they are age matched and sex matched to the ccRCC and nccRCC cohorts. Furthermore, we have included a recruitment chart for patients and controls into the new version of the manuscript (**Fig. EV1**) For follow-up studies, however, we are recruiting patients with more mixed renal conditions to build larger and more high-confidence models to identify specific subtypes of renal malignancy. We do however still believe that our study is of high value to the field since conventional liquid biopsy approaches looking at cell free DNA have had mixed results and kidney tumors tend to be low shedders, and expanding the scope to include multi-omics approaches is of value. Having shown in the TCGA and CPTAC cohorts that the targets of interest indeed are expressed in tumor tissue, and to higher levels than surrounding tissue, does provide more confidence to the tumor specificity of our markers, but as pointed out, this needs to be more clearly demonstrated in clinical cohorts designed to express exactly this. This is, however, outside of the scope of the current study.
Lastly, the discussion has been greatly expanded to clearly highlight these limitations of the present study and openly discusses the need for more rigorous and urologically relevant control groups in future follow ups.

3. Biological interpretation: mitochondrial respiration signature and tissue-of-origin vs "cancer-derived" biomarkers
The manuscript reports enrichment of mitochondrial respiration/electron transport chain signatures in urine sediment proteomics, and increased CoQ10 and lipid classes in urine lipidomics; it then proposes that the lipid phenotype may relate to tumor biology, whereas the mitochondrial respiration signal likely derives from shed epithelium or surrounding tissue damage rather than ccRCC cells, which are described as relatively independent of oxidative phosphorylation. This creates a conceptual tension: the stated goal is biomarker discovery for ccRCC diagnosis, but part of the omics signal appears to reflect non-tumor processes (e.g., epithelial shedding, injury response). Even if diagnostically useful, such markers may be less specific to ccRCC and more reflective of kidney injury or local inflammation. The authors should address this more directly and consider additional analyses to strengthen linkage to ccRCC, for example by doing correlation with hematuria, proteinuria, urinary leukocytes, and renal function measures if available.

We thank the reviewer for this further insightful comment which adds to the criticism raised in the previous question. It is indeed possible that confounding kidney co-morbidities are contributing to the observed data. Analysis of further clinical data from the present cohorts is unfortunately not possible but will be included in future studies. That being said, using the publicly available TCGA and CPTAC cohorts we have now shown a clear upregulation of SAA1, HP and LCN15 at the RNA level and SAA1 and HP at the protein level in tumors compared to normal tissue (see the new **Fig. 6**). This of course does not eliminate the effect of confounding co-morbidities, but it does point to an increase in tumor-derived secreted HP, SAA1 and LCN15 in ccRCC.

Furthermore, within our discovery cohort, we have new added analysis in **Fig. EV5E-H** showing that different UrineScores did not correlate with various clinical parameters, such as overall urine protein concentration (**Fig. EV5E**), immune activation as determined through leukocyte counts in blood (**Fig. EV5F**), kidney function measured via creatinine levels in the blood (**Fig. EV5G**), nor overall inflammation as measured through C-reactive protein in blood (**Fig. EV5H**).

Minor concerns
1. Inaccurate statement on RCC cell of origin

The introduction states that "RCCs are a heterogenous group of cancers arising from the proximal convoluted tubule." This is not accurate across RCC subtypes: while ccRCC is classically linked to proximal tubular epithelium, other subtypes (e.g., chromophobe RCC) are generally associated with more distal nephron/collecting duct-related lineages. The statement should be revised to reflect subtype-specific origins.

We thank the reviewer for this comment and have amended the introduction accordingly.

Point-by-Point reply to Reviewer 3:

The study by Jonsson et al. aims to identify clinically relevant urinary biomarkers for clear cell renal cell carcinoma (ccRCC), an area of clear and unmet clinical need. The concept of developing non-invasive biomarkers for early detection is highly relevant, and the authors report clear signals between healthy individuals and ccRCC patients despite a relatively modest cohort size.

However, several issues warrant clarification.

We thank the reviewer for highlighting the clinical relevance of our study and for the constructive suggestions that helped improve the manuscript. We have thoroughly revised the manuscript and are pleased to provide detailed point-by-point responses to all raised concerns below; our comments in indicated in blue.

First, the discovery cohort includes a limited number of ccRCC cases (n=22), alongside non-ccRCC (n=9) and healthy controls (n=12). While the reported signals appear robust, the relatively small sample size raises concerns regarding statistical power, generalizability, and potential overfitting. Additional discussion of cohort heterogeneity and robustness of the findings would strengthen the manuscript.

Second, the authors state that the study aims to identify early-stage biomarkers. However, only approximately half of the tumors in the discovery cohort are stage pT1 (13/22), with the remainder consisting of more advanced disease (1 pT2 and 8 pT3). A similar distribution is observed in the validation cohort, where pT1 and pT3 tumors are represented in roughly equal proportions. Given this stage composition, it is unclear whether the identified biomarkers truly reflect early-stage disease biology or are primarily driven by signals from more advanced tumors. Moreover, the identified biomarkers are compared against healthy controls rather than against patients with small renal masses of indeterminate nature or other urological conditions that may mimic RCC. These cases were excluded from the initial cohort of 40 cases included in the study. This raises questions regarding their specificity and real-world diagnostic applicability. If the intent is to propose these markers for early detection or clinical decision-making, additional analyses stratified by tumor stage and size would be highly informative. In particular, it would be important to demonstrate that the biomarkers can discriminate pT1 tumors from controls independently of higher-stage cases.

We thank the reviewer for these insightful comments, and we would like to address both point 1 and point 2 below:

We fully agree with the reviewer that the claims that these markers are true early-stage diagnostic markers for ccRCC are not fully supported at this time. We have therefore toned down these claims throughout the entire manuscript, focusing more on general biomarker discovery. To address the concern about statistical robustness more broadly, we have added bootstrapped 95% confidence intervals to the reported UrineScore AUROCs in the revised manuscript. The discovery AUROC of 0.957 (95% CI: 0.873–1.000) and validation AUROC of 0.949 (95% CI: 0.588–0.941) confirm that discriminatory performance is maintained across both cohorts, with the wide confidence intervals directly reflecting the limited sample sizes. We have also added a full diagnostic performance table (**Fig. 5D, Table EV4)** reporting sensitivity, specificity, accuracy, PPV and NPV at each UrineScore threshold in both cohorts. At the optimal operating threshold of UrineScore ≥2, the assay achieves 86.4% sensitivity and 91.7% specificity in the discovery cohort, with specificity maintained at 93.8% in the independent validation cohort. Across all thresholds and both cohorts, the negative predictive value exceeds 99.5% at a realistic population screening prevalence of 1%, calculated via Bayes' theorem, supporting the utility of the UrineScore as a rule-out tool. The sensitivity drop from discovery (86.4%) to validation (53.8%) at UrineScore ≥2 is acknowledged as a limitation and is

consistent with the small number of ccRCC cases in the validation cohort (N = 13) rather than a systematic biological difference between cohorts, given that specificity was well-maintained across both cohorts.

What we have however done to further support our claims is to perform stage specific analyses. We have shown that the overall distribution of the UrineScore is not significantly different between stage 1 (pT1) and stage 3 (pT3) ccRCC tumors in our discovery cohort, **Fig. EV5D**. While we acknowledge that demonstrating stage-specific urine diagnostic performance in our own cohort is not statistically robust at current sample sizes — subgroup analyses with fewer than 13 cases per stage would yield confidence intervals spanning the full 0–1 range and would therefore be uninformative, the consistency of the UrineScore distribution across stages provides initial evidence that the signal is not exclusively driven by advanced disease. This is of course subject to more thorough validation in larger, independent clinical cohorts and we have thus added extensive discussions about the current limitations of the UrineScore and the need for further clinical validation.

Regarding the comparison against patients with small renal masses of indeterminate nature or other urological conditions that may mimic RCC, we fully agree with the reviewer that this represents an important gap that limits assessment of real-world diagnostic specificity. We would also note that patients presenting with non-malignant renal masses (angiomyolipoma, Bosniak cysts, etc; n=9) were enrolled in the patient arm of this study and underwent full pathological assessment but were excluded from analysis on pathological grounds rather than used as controls (see **Fig. EV1** for STARD recruitment chart). This reflects a deliberate design choice: including benign mass patients as controls in a discovery cohort could embed confounders specific to mass-related urinary changes into the baseline, which would complicate biomarker interpretation. Their inclusion in the enrolled cohort does, however, demonstrate that the study was not naive to the clinical context of renal mass presentation. Further, due to the heterogenous nature of this group and the large range of other renal conditions which would fall into this group, inclusion of such samples into the study will be done once more samples have been collected.
We have however added a dedicated limitations paragraph to the Discussion explicitly acknowledging this point and noting that future prospective validation studies should prioritise the recruitment of clinically relevant disease controls, including patients presenting with hematuria or incidentally detected renal masses, to establish the specificity of the UrineScore in the differential diagnostic setting in which it would ultimately be applied.

The authors perform comprehensive proteomic, lipidomic, and metabolomic profiling of urine samples and report discriminatory signals across all three platforms. However, the proposed diagnostic "UrineScore" is derived solely from the proteomic dataset. The rationale for excluding lipidomic and metabolomic features from the integrated diagnostic model is not sufficiently explained.
Given that multi-omics integration is often a key strength in biomarker development, it would be important to clarify whether combined models were explored and, if so, how their performance compared to the proteomics-only approach. An integrative model might further improve robustness, specificity, or generalizability, particularly in small cohorts where single-omics signals may be vulnerable to overfitting.

This is an excellent suggestion and one we have now directly addressed. We constructed an integrated model combining the three protein PRM-MS scores with the four best-performing phosphatidylcholine lipid species identified in our lipidomics screen (PC 38:3, PC 38:4, PC 38:5, PC 38:6) — selected on the basis of their prior reported association with ccRCC urine

lipidomics. A logistic regression model incorporating all seven features was evaluated using leave-on-out cross-validation (LOOCV) in the discovery cohort and compared to lipid-only and protein-only models. Metabolomics was excluded from the integrated model, as discussed in the Result section.

The results, shown in the new **Fig. EV5B-C**, demonstrate that the integrated model achieves a LOOCV AUC of 0.883, compared to 0.879 for proteins only and 0.788 for lipids only. While the integrated model numerically improves upon the protein-only model, this difference is not statistically significant (DeLong test $p = 0.957$), which we attribute to the limited statistical power at $n = 34$ with 7 predictors. Principal component analysis of the integrated feature matrix confirms that lipid and protein features capture partially complementary variance (PC1 = 68%), suggesting that multi-modal integration has biological rationale and may yield greater benefit in larger prospective cohorts.

The rationale for basing the primary UrineScore on proteomics alone is threefold: (i) the protein markers show the strongest and most consistent individual discrimination; (ii) the targeted PRM-MS assay is analytically simpler, more reproducible, and more readily translatable to a clinical workflow than a combined proteomics-lipidomics platform; and (iii) the integrated model does not achieve statistically superior performance at the current sample size. We agree with the reviewer that integrated multi-omics scoring should be a priority for future validation studies and have added a statement to this effect in the Discussion.

While the reported performance of the UrineScore appears promising, its stage-specific performance remains unclear. As the stated aim is early detection, it is particularly important to demonstrate that the score retains diagnostic accuracy in low-stage (pT1) disease independently of higher-stage cases. Validation restricted to early-stage tumors would substantially strengthen the translational relevance of the findings.
Overall, while the study addresses an important clinical question and presents promising initial findings, further clarification regarding stage-specific performance and diagnostic applicability is needed to support the claim of early-stage biomarker identification.

We thank the reviewer for this important comment. As discussed in our response to Comment 1 and 2 above, stage-stratified performance analysis in our urine cohorts is not statistically feasible at the current sample sizes, with 13 pT1 cases in the discovery cohort and approximately equal numbers of pT1 and pT3 cases in the validation cohort. Hence, subgroup AUROCs would have confidence intervals spanning the full 0–1 range and would therefore be uninformative rather than strengthening the conclusions.

What we can demonstrate, however, is that the UrineScore distribution is not significantly different between pT1 and pT3 tumours in our discovery cohort (**Fig. EV5D**), providing initial evidence that the composite signal is not exclusively driven by advanced-stage disease. We acknowledge this is not a substitute for a properly powered stage-stratified analysis and have been careful to frame the UrineScore accordingly throughout the revised manuscript, as a general ccRCC diagnostic tool at this stage of development rather than a validated early-detection biomarker. The early-stage claim has been substantially toned down throughout the manuscript, and we have added a dedicated limitations statement explicitly acknowledging that demonstration of stage-specific diagnostic performance requires dedicated prospective recruitment of early-stage patients as a key objective of planned larger follow-up studies.

We fully agree with the reviewer that validation in a larger cohort enriched for pT1 disease, ideally including patients with small incidentally detected renal masses, is the critical next step

before any early-detection claims can be made with confidence, and this is now stated explicitly as the primary objective of our ongoing prospective recruitment effort.

9th Jul 2026

Dear Dr. Penninger,

Thank you for submitting your revised manuscript, which has now been evaluated by the three initial referees. As you will see below, they are satisfied with the revisions, and I will therefore be able to accept your manuscript once the following editorial points have been addressed:

1/ Manuscript text:
- Please remove the highlighted text and only keep in track changes any new modification to the text.
- As also mentioned by referee #3, please carefully check and edit your text where needed.
- Correct the order and headings of the manuscript sections to: Abstract, Introduction, The Paper Explained, Results, Discussion, Methods, Acknowledgements, Disclosure and competing interests statement, References, Figure legends, Expanded View Figure legends
- Materials and methods should be renamed Methods.
- BioRender: remove info from figure legends and instead add a dedicated section to the Methods, using this format:
Graphics:
(some of the... OR Figure #... OR synopsis) Graphics were created with BioRender.com.
- Data Availability: remove "All processed omics data (proteomics from urine supernatants, proteomics from urine sediments, lipidomics and metabolomics) are directly available as expanded view datasets with this publication (Dataset EV1-4)." Provide a specific URL for PXD078983 dataset. Note that all datasets must publicly available before publication of the manuscript.
- References: please correct the format to 10 author names listed before et al.

2/ Figures:
- Remove the EV table legends from the manuscript text and add them to the corresponding tables.
- Please make the appendix figure an EV figure and add the legend to the manuscript text
- In the figure legends, please correct the nomenclature to Figure EV1 etc.
- Please address the queries from our data editors in the figure legends:
1. Please note that the box plots need to be defined in terms of minima, maxima, centre, bounds of box and whiskers, and percentile in the legend of figure 6C
2. Please note that information related to n is missing in the legends of figures EV2 C-E
3. For n=2, please remove statistics and error bars, and only indicate individual data points.

3/ Please upload the Source Data as 1 file per figure.

4/ I introduced minor edits in your synopsis, please let me know if you agree or amend as you see fit:

Urine multi-omics profiling of ccRCC patients identifies a three-protein diagnostic signature (HP, SAA1, and LCN15) that forms a composite UrineScore with 96% accuracy in distinguishing clear cell renal cell carcinoma from healthy controls, validated in an independent cohort.
• Integrated urine proteomics, lipidomics, and metabolomics identified widespread metabolic dysregulation in ccRCC.
• Three urinary proteins-Haptoglobin (HP), Serum Amyloid A1 (SAA1), and Lipocalin 15 (LCN15)-were identified as specifically elevated in ccRCC compared with healthy controls and non-clear cell RCC subtypes.
• A targeted parallel reaction monitoring mass spectrometry (PRM-MS) assay quantified HP, SAA1, and LCN15, and the resulting UrineScore achieved AUROCs of 0.96 and 0.95 in the discovery and validation cohorts, respectively.
• Tumor-specific upregulation of HP, SAA1, and LCN15 was confirmed in TCGA RNA-sequencing data and CPTAC tissue proteomics; combined high expression predicted worse overall survival in ccRCC.
Please resize the visual abstract to 550 px wide x 300 px high.

5/ As part of the EMBO Publications transparent editorial process initiative (see our Editorial at http://embomolmed.embopress.org/content/2/9/329), EMBO Molecular Medicine will publish online a Review Process File (RPF) to accompany accepted manuscripts.
This file will be published in conjunction with your paper and will include the anonymous referee reports, your point-by-point response and all pertinent correspondence relating to the manuscript. Let us know whether you agree with the publication of the RPF and as here, if you want to remove or not any figures from it prior to publication.
Please note that the Authors checklist will be published at the end of the RPF.

I look forward to receiving your revised manuscript.

Yours sincerely,

Lise Roth

Lise Roth, PhD
Senior Editor
EMBO Molecular Medicine

***** Reviewer's comments *****

Referee #1 (Remarks for Author):

The authors have addressed the reviewers' criticisms in detail and appropriately and discussed them in their letter. The manuscript has been thoroughly revised and adapted; new figures have been created, and, in particular, the discussion section has been completely restructured. The main limitation of the study-namely, the very small sample size-has not been altered, but has been appropriately addressed in the revision.

Referee #2 (Comments on Novelty/Model System for Author):

the data presented are preliminary in nature. the goal of the manuscript is to develop biomarkers for early diagnosis of ccRCC. It is not presented as a multi-omics analysis with the potential to identify biomarkers. thus, although the study is technically sound the limited number of samples is an issue. the authors used CPTAC to demonstrate that their proteins of interest are expressed in cancer cells but this do not adress the issue of specificity of these biomarkers.

Referee #2 (Remarks for Author):

We would like to thank the authors for their efforts to answer the questions raised and for analysis of CPTAC data. However, as this stated by the authors, this does not eliminate the effect of confounding co-morbidities. We thank them for adding these limitations in the discussion

Referee #3 (Comments on Novelty/Model System for Author):

The authors have addressed my main concerns adequately. They have appropriately toned down the early-detection claims, added further discussion of cohort size and diagnostic limitations, provided additional robustness analyses, clarified the lack of stage-specific power, and added rationale for retaining a proteomics-based UrineScore rather than a fully integrated multi-omics model.

I do not think further experimental work is required for publication.

Referee #3 (Remarks for Author):

I thank the authors for their detailed and constructive response. The revised manuscript appears to address my main concerns. In particular, the early-stage biomarker claims have been appropriately moderated, the limitations regarding cohort size, disease controls, and stage-specific diagnostic performance are now more clearly acknowledged, and the rationale for the proteomics-based UrineScore is better justified.

I therefore have no further major concerns and consider the manuscript suitable for publication. I only recommend checking for minor numerical or typographical inconsistencies before final acceptance.

The authors addressed the remainining editorial issues.

22nd Jul 2026

Dear Dr. Penninger,

Thank you for submitting your revised files. I am pleased to inform you that your manuscript is accepted for publication and is now being sent to our publisher to be included in the next available issue of EMBO Molecular Medicine.

Your manuscript will be processed for publication by EMBO Press. It will be copy edited and you will receive page proofs prior to publication. Please note that you will be contacted by Springer Nature Author Services to complete licensing and payment information.

You may qualify for financial assistance for your publication charges - either via a Springer Nature fully open access agreement or an EMBO initiative. Check your eligibility: https://link.springer.com/journal/44321/how-to-publish-with-us

Should you be planning a Press Release on your article, please notify the production team via this form in order to coordinate publication and release dates: https://forms.cloud.microsoft/e/eH4A8JVP6g

If you have any questions, please do not hesitate to contact the Editorial Office. Thank you for your contribution to EMBO Molecular Medicine.

Yours sincerely,

Lise Roth

Lise Roth, Ph.D
Senior Editor
EMBO Molecular Medicine

>>> Please note that it is EMBO Molecular Medicine policy for the transcript of the editorial process (containing referee reports and your response letter) to be published as an online supplement to each paper. If you do NOT want this, you will need to inform the Editorial Office via email immediately. More information is available here: https://link.springer.com/partners/embo-press/editorial-policies#Peer%20review

EMBO Press Author Checklist

Corresponding Author Name: Gustav Jonsson, Josef M. Penninger	
Journal Submitted to: EMBO Molecular Medicine	
Manuscript Number: EMM-2026-23297	

USEFUL LINKS FOR COMPLETING THIS FORM
The EMBO Journal - Author Guidelines
EMBO Reports - Author Guidelines
Molecular Systems Biology - Author Guidelines
EMBO Molecular Medicine - Author Guidelines

Reporting Checklist for Life Science Articles (updated January
This checklist is adapted from Materials Design Analysis Reporting (MDAR) Checklist for Authors. MDAR establishes a minimum set of requirements in transparent reporting in the life sciences (see Statement of Task: 10.31222/osf.io/9sm4x). Please follow the journal's guidelines in preparing your manuscript.
Please note that a copy of this checklist will be published alongside your article.

Abridged guidelines for figures
1. Data
The data shown in figures should satisfy the following conditions:
- the data were obtained and processed according to the field's best practice and are presented to reflect the results of the experiments in an accurate and unbiased manner.
- ideally, figure panels should include only measurements that are directly comparable to each other and obtained with the same assay.
- plots include clearly labeled error bars for independent experiments and sample sizes. Unless justified, error bars should not be shown for technical
- if n<5, the individual data points from each experiment should be plotted. Any statistical test employed should be justified.
- Source Data should be included to report the data underlying figures according to the guidelines set out in the authorship guidelines on Data

2. Captions
Each figure caption should contain the following information, for each panel where they are relevant:
- a specification of the experimental system investigated (eg cell line, species name).
- the assay(s) and method(s) used to carry out the reported observations and measurements.
- an explicit mention of the biological and chemical entity(ies) that are being measured.
- an explicit mention of the biological and chemical entity(ies) that are altered/varied/perturbed in a controlled manner.
- the exact sample size (n) for each experimental group/condition, given as a number, not a range;
- a description of the sample collection allowing the reader to understand whether the samples represent technical or biological replicates (including how many animals, litters, cultures, etc.).
- a statement of how many times the experiment shown was independently replicated in the laboratory.
- definitions of statistical methods and measures:
  - common tests, such as t-test (please specify whether paired vs. unpaired), simple χ2 tests, Wilcoxon and Mann-Whitney tests, can be unambiguously identified by name only, but more complex techniques should be described in the methods section;
  - are tests one-sided or two-sided?
  - are there adjustments for multiple comparisons?
  - exact statistical test results, e.g., P values = x but not P values < x;
  - definition of 'center values' as median or average;
  - definition of error bars as s.d. or s.e.m.

Please complete ALL of the questions below.
Select "Not Applicable" only when the requested information is not relevant for your study.

Materials

Newly Created Materials	Information included in the manuscript?	In which section is the information available? (Reagents and Tools Table, Materials and Methods, Figures, Data Availability Section)
New materials and reagents need to be available; do any restrictions apply?	Not Applicable	

Antibodies	Information included in the manuscript?	In which section is the information available? (Reagents and Tools Table, Materials and Methods, Figures, Data Availability Section)
For **antibodies** provide the following information:<br>- Commercial antibodies: RRID (if possible) or supplier name, catalogue number and or/clone number<br>- Non-commercial: RRID or citation	Not Applicable	

DNA and RNA sequences	Information included in the manuscript?	In which section is the information available? (Reagents and Tools Table, Materials and Methods, Figures, Data Availability Section)
**Short novel DNA or RNA including primers, probes**: provide the sequences.	Not Applicable	

Cell materials	Information included in the manuscript?	In which section is the information available? (Reagents and Tools Table, Materials and Methods, Figures, Data Availability Section)
**Cell lines:** Provide species information, strain. Provide accession number in repository **OR** supplier name, catalog number, clone number, and/**OR** RRID.	Not Applicable	
**Primary cultures:** Provide species, strain, sex of origin, genetic modification status.	Not Applicable	
Report if the cell lines were recently **authenticated** (e.g., by STR profiling) and tested for mycoplasma contamination.	Not Applicable	

Experimental animals	Information included in the manuscript?	In which section is the information available? (Reagents and Tools Table, Materials and Methods, Figures, Data Availability Section)
**Laboratory animals or Model organisms:** Provide species, strain, sex, age, genetic modification status. Provide accession number in repository **OR** supplier name, catalog number, clone number, **OR** RRID.	Not Applicable	
**Animal observed in or captured from the field:** Provide species, sex, and age where possible.	Not Applicable	
Please detail **housing and husbandry conditions**.	Not Applicable	

Plants and microbes	Information included in the manuscript?	In which section is the information available? (Reagents and Tools Table, Materials and Methods, Figures, Data Availability Section)
**Plants:** provide species and strain, ecotype and cultivar where relevant, unique accession number if available, and source (including location for collected wild specimens).	Not Applicable	
**Microbes:** provide species and strain, unique accession number if available, and source.	Not Applicable	

Human research participants	Information included in the manuscript?	In which section is the information available? (Reagents and Tools Table, Materials and Methods, Figures, Data Availability Section)
If collected and within the bounds of privacy constraints report on age, sex and gender or ethnicity for all study participants.	Yes	Age and sex of participants in the disocvery cohort are available in Table EV1.

Core facilities	Information included in the manuscript?	In which section is the information available? (Reagents and Tools Table, Materials and Methods, Figures, Data Availability Section)
If your work benefited from core facilities, was their service mentioned in the acknowledgments section?	Yes	All core facilities have been acknowledged in the "Acknowledgement" section

Design

Study protocol	Information included in the manuscript?	In which section is the information available? (Reagents and Tools Table, Materials and Methods, Figures, Data Availability Section)
If study protocol has been **pre-registered, provide DOI in the manuscript**. For clinical trials, provide the trial registration number **OR** cite DOI.	Not Applicable	

Report the **clinical trial registration number** (at ClinicalTrials.gov or equivalent), where applicable.	Not Applicable	

**Laboratory protocol**	**Information included in the manuscript?**	**In which section is the information available?** (Reagents and Tools Table, Materials and Methods, Figures, Data Availability Section)
Provide DOI OR other citation details if **external detailed step-by-step protocols** are available.	Not Applicable	

**Experimental study design and statistics**	**Information included in the manuscript?**	**In which section is the information available?** (Reagents and Tools Table, Materials and Methods, Figures, Data Availability Section)
Include a statement about **sample size** estimate even if no statistical methods were used.	Yes	The information is available in Materials and Methods, under "Patient cohorts". Sample sizes were not pre-determined, but rather allocated to groups depending on pathological assessment.
Were any steps taken to minimize the effects of subjective bias when allocating animals/samples to treatment (e.g. **randomization procedure**)? If yes, have they been described?	Not Applicable	
Include a statement about **blinding** even if no blinding was done.	Yes	The information is available in Materials and Methods, under "Patient cohorts". "For all downstream experiments and analysis, experimenters were blinded to the disease or control state of individual samples (single blinding)."
Describe **inclusion/exclusion criteria** if samples or animals were excluded from the analysis. Were the criteria pre-established?<br><br>If sample or data points were omitted from analysis, report if this was due to attrition or intentional exclusion and provide justification.	Yes	The information is available in Materials and Methods, under "Patient cohorts". Renal masses determined to not be renal cell carcinoma were excluded after pathological assessment.
For every figure, are **statistical tests** justified as appropriate? Do the data meet the assumptions of the tests (e.g., normal distribution)? Describe any methods used to assess it. Is there an estimate of variation within each group of data? Is the variance similar between the groups that are being statistically compared?	Yes	All statistical methods are detailed in throughout the Materials and Methods section, and specificalyl collated under "Statistical analysis"

**Sample definition and in-laboratory replication**	**Information included in the manuscript?**	**In which section is the information available?** (Reagents and Tools Table, Materials and Methods, Figures, Data Availability Section)
In the figure legends: state number of times the experiment was **replicated** in laboratory.	Not Applicable	
In the figure legends: define whether data describe **technical or biological replicates**.	Yes	The numbers of biological replicates (number of patients per group) are stated in each figure legend and materials and methods.

Ethics

**Ethics**	**Information included in the manuscript?**	**In which section is the information available?** (Reagents and Tools Table, Materials and Methods, Figures, Data Availability Section)
Studies involving **human participants**: State details of **authority granting ethics approval** (IRB or equivalent committee(s), provide reference number for approval.	Yes	The information is available in Materials and Methods, under "Patient cohorts".
Studies involving **human participants**: Include a statement confirming that **informed consent** was obtained from all subjects and that the experiments conformed to the principles set out in the WMA Declaration of Helsinki and the Department of Health and Human Services Belmont Report.	Yes	The information is available in Materials and Methods, under "Patient cohorts".
Studies involving **human participants:** For publication of **patient photos**, include a statement confirming that consent to publish was obtained.	Not Applicable	
Studies involving experimental **animals**: State details of **authority granting ethics approval** (IRB or equivalent committee(s), provide reference number for approval. Include a statement of compliance with ethical regulations.	Not Applicable	
Studies involving **specimen and field samples:** State if relevant **permits** obtained, provide details of authority approving study; if none were required, explain why.	Not Applicable	

**Dual Use Research of Concern (DURC)**	**Information included in the manuscript?**	**In which section is the information available?** (Reagents and Tools Table, Materials and Methods, Figures, Data Availability Section)
Could your study fall under dual use research restrictions? Please check biosecurity documents and list of **select agents and toxins** (CDC): https://www.selectagents.gov/sat/list.htm	Not Applicable	
If you used a select agent, is the security level of the lab appropriate and reported in the manuscript?	Not Applicable	
If a study is subject to dual use research of concern regulations, is the name of the **authority granting approval and reference number** for the regulatory approval provided in the manuscript?	Not Applicable	

Reporting

The MDAR framework recommends adoption of discipline-specific guidelines, established and endorsed through community initiatives. Journals have their own policy about requiring specific guidelines and recommendations to complement MDAR.

**Adherence to community standards**	**Information included in the manuscript?**	**In which section is the information available?** (Reagents and Tools Table, Materials and Methods, Figures, Data Availability Section)
State if relevant guidelines or checklists (e.g., **ICMJE, MIBBI, ARRIVE, PRISMA**) have been followed or provided.	Not Applicable	
For **tumor marker prognostic studies,** we recommend that you follow the **REMARK** reporting guidelines (see link list at top right). See author guidelines, under 'Reporting Guidelines'. Please confirm you have followed these guidelines.	Yes	Available in Materials and Methods, Figure EV1, Table EV1 (STARD recruitment chart) and Table EV4 (Specificity and Sensitivity assessment with 95% CI).
For **phase II and III randomized controlled trials**, please refer to the **CONSORT** flow diagram (see link list at top right) and submit the CONSORT checklist (see link list at top right) with your submission. See author guidelines, under 'Reporting Guidelines'. Please confirm you have submitted this list.	Not Applicable	

Data Availability

**Data availability**	**Information included in the manuscript?**	**In which section is the information available?** (Reagents and Tools Table, Materials and Methods, Figures, Data Availability Section)
Have **primary datasets** been deposited according to the journal's guidelines (see 'Data Deposition' section) and the respective accession numbers provided in the Data Availability Section?	Yes	Data Availability Section. Proteomics data have been deposited into the PRIDE database, and metabolomics and lipidomics have been deposited into the MetaboLights database. Accession numbers are available in the Data Availability section.
Were **human clinical and genomic datasets** deposited in a public access-controlled repository in accordance to ethical obligations to the patients and to the applicable consent agreement?	Not Applicable	
Are **computational models** that are central and integral to a study available without restrictions in a machine-readable form? Were the relevant accession numbers or links provided?	Not Applicable	
If publicly available data were reused, provide the respective **data citations in the reference list**.	Not Applicable	
